# Genome‑wide identification and expression analysis of the *UBC* gene family in wheat (*Triticum aestivum* L.)

**DOI:** 10.1186/s12870-024-05042-3

**Published:** 2024-04-26

**Authors:** Weidong Gao, Long Zhang, Yanyan Zhang, Peipei Zhang, Fahimeh Shahinnia, Tao Chen, Delong Yang

**Affiliations:** 1 State Key Laboratory of Aridland Crop Science, Gansu Agricultural University, Lanzhou, 730070 China; 2 College of Life Science and Technology, Gansu Agricultural University, Lanzhou, 730070 China; 3 Bioanalytics Gatersleben, Am Schwabenplan 1b, Seeland, 06466 Germany

**Keywords:** Wheat, *UBC* gene family, Gene expression, Allelic variation, Thousand-kernel weight

## Abstract

**Background:**

Ubiquitination is an important regulatory step of selective protein degradation in the plant UPS (ubiquitin–proteasome system), which is involved in various biological processes in eukaryotes. Ubiquitin-conjugating enzymes play an intermediate role in the process of protein ubiquitination reactions and thus play an essential role in regulating plant growth and response to adverse environmental conditions. However, a genome-wide analysis of the *UBC* gene family in wheat (*Triticum aestivum* L.) has not yet been performed.

**Results:**

In this study, the number, physiochemical properties, gene structure, collinearity, and phylogenetic relationships of *TaUBC* family members in wheat were analyzed using bioinformatics methods. The expression pattern of *TaUBC* genes in different tissues/organs and developmental periods, as well as the transcript levels under abiotic stress treatment, were analyzed using RNA-Seq data and qRT-PCR. Meanwhile, favorable haplotypes of *TaUBC25* were investigated based on wheat resequencing data of 681 wheat cultivars from the Wheat Union Database. The analyses identified a total of 93 TaUBC family members containing a UBC domain in wheat genome. These genes were unevenly distributed across 21 chromosomes, and numerous duplication events were observed between gene members. Based on phylogenetic analysis, the TaUBC family was divided into 13 E2 groups and a separate UEV group. We investigated the expression of *TaUBC* family genes under different tissue/organ and stress conditions by quantitative real-time PCR (qRT-PCR) analysis. The results showed that some *TaUBC* genes were specifically expressed in certain tissues/organs and that most *TaUBC* genes responded to NaCl, PEG6000, and ABA treatment with different levels of expression. In addition, we performed association analysis for the two haplotypes based on key agronomic traits such as thousand-kernel weight (TKW), kernel length (KL), kernel weight (KW), and kernel thickness (KT), examining 122 wheat accessions at three environmental sites. The results showed that *TaUBC25-Hap II* had significantly higher TKW, KL, KW, and KT than *TaUBC25-Hap I*. The distribution analysis of haplotypes showed that *TaUBC25-Hap II* was preferred in the natural population of wheat.

**Conclusion:**

Our results identified 93 members of the *TaUBC* family in wheat, and several genes involved in grain development and abiotic stress response. Based on the SNPs detected in the *TaUBC* sequence, two haplotypes, *TaUBC25-Hap I* and *TaUBC25-Hap II*, were identified among wheat cultivars, and their potential value for wheat breeding was validated by association analysis. The above results provide a theoretical basis for elucidating the evolutionary relationships of the *TaUBC* gene family and lay the foundation for studying the functions of family members in the future.

**Supplementary Information:**

The online version contains supplementary material available at 10.1186/s12870-024-05042-3.

## Introduction

Post-translational protein modifications play an important role in living organisms [1]. Ubiquitination, one of the most common posttranslational modifications of proteins, plays a crucial role in the process of selective protein degradation in plants [2–4]. Protein ubiquitination is involved in various processes, such as photomorphogenesis, vascular differentiation, flower development, phytohormone and light signaling, and biotic and abiotic stress responses [5–8]. The process of protein ubiquitination is mediated by the action of three enzymes, termed ubiquitin-activating enzyme (E1s), ubiquitin-conjugating enzyme (E2s), and ubiquitin-ligating enzyme (E3s) [9]. Ubiquitin-conjugating enzymes facilitate the transfer of activated ubiquitin to substrates or E3 ubiquitin ligases [9, 10], resulting in the formation of polyubiquitin chains on target proteins [11]. Together with the E3s, the E2s determine substrate specificity in the ubiquitination system [12]. The E2 enzyme contains a conserved catalytic domain called the UBC domain. It forms a thioester bond with ATP-activated ubiquitin proteins via a cysteine located at the 85th amino acid [5, 13].

To date, a total of 34, 40, 41, 43, 48, 53, 57, 59, 72, 75, and 83 E2 ubiquitin-conjugating enzyme *UBC* genes have been identified in papaya, longan, *Arabidopsis thaliana*, grape, rice, sorghum, potato, tomato, banana, maize, and *Brassica rapa*, respectively [5, 14–20]. Some *UBC* genes were cloned and functionally validated in different plants, such as soybean, tomato and Arabidopsis [8, 21–24]. Previous research has revealed that *UBC* genes play critical roles in a variety of physiological and developmental processes, including Arabidopsis flowering [25], seedling photomorphogenesis [26], root development [27], and female gametophyte development [28], as well as fruit maturation of tomato [29].

In addition, E2 ubiquitin-conjugating enzyme *UBC* genes are also involved in plant responses to environmental stress such as low temperature, heat, drought, and salt [24]. *LeUBC1*, the first plant *UBC* gene identified and characterized in tomato, was found to be upregulated in expression under high temperature stress, suggesting that UBC proteins can degrade abnormal proteins at high temperatures [21]. Salt stress triggered the expression of *AtUBC32*, which plays an important role in salt stress tolerance in Arabidopsis [22]. In Arabidopsis, *UBC7*, *UBC13*, and *UBC14* are involved in the response to salt stress, oxidative stress, and ABA stress [24]. Overexpression of *GmUBC9* in transgenic Arabidopsi*s* and soybean plants increased drought tolerance, and the transgenic plants exhibited a late flowering phenotype with increased expression of genes related to flowering [8]. Therefore, *UBC* genes play an important regulatory role in plant growth and development and response to abiotic stress [8, 28].

Wheat (*Triticum aestivum* L.) is one of the world's most important cereals, contributing 20% of calories and protein consumed worldwide [30]. Increasing wheat yields is important for global food and nutrition security (FAO, http://faostat.fao.org). Hexaploid wheat has a large genome (~ 17 Gb) consisting of A, B, and D subgenomes derived from two amphiploidization events [31]. The wheat genome contains a large number of repetitive sequences, which has limited the study of multiple gene family functions. Although the *UBC* gene family plays an important role in plant growth and development and the response to abiotic stress, little is known about this family in wheat. Hitherto, the main objectives of this study were to identify and characterize *UBC* family members in wheat and investigate the expression pattern of *TaUBC* genes. Additionally, due to the high expression of *TaUBC25* in grains, association analysis with the *TaUBC25* gene was performed using wheat resequencing data from the WheatUnion database and grain trait data acquired from published studies. [32–36]. Simultaneously, the geographic distribution and frequency of favorable allelic variation of *TaUBC25* were analyzed. Our study aims to provide a theoretical basis for elucidating the physiological and biochemical functions of the E2 ubiquitin-conjugating enzyme *UBC* gene family in wheat.

## Results

### Identification of *UBC* genes in wheat

In this study, a total of 93 *UBC* genes were identified in wheat, which were designated as *TaUBC1* to *TaUBC93* based on the distribution of *UBC* gene members on chromosomes (Table S1). The results showed that the length of the coding regions of all members ranged from 318 bp (*TaUBC30*) to 3234 bp (*TaUBC39*), and the theoretical molecular weights (MW) of the coding proteins ranged from 12.05 kDa (TaUBC30) to 118.59 kDa (TaUBC39), with isoelectric points (pI) varying from 4.19 (TaUBC47) to 9.77 (TaUBC77). Moreover, prediction of subcellular localization showed that TaUBC proteins were mainly found in the nucleus and cytoplasm, accounting for 51.6% (48/93) and 20.4% (19/93), respectively. Some TaUBC proteins were associated with chloroplasts, mitochondria, cytomembranes, or the endoplasmic reticulum (Table S1).

### Phylogenetic and structural analysis of *UBC* genes

**Fig. 1:**
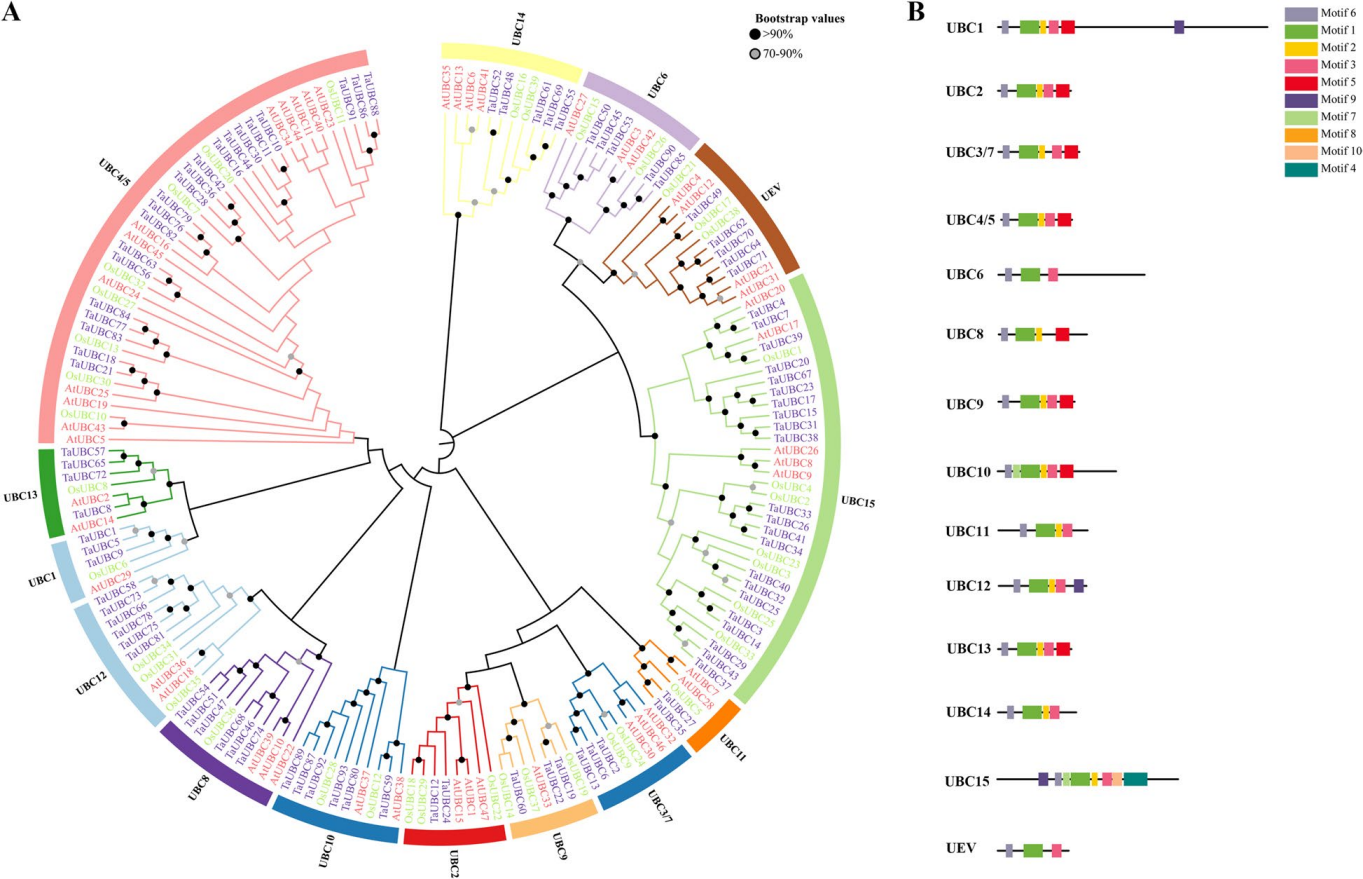
Phylogenetic relationships and conserved motifs of UBC proteins. **A** Phylogenetic analysis of *Arabidopsis* (47), rice (42), and wheat (93) generated using MEGA 11 software with default parameter settings. The different colored blocks indicate different subclasses of the UBC family. The TaUBC family was clustered into 14 clades, and each clade contained 3, 2, 3, 21, 5, 6, 3, 6, 2, 6, 4, 5, 22 and 5 TaUBCs, respectively. The name of each branch was indicated next to the corresponding branch. **B** Conserved motifs of UBC proteins. Distribution of the 10 conserved motifs in the TaUBC proteins after analysis by the MEME tool. The different colored boxes represent different motifs and their position in each protein sequence of TaUBC

To further evaluate the evolutionary relationships of UBC proteins in different plant species, the full-length amino acid sequences of 47 AtUBC, 39 OsUBC, and 93 TaUBC were selected for multiple sequence alignment and construction of a Neighbour-Joining phylogenetic tree (Fig. 1A). The phylogenetic tree results showed that these UBC proteins could be divided into 13 E2 groups and one UEV-independent group based on high bootstrap analysis, including UBC1, UBC2, UBC3/7, UBC4/5, UBC6, UBC8, UBC9, UBC10, UBC11, UBC12, UBC13, UBC14, UBC15, and UEV, which is essentially consistent with previous reports [15]. As shown in Fig. 1A, the UBC4/5 subfamily has the most members and contains twelve AtUBC, eight OsUBC, and twenty-one TaUBC. The UBC1 and UBC11 subfamilies have the fewest members, each containing only five group members.

To further investigate the structural diversity of TaUBC proteins, the motif compositions of 93 TaUBC proteins were identified and visualized using MEME software [37]. A total of 10 conserved motifs were identified, referred to here as motifs 1 to 10 (Fig. 1B, Fig. S1B). Motifs 1, 2, 3, 5, and 6 were found in most sequences, suggesting the functional similarity and conserved position of the genes in wheat (Fig. S2). Motif 1 was most frequently conserved in all E2 members (Fig. 1B, Fig. S1B). In addition, motifs 4, 7, 8, 9, and 10 were selectively present in certain subgroups of the TaUBC family, such as motif 9, which is present in only three proteins of the UBC1 subfamily, with an additional or specific function and has been found in other plant species [5, 16].

Structural analysis of *TaUBC* genes showed that the number of introns in the coding sequences of 93 *TaUBC* genes varied from 1 (*TaUBC56/63/77/83/84*) to 8 (*TaUBC14/85/90*), and most members within the same subfamily had the same exon/intron structure (Fig. S1). For example, *TaUBC86/88/91* from *UBC4/5* all contained three introns and four exons that were about 3500 bp in length, similarly *TaUBC22/19/60* from *UBC9* each contained four introns and five exons that were nearly 3000 bp long, while *TaUBC2/6/13* from *UBC3/7* each contained 5 introns and 6 exons, indicating their evolutionarily conserved features with possibly similar functions.

### Chromosome localization, gene duplication and collinearity analysis of *UBC* gene family

Based on the *Ensembel* Plants database, the 93 *TaUBC* genes were unevenly distributed across 21 chromosomes of the wheat genome (Fig. S3B).Among these, chromosome 5B had the greatest number of *TaUBC* genes (8 out of 93), while there were only two *TaUBC* genes on each of chromosomes 2B and 7B. Gene duplication events such as segmental duplication and tandem duplication during polyploidization of wheat lead to gene expansion [38].

**Fig. 2:**
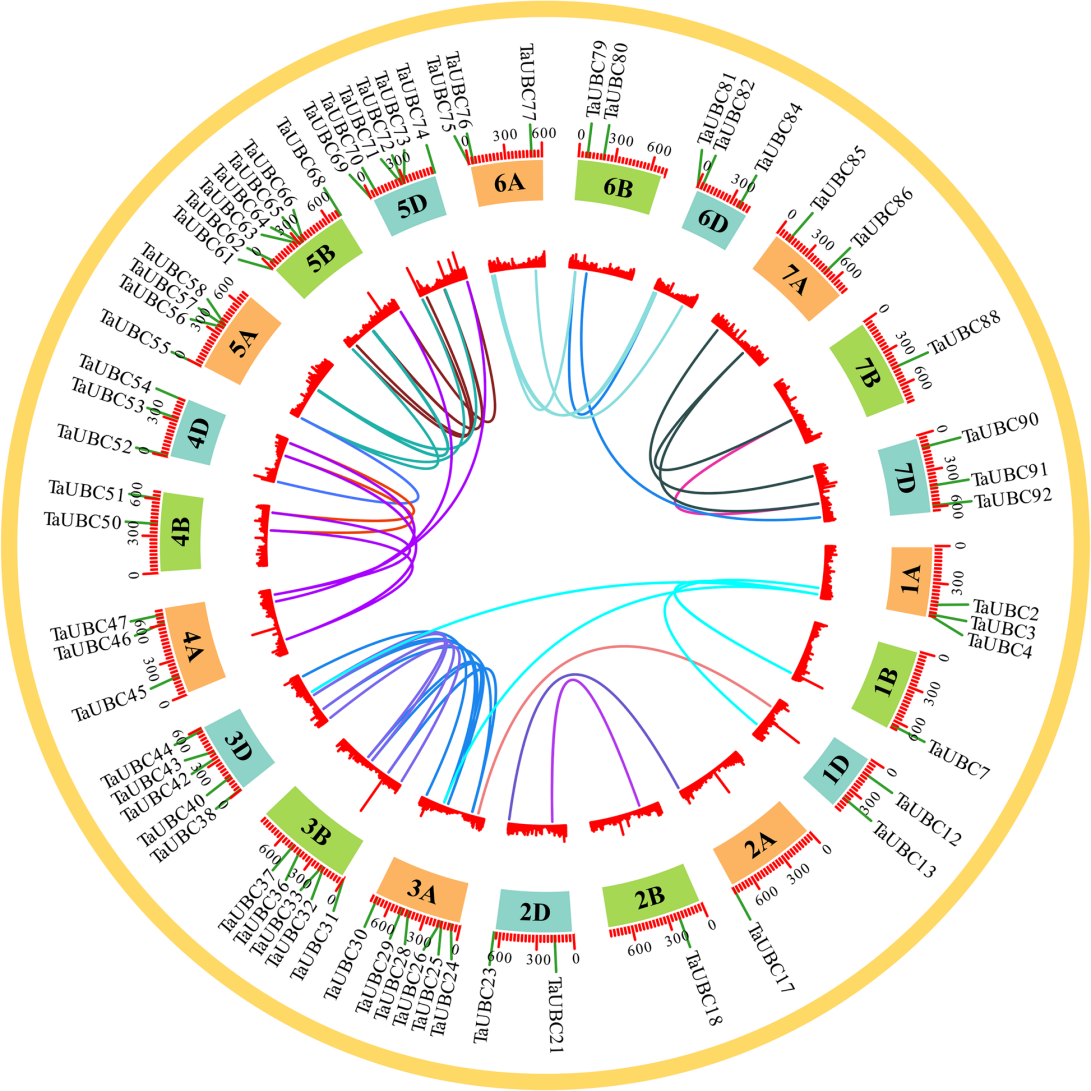
Distribution of *TaUBCs* segment duplication gene pairs on wheat chromosomes. Duplicated gene pairs are connected by lines with the corresponding color. The different colored blocks indicate different chromosome. The chromosome number is indicated on the inner side of each chromosome. The heatmap in the inner circle represents the gene density on the chromosome

To investigate the contribution of *TaUBC* family duplication events, we analyzed the occurrence of tandem duplication and large-scale segmental duplication during evolution. Synteny results showed that 69.9% (65/93) of *TaUBC* genes had duplication events, all of which experienced segmental duplication but no tandem duplication (Fig. 2, Table S2). In addition, the duplicated gene pairs had the most members in the *UBC4/5* (13 pairs) and *UBC15* (12 pairs) subfamilies, and no duplicated gene pairs were found in the *UBC1* and *UBC9* subfamilies (Table S2), indicating that segmental duplication is the main driver of gene expansion in the *TaUBC* family.

Calculation of Ka/Ks values of the duplicated gene pair for all *TaUBC* repeat genes (Table S2) suggests that *TaUBC* are driven by selection pressure and the family members are more conserved during evolution. In addition, synteny analysis of *UBC* genes in wheat, rice, and Arabidopsis revealed that 51 *TaUBC* genes are homologous to *OsUBC* genes in rice and *AtUBC* genes in Arabidopsis, indicating that they may have similar biological functions (Fig. S3A).

### Expression analysis of *TaUBC* genes in various organs at different developmental stages

**Fig. 3:**
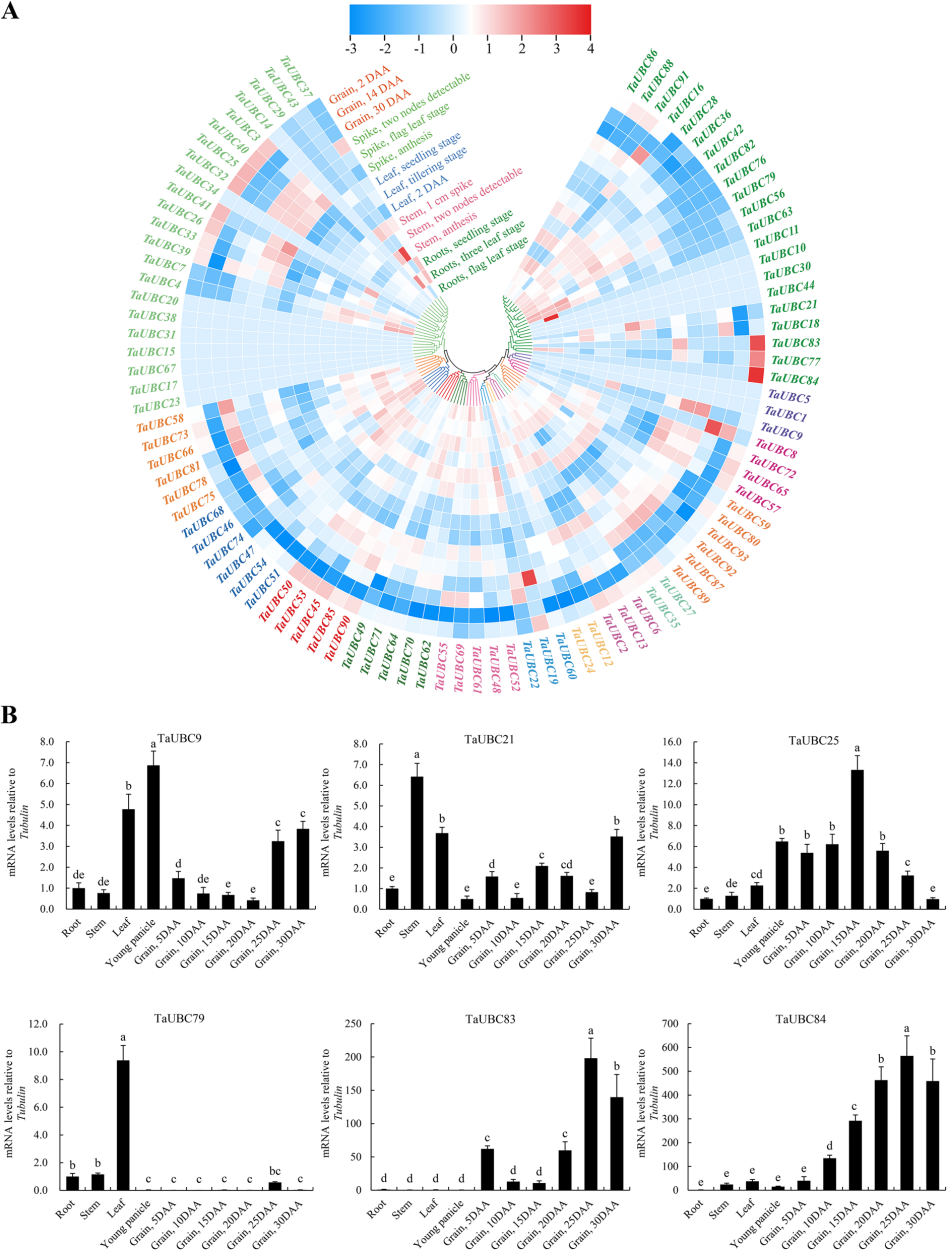
Expression analysis of the *TaUBC* genes. **A** Heatmap of *TaUBC* genes expression in different growth stages and different tissues of Chinese Spring. The heatmap shows the phylogenetic clustering of 93 *TaUBC* genes. Color scale: Blue represents low expression and red indicates high expression levels. **B** Expression profiles of *TaUBCs* over developmental period of wheat. *Tubulin* (TraesCS1A01G350200.1) was used as a reference gene. The Y-axis and X-axis indicates the relative expression levels and the different tissues, respectively. Means and standard deviations (SDs) were calculated from three biological and three technical replicates. The error bars indicate the standard deviation

The expression level of *TaUBC* genes in roots, stems, leaves, spikes, and grains at different developmental stages were analyzed using wheat RNA-Seq data from the expVIP expression database. The expression patterns of *TaUBC* genes showed that they were generally highly expressed in spikes and grains (Fig. 3A). *TaUBC* genes in the same subgroup generally showed a similar expression pattern. *TaUBC3/40/25/32/26* in the *UBC15* subfamily and *TaUBC83/77/84* in the *UBC4/5* subfamily had higher expression levels in spikes and young grains (Grain, 2DAA) compared with other organs. Moreover, the findings indicated that the expression levels of the majority of duplicated gene pairs were consistent across various tissues and developmental stages, such as *TaUBC4/7* and *TaUBC86/88*, which were widely expressed in diverse organs and developmental phases.

In the subsequent, real-time quantitative PCR (qRT-PCR) was used to assess the expression levels of six *TaUBC* genes. Since *TaUBC25*, *TaUBC83*, and *TaUBC84* are highly expressed in grains, *TaUBC9*, *TaUBC11*, and *TaUBC21* are highly expressed in young panicles, stems, and leaves, respectively, and therefore they were selected for qRT-PCR detection. The results showed the predominant expression of *TaUBC25/83/84* in the grain (Fig. 3B). Conversely, *TaUBC9*, *TaUBC79*, and *TaUBC21* exhibited primary expression in young panicles, leaves and stems, respectively. The qRT-PCR results of generally consistent with the *in-silico* expression analysis data obtained from the expVIP database Among them, the heatmap shows that *TaUBC25* is mainly highly expressed in grains (2DAA) and panicles (Fig. 3A), while qRT-PCR data shows that *TaUBC25* is constitutive and highly expressed in grains and panicles (Fig. 3B). The differences between these two result may be caused by the differences in calculation methods.

### Regulation of *TaUBC* genes expression in response to abiotic stress

Analysis of the *cis*-acting elements of the promoters of *TaUBC* genes identified several *cis*-acting elements related to the response to abiotic stress (Fig. S5). Therefore, the expression patterns of *TaUBC* genes under different abiotic stress treatments, including low temperature (4 ℃), salt (200 mM NaCl), and drought (20% PEG6000), were analyzed using transcriptome data from the expVIP database (Fig. S4). The results showed that the expression levels of *TaUBC37/43/29* in *UBC15*, *TaUBC60* in *UBC9*, and *TaUBC12* in *UBC2* was down-regulated upon low-temperature treatment, whereas the expression of all genes in *UBC4/5* except *TaUBC18* was up-regulated. The expression levels of *TaUBC* gene family members were generally down-regulated after NaCl treatment. On the other hand, the expression levels of *TaUBC3/7/14/25/32/40* in *UBC15* and *TaUBC55/69* in *UBC14* were up-regulated after NaCl treatment, while the expression of *TaUBC17/20* was significantly increased after 48 h of NaCl treatment. Moreover, while the PEG6000 treatment affected the expression of all wheat *TaUBC* genes, each gene exhibited distinct responses. Specifically, seven genes (*TaUBC85/90/60//13/6/66*) displayed substantial up-regulation, reaching peak expression levels after 24 h. In contrast, *TaUBC37* was the sole gene exhibiting down-regulation.

**Fig. 4:**
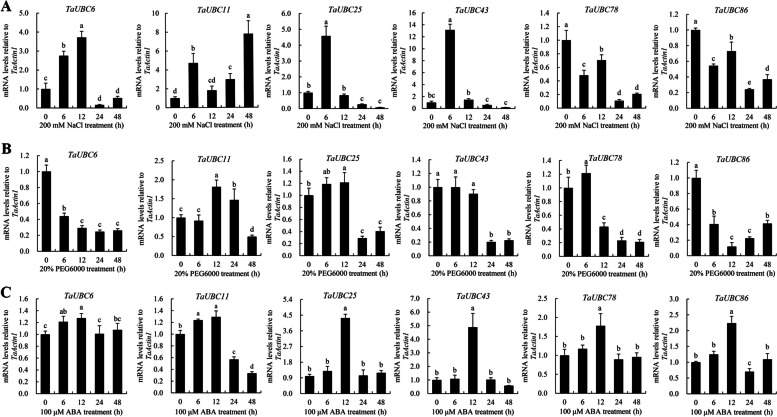
qRT-PCR analysis of selected *TaUBCs* under 200 mM NaCl, 20% PEG6000, and 100 uM ABA stress. **A** Relative expression patterns of *TaUBCs* in leaves after 200 mM NaCl treatment. **B** Relative expression patterns of *TaUBCs* in leaves after 20% PEG6000 treatment. **C** Relative expression patterns of *TaUBCs* in leaves after treatment with 100 uM ABA. Relative expression values in the control sample (CK 0 h) were normalized to 1. *TaActin1* was used as a reference gene. Each bar value is the average value ± standard deviation based on three biological replicates. The different letters denote a significant difference between means (*P* < 0.05)

To further investigate whether *TaUBC* genes in wheat respond to abiotic stress, we used qRT-PCR to detect the expression of six selected *TaUBC* genes, including *TaUBC6*, *TaUBC11*, *TaUBC25*, *TaUBC43*, *TaUBC78*, and *TaUBC86*, in 14-day-old seedlings after treatment with various stress conditions such as salt (200 mM NaCl), 20% PEG6000, and ABA (100 μΜ ABA). The qRT-PCR results showed that the expression of *TaUBC78* and *TaUBC86* was slightly suppressed, whereas the expression of *TaUBC6*, *TaUBC11*, *TaUBC25*, and *TaUBC43* was significantly increased under 200 mM NaCl treatment. In particular, *TaUBC25* and *TaUBC43* was rapidly up-regulated after 6 h under treatment and peaked (Fig. 4A, Fig. S6A). The expression levels of *TaUBC6*, *TaUBC43* and *TaUBC86* were significantly reduced, whereas *TaUBC11*, *TaUBC25* and *TaUBC78* were induced under 20% PEG6000 treatment (Fig. 4B, Fig. S6B). The qRT-PCR results generally consistent with the in-silico expression analysis data obtained from the expVIP database. Among them, the heatmap shows that *TaUBC11* is mainly highly expressed in NaCl treatment for 12 h, while qRT-PCR data shows that *TaUBC11* is highly expressed in NaCl treatment for 24 h. The differences between them these two results may be caused by the differences in calculation methods and wheat varieties. The inconsistency between the qRT-PCR data of some genes and the *in-silico* analysis may be due to differences in their algorithms. The ABA pathway is typically involved in signal transduction under various abiotic stresses. Under ABA treatment, all six genes showed significantly up-regulated expression, and were induced after 6 h treatment and reached peak at 12 h (Fig. 4C, Fig. S6C). Therefore, the RNA-Seq data and qRT-PCR results revealed that a part of *TaUBC* genes may be involved in the regulation of diverse abiotic stress.

### Analysis of *TaUBC25* allelic variation in wheat

Because *TaUBC25* was significantly highly expressed in the wheat grain thus we further analyzed whether the function of *TaUBC25* could be associated with the control of seed traits. For this purpose, the single nucleotide polymorphisms (SNPs) between the sequences of *TaUBC25* were analyzed in 681 wheat cultivars whose genomic sequences could be obtained from the Wheat Union database (http://wheat.cau.edu.cn/WheatUnion/). This led to the identification of two types of *TaUBC25* gene haplotypes in different wheat cultivars (Table S5).

**Fig. 5:**
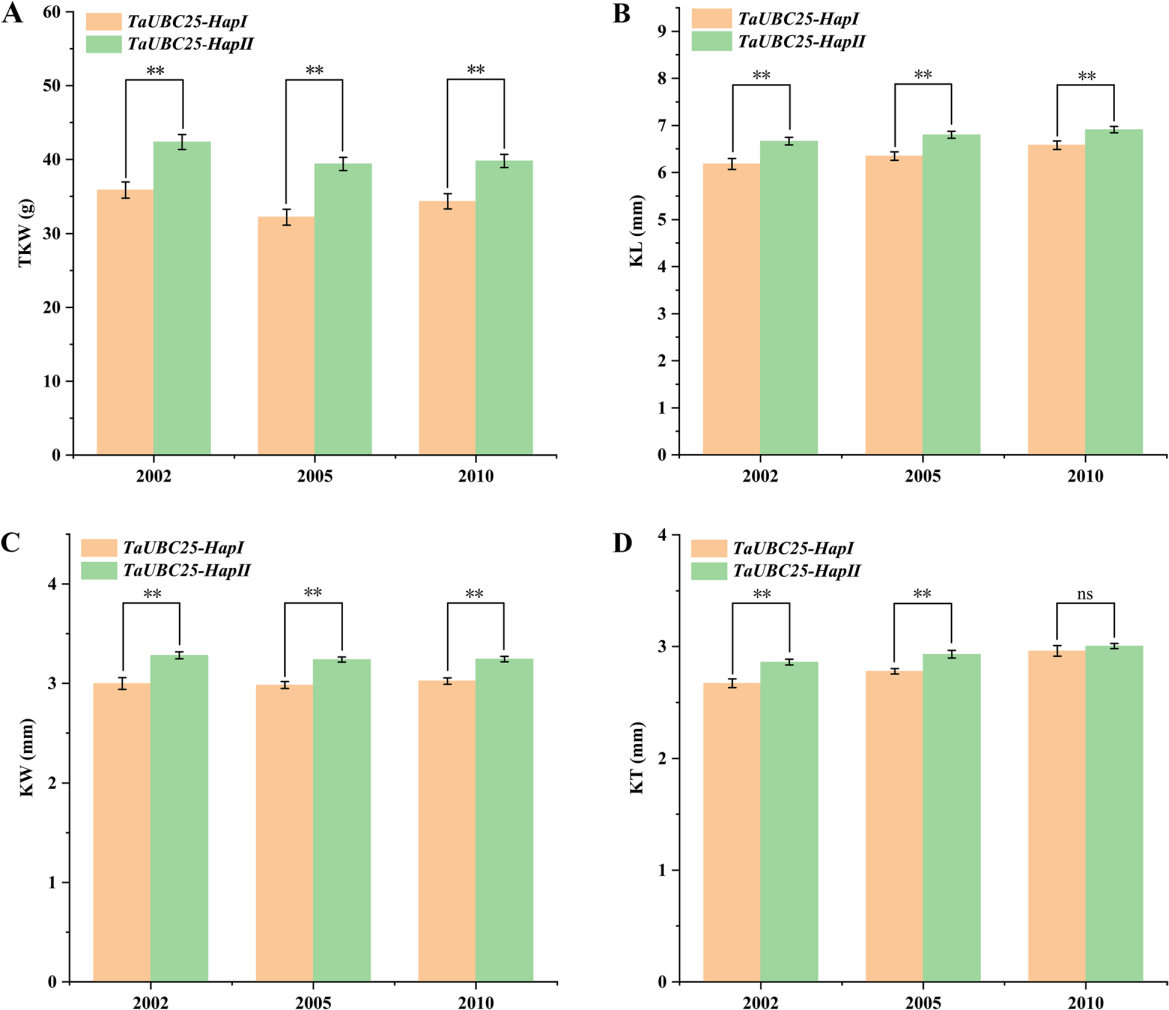
Association analysis of TKW, KL, KW and KT between two haplotypes of *TaUBC25* gene in three environments. **A** The mean thousand grain weight of *TaUBC25* haplotypes in the modern cultivars. **B** The mean kernel length of *TaUBC25* haplotypes in the modern cultivars. **C** The mean kernel width of *TaUBC25* haplotypes in the modern cultivars. **D** The mean kernel thickness of *TaUBC25* haplotypes in the modern cultivars. The X-axis represents different environments (Luoyang, 2002; Luoyang, 2005; Shunyi, 2010). The values show the mean ± SE (*n* > 50 grains). Significant statistical analysis was carried out by Student’s *t*-test (*, *P* < 0.05; **, *P* < 0.01)

The grain-related traits of 122 wheat accessions, grown at Luoyang, Henan Province, in 2002 and 2005, and at Shunyi, Beijing, in 2010 were acquired from previous report [39] and used for association analysis. The results showed that TKW, KL, KW, and KT were significantly different between the two haplotypes of *TaUBC25* gene (Fig. 5, Table S6). There were highly significant differences (*P* < 0.01) in TKW, KL, KW, and KT (assessed over 3 years) between the two haplotypes *TaUBC25-Hap I* and *TaUBC25-Hap II*. Consequently, TKW, KL, KW, and KT of *TaUBC25-Hap II* were significantly higher (*P* < 0.01) than those of *TaUBC25-Hap I* under three environmental conditions.

**Fig. 6:**
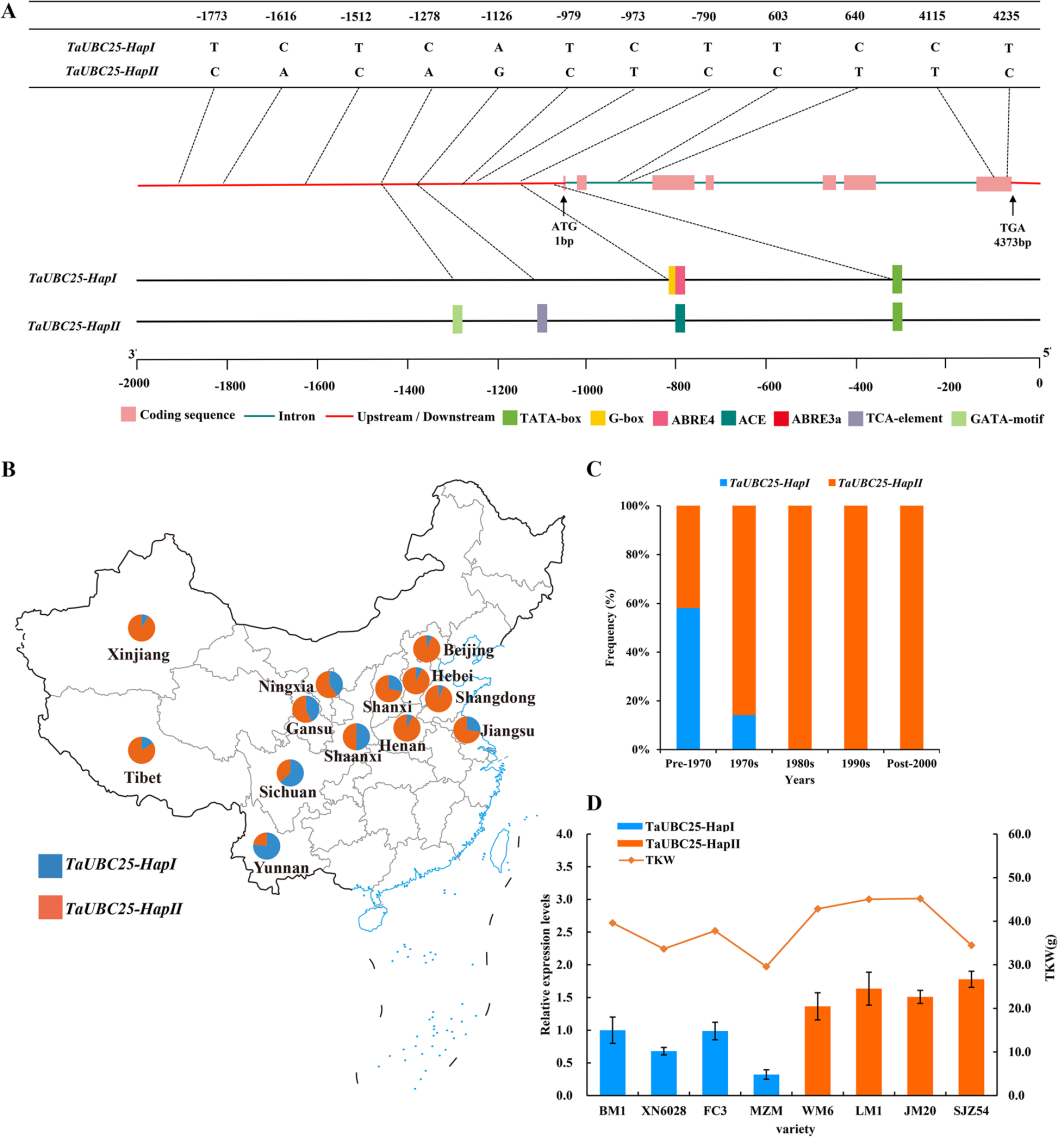
Analysis of the different* cis*-element existed in the promoter region, spatial and temporal distribution, and expression levels of two *TaUBC25* haplotypes. **A** The distribution of main *cis*-regulatory elements contained SNPs site in the promoter region of the two haplotypes of *TaUBC25* gene. **B** Geographic distribution of varieties with *TaUBC25* haplotypes in China. The map was downloaded using the Standard Map Service System (http://bzdt.ch.mnr.gov.cn/). **C** Frequencies of *TaUBC25* allelic variation in Chinese wheat breeding programs in different decades. **D** The expression levels of *TaUBC25* and TKW carrying *TaUBC25-Hap I* or *TaUBC25-Hap II* haplotypes

In the next step, analyzing the promoter regions of the two haplotypes of the *TaUBC25* gene revealed eight SNP (single-nucleotide polymorphisms) variants at positions -790 (T/C), -973 (C/T), -979 (T/C), -1126 (A/G), -1278 bp (C/A), -1512 (T/C), -1616 (C/A), and -1773 (T/C), respectively, upstream of the start codon ATG (Fig. 6A). The prediction of *cis*-elements indicated that these eight SNPs might contribute in the formation of several transcription factor binding sites, including the GATA motif. This implies a potential close link between these SNPs and the regulation of seed development in wheat.

Wheat breeding selection leaves a strong footprint in the genome. To better understand whether *TaUBC25-Hap II* was positively selected in wheat breeding, the geographical distribution of two haplotypes of the *TaUBC25* gene was examined using 312 genotypes from 13 provinces in China (Fig. 6B, Table S7). The distribution frequencies of *TaUBC25-Hap II* from 13 provinces in China were 93.75% (Beijing), 57.14% (Gansu), 92.31% (Hebei), 92.86% (Henan), 72.73% (Jiangsu), 60.00% (Ningxia), 93. 94% (Shandong), 71.43% (Shanxi), 50.00% (Shaanxi), 36.84% (Sichuan), 84.40% (Tibet), 91.67% (Xinjiang), and 23.08% (Yunnan), indicating that *TaUBC25-Hap II* is a favorable haplotype. We also determined the frequencies of *TaUBC25-Hap II* in the historical population of 99 accessions over 10-year intervals (pre-1970, 1970s, 1980s, 1990s, and post-2000) of evaluation. As a result, the frequency of the favorable haplotype *TaUBC25-Hap II* increased sharply from 41.67% to100% in the cultivars released from pre-1970 to 1980s, and subsequently remained stable in the decades from 1990s to post-2000, while the frequencies of the haplotype *TaUBC25-Hap I* showed the opposite trend (Fig. 6C; Table S8). The results suggest that the favorable *TaUBC25-Hap II* haplotype is subject to positive selection in wheat breeding, particularly in China.

Finally, to further analyze the effect of favorable allelic variation in regulating wheat grain development, eight cultivars with *TaUBC25-Hap I* or *TaUBC25-Hap II* from 122 cultivars (mentioned above) were used to test the levels of *TaUBC25* gene expression in spike at the grain filling stage. The qRT-PCR results showed that genotypes with *TaUBC25-Hap II* had relatively higher expression levels than those with *TaUBC25-Hap I* (Fig. 6D), suggesting that *TaUBC25-Hap II* may contribute to increasing grain yield in this wheat material.

## Discussions

The ubiquitin proteasome system (UPS) is mediated by three enzymatic cascades, which include ubiquitin-activating enzymes, ubiquitin-binding enzymes, and ubiquitin-ligases [9]. Ubiquitin-binding enzymes are important components of the UPS and play a crucial role in plant development and growth. The function of E2 ubiquitin-binding enzymes in higher plants has been relatively insufficient compared to the extensive studies on E3 ubiquitin ligases. Therefore, a systematic analysis of members of the E2 ubiquitin-binding enzyme family is crucial to elucidate their role in plant development and in responding to adverse changes in the external environment. In this study, we identified a total of 93 wheat *UBC* genes. The phylogenetic tree results showed that these 93 UBC proteins could be divided into 13 E2 groups and 1 UEV-independent group, which is consistent with previous reports [15].

We found that 69.9% (65/93) of wheat *TaUBC* genes had duplication events (Fig. 2). A total of 51 duplicated gene pairs of *TaUBC* family genes were subjected to fragmentary duplication with Ka/Ks values less than 1, indicating that *TaUBC* genes are conserved in the wheat genome by fragmentary duplication, which overall contributes to the expansion of the *TaUBC* gene family in wheat. Since wheat has undergone two rounds of polyploidization, [40], the expansion of *TaUBC* family members in wheat should be due to gene duplication. Gene duplications are an important mechanism for gene family evolution. This event has occurred repeatedly in angiosperms and has resulted in gene combination or amplification, which in turn has resulted in gene family expansion [41]. Gene duplications also play an important role in adaptation to environmental stimuli, with family members being better maintained over evolutionary time following this event, which was triggered by a purifying selection pressure [42–46]. Gene duplications have been also reported to occur among UBC family members in Arabidopsis, rice [14], maize [15], and potato [18].

Motif analysis revealed that each subgroup of TaUBC proteins contained a conserved structural domain of UBC [47, 48]. According to the phylogenetic analysis and amino acid sequence similarity (Fig. 1), the 93 members of the TaUBC proteins family can be classified into 14 groups, including 13 E2 groups and 1 UEV group, which is generally consistent with the phylogenetic analysis of the UBC family in Arabidopsis [20], tomato [29] and rice in previous studies [49]. The variation in numbers among the different *TaUBC* subfamilies suggests that the *TaUBC* family has undergone genealogy-specific expansion and functional divergence during evolution. Phylogenetic analysis of the *TaUBC* genes also revealed that UBC9 and UBC15 are amplified in monocots but not in dicot Arabidopsis. One member of UBC9 was identified in Arabidopsis, while three members were identified in both wheat and rice. For UBC15, five members were identified in Arabidopsis, while 22 and seven members were identified in wheat and rice, respectively. This suggests that the UBC-encoding genes of these subclasses evolved species-specifically from common precursor genes that existed before the diversification of monocot and dicot gene lineages.

The results of our study showed that a large number of elements related to plant growth and development, hormone response, and stress response were present in the promoter region of *TaUBC* genes (Fig. S5). We also observed that the different subgroups contained different numbers and types of *cis*-elements, suggesting a possible functional differentiation of *TaUBC* genes [50, 51]. According to the expression patterns of *TaUBC* genes, we found that the same subgroup and most duplicated gene pairs generally have shown similar expression patterns in different tissues and developmental stages (Fig. 3A) and under abiotic stress conditions (Fig. S4). This finding was subsequently confirmed by qRT-PCR analysis, in which we identified tissue-specific expression of *TaUBC* genes (*TaUBC9/79/25/21/83/84*) predominantly in young panicles, leaves, stems, and grains (Fig. 3B). The analyses of the temporal and spatial expression patterns of *TaUBC* genes provided valuable insights into their potential functions in wheat. Other studies have also reported that the expression of *E2* genes in many plant species is regulated by tissues and developmental stages as well as environmental conditions [22, 52, 53]. Overexpression of *SiUBC32* can significantly affect the flowering time of foxtail millet (*Setaria italica*), thereby impacting seed development. This indicates that this gene plays a key role in regulating grain yield [54]. Heterologous expression of the soybean (*Glycine max*) *GmUBC9* gene significantly increased the expression level of flowering-related genes in Arabidopsis, exhibiting a late flowering phenotype [8]. *OsUBC26* is involved in the proteasomal degradation of the *M. oryzae* effector protein AvrPiz-t [55], *OsUBC45* is localized in the endoplasmic reticulum of cells and is involved in regulating plant immunity as a component of ERAD [56]. In addition, the SMG3 encoding OsUBC45 controls rice grain size and weight [56, 57], and OsUBC45 can interact with OsGSK3 and OsPIP2;1 to mediate their degradation in rice to promote yield [58]. In wheat, an E2 ubiquitin-conjugating enzyme *UBC* gene has been successfully cloned and named *TaUBC*. It has been found that this gene is constitutively expressed in roots, stems, and leaves of wheat. In vitro ubiquitination activity assay confirmed that *TaUBC* possesses ubiquitin-conjugating enzyme activity, which confirms its involvement in wheat plant development and yield regulation [59].

In this study, several *TaUBC* genes, such as *TaUBC6/11/25/43/78/86*, were found to be significantly up-regulated after 12 h of ABA treatment (Fig. 4). These results correlate with the promoter region containing a large number of ABA-responsive elements, suggesting that the function of these *TaUBC* genes responding to abiotic stress may lie in an ABA-dependent signaling pathway. Abiotic stresses such as drought, cold, and salt stress can cause cell dehydration, which can severely affect wheat growth and development, resulting in more or less severe wheat yield losses [49, 60, 61]. Meanwhile, some studies have identified an important role of certain E3 ligases and other components of the UPS in plant stress responses [6, 7, 46]. For example, many genes encoding E2 enzymes in the rice and potato genomes are induced by salt and cold stress and abscisic acid (ABA) [16, 49], showing that *E2* genes play an important regulatory role in abiotic stress responses. The *OgUBC1* gene is involved in cellular responses to biotic and abiotic stress in wild rice [52]. *AtUBC32* plays a role in brassinosteroid (BR)-mediated salt tolerance in plants [22]. In soybean, the expression of *GmUBC2* was up-regulated in drought and salt stress [53]. In addition, we found that elements related to environmental stress were abundant (Fig. S5), including STRE, MYB, ARE, GC-Motif, etc. The most abundant and widespread of the 13 hormone response elements were ABREs, which respond to ABA [62, 63]. ABREs are the most important *cis*-regulatory elements that play an important role in plant resistance to biotic or abiotic adversities [64].

Wheat yield is mainly determined by thousand kernel weight (TKW), grain number per spike (GNPS) and spikelet number per spike (GNS),which is a complex quantitative trait [45]. In recent years, with the development of molecular biology and genetics, many genes regulating seed development are involved in protein degradation by UPS, suggesting that UPS may play a key role in wheat seed development. In previous studies, genes related to controlling grain weight such as *GW2* [65, 66], *DA1* [67], *GW7* [68] and *Sus2* [69], have been studied in detail, and their superior haplotypes have been positively selected in Chinese wheat breeding. Association analysis of the natural genetic variations of four agronomic traits in three environments in the present study showed that the *TaUBC25-Hap II* had a significantly higher TGW than the *TaUBC25-Hap I* haplotype (Fig. 5). Our analysis of the geographical distribution of 312 wheat accessions in China suggests that genetic variation in *TaUBC25-Hap II* was artificially and strongly selected for during wheat domestication breeding (Fig. 6B). Moreover, the frequency of the *TaUBC25-Hap II* in wheat breeding in China increased rapidly in the 1970s and remained at a stable high level after the 1980s (Fig. 6C). This period coincides with the green revolution period in wheat [70], suggesting that the favorable *TaUBC25-Hap II* of *TaUBC25* may have been strongly selected by breeders during this time in China. To further analyze the effect of favorable allelic variation on the regulation of wheat grain development, qRT-PCR analysis was performed on eight cultivars with *TaUBC25-Hap I* or *TaUBC25-Hap II*. The results showed that the genotypes with the *TaUBC25-Hap II* gene had relatively higher expression levels than those with the *TaUBC25-Hap I* gene, and their TKWs also showed consistent phenotypes. It was experimentally confirmed that the *TaUBC25* gene is related to wheat seed development and the *TaUBC25-Hap II* haplotype is an excellent haplotype for wheat yield improvement that can be used in future wheat breeding programs.

## Conclusion

In summary, a total of 93 *TaUBC* genes were identified and divided into 13 E2 groups and a separate UEV group from a wheat genome. The results of RNA-Seq and qRT-PCR suggested that some *TaUBC* genes were specifically expressed in certain tissues/organs and that most *TaUBC* genes responded to NaCl, PEG6000, and ABA treatment with different levels of expression, which results will be valuable for further studies on the biological role of *TaUBC* genes in grain development and response to stress conditions and may shed light on the improvement of the genetic breeding of wheat. Based on the SNPs detected in the *TaUBC* sequence, two haplotypes, *TaUBC25-Hap I* and *TaUBC25-Hap II*, were identified among wheat cultivars. Association analysis showed that allelic variation *TaUBC25-Hap II* displayed higher TKW, KL, KW and KT compared with *TaUBC25-Hap I* allelic variation, and and *TaUBC25-Hap II* haplotype is widely planted in the primary wheat-producing regions of China. qRT-PCR showed the expression of the *TaUBC25* gene in eight haplotypes, exhibiting a significantly higher expression in *TaUBC25-Hap II* than in *TaUBC25-Hap I*. Our results provide potential value for wheat breeding. The above results provide a theoretical basis for elucidating the evolutionary relationships of the *TaUBC* gene family and also provide insights into the molecular regulation of the *TaUBC25* gene.

## Materials and methods

### Plant materials, growth conditions, and stress treatments

Wheat variety JinMai47 which has diverse abiotic tolerance and has been widely planted in China [71, 72] was used as experimental material for this study. Grains were sterilized with 10% sodium hypochlorite, spread on sterilized Petri dishes, and cultured in 1/2 Hoagland nutrient solution under photoperiod conditions of 16/8 h (light/dark, 25/22 °C). The 1/2 Hoagland nutrient solution was replaced every five days to ensure a constant supply of nutrients. For the stress treatment experiments, the 1/2 Hoagland nutrient solution was replaced with: (I) 1/2 Hoagland nutrient solution added with 200 mM NaCl, (II) 1/2 Hoagland nutrient solution added with 20% PEG6000, (III) 1/2 Hoagland nutrient solution added with 100 uM ABA, and (IV) 1/2 Hoagland nutrient solution alone, as the control treatment, when wheat seedlings were 14-day-old. Then the first leaves of the three wheat seedlings were collected after 0, 6, 12, 24, and 48 h after the stress treatments in each biological replicate. Three biological replicates were performed for qRT-PCR analysis. For tissue-specific gene expression analysis, untreated wheat seedlings were transferred in pots for culturing. Then roots, stems, leaves and young panicles at anthesis, and developing grain (5, 10, 15, 20, 25, and 30DAA) stage were collected from three wheat plants, respectively. Eight wheat varieties with *TaUBC25-Hap I* or *TaUBC25-Hap II* haplotypes were selected from 122 wheat accessions and spikelets were collected at the grain filling stage to test the expression of the two haplotypes. Three biological replicates were performed to analyze the data. All samples were stored in liquid nitrogen immediately after collection, and thereafter stored in an ultra-low temperature freezer.

### Identification of the UBC gene family in wheat

To identify UBC family members in wheat, UBC protein sequences in rice and Arabidopsis were retrieved from the Rice Genome Annotation Project database (http://rice.plantbiology.msu.edu/) and the Arabidopsis Information Resource (TAIR) database (http://www.Arabidopsis.org), respectively [73]. In addition, whole genome data, CDS sequences, protein sequences, and annotation databases of *Triticum aestivum* (IWGSCRefSeq_v1.0), were downloaded from the Ensembl Plants database (http://plants.ensembl.org) [73]. In addition, the hidden Markov model (HMM) profile for the UBC domain (PF00179) was downloaded from Pfam (http://pfam.xfam.org/family/PF00179) [74], and then applied to the whole genome protein sequence of wheat using the HMMER 3.0 [75] software program (threshold E < 1e-5) to obtain *TaUBC* candidate genes. The wheat UBC proteins containing the structural domain of E2 ubiquitin-binding enzymes were confirmed using the NCBI Conserved Domain Database (NCBI-CDD) (https://www.ncbi.nlm.nih.gov/Structure/bwrpsb/bwrpsb.cgi), Inter Pro (http://www.ebi.ac.uk/interpro/scan.html), and SMART (http://smart.embl-heidelberg.de/) [76–78].

The proteins without UBC domains were removed. Based on the phylogenetic clustering of UBCs in Arabidopsis and rice, the identified wheat *UBC* genes were renamed as *TaUBCs* described in a previous report [79]. The ExPASy server (https://web.expasy.org/compute_pi/) was used to calculate the molecular weight (MW), theoretical isoelectric point (pI), grand average hydropathicity (GRAVY), and amino acid sequence length of the proteins. Gpos-mPLoc (http://www.csbio.sjtu.edu.cn/bioinf/Gpos-multi/) was used to predict the subcellular localization of wheat UBC proteins [80].

### Phylogenetic, gene structure, and motif analysis of *UBC* genes

Phylogenetic tree construction allows further investigation of gene conservation during evolution. Multiple sequence alignments of wheat, Arabidopsis, and rice UBC proteins were performed using ClustalW and standard parameters. The phylogenetic tree was constructed with the 179 UBC full-length amino acid sequences using MEGA 11.0, and the neighbor-joining (NJ) method was used with the following parameters: Poisson correction, pairwise deletion, and bootstrap (1000 repeats; random seed) [81, 82]. The phylogenetic tree was visualized using the online software ChiPlot (https://www.chiplot.online/tvbot.html) [83]. The phylogeny analysis is performed on full-length amino acid sequence. Wheat genome annotation (GFF3) files were obtained from the International Wheat Genome Sequencing Consortium (IWGSC) (https://urgi.versailles.inra.fr/download/iwgsc/IWGSC_RefSeq_Annotations/v1.0/). By comparing the genomic and corresponding cDNA sequences of wheat *TaUBC* genes, the schematic structure of *TaUBC* genes was determined using Gene Structure Display Server (GSDS 2.0, http://gsds.gao-lab.org/index.php). The protein-conserved motifs were identified using the Multiple Expectation Maximization for Motif Elicitation (MEME) online tool (http://meme.nbcr.net/meme) [37] with the motif number set to 10 and visualized using TBtools software [84, 85].

### Chromosomal location, gene duplication, and collinearity analysis

The chromosomal positions of wheat *UBC* genes were obtained from the Ensembl Plants database (http://ftp.ebi.ac.uk/ensemblgenomes/pub/release-51/plants/gff3/triticum_aestivum/) and mapped to chromosomes using TBtools software [84]. Based on the wheat genome annotation, covariance analysis was performed using the One Step MCScanX module [86, 87] in TBtools software [81]. The Simple Ka/Ks Calculator module was followed to calculate the rate of synonymous substitutions (Ks) and nonsynonymous substitutions (Ka) for duplicate gene pairs. If Ka/Ks > 1, positive selection; Ka/Ks = 1, neutral selection; Ka/Ks < 1, purifying selection [85].

### Analysis of the *cis*‑regulatory elements in the promoter

Wheat whole-genome data were downloaded from the *Triticeae* Multi-omics Center (http://202.194.139.32), and the 2.0-kb DNA sequences upstream from the initiation codon of *TaUBCs* were retrieved using TBtools software [84]. The *cis*-regulatory elements in the promoter regions of the *TaUBC* genes were analyzed for type, number, and function using the PlantCARE website (http://bioinformatics.psb.ugent.be/webtools/plantcare/html/) [73]. The distribution of identified *cis*-elements was plotted using GSDS.

### *TaUBC* genes expression profiles

Available RNA-seq data from different tissues/organs and developmental periods, as well as from plants under different abiotic stress treatments in Chinese spring, were retrieved from the expVIP expression database (http://www.wheat-expression.com) [88, 89] and used to verify the expression patterns of *TaUBC* genes family members. Among them, the material used in the expVIP expression database is Chinese spring wheat plants, which grown in growth pouches supplied with 50% Hoagland’s solution in growth chambers with 12:12 h day: night length at constant 20 °C for 14 days. On the 14th day, plants at the three-leaf stage (Zadok stage 13) were selected for RNA extraction. The different tissues/organs and developmental periods include grain developing at 2 d (grain, 2 DAA), 14 d (grain, 14 DAA), and 30 d (grain, 30 DAA) after flowering; spikes developing at two nodes, flag-picking stage, and flowering stage; leaf development at seedling stage, tiller stage, and 2 d after flowering; stems developing at 1 cm spike length, two nodes, and flowering; and root development at seedling stage, three-leaf stage, and flag picking stage. Abiotic stress included low-temperature stress (wheat, 4 °C), salt stress (NaCl), and simulated drought stress (PEG6000), with data sourced from expVIP database (http://www.wheat-expression.com) [89]. All relative expression values were calculated as transcripts per kilobase of exon model per million mapped reads (TPM) with StringTie using the method described previously [90, 91], and expression values were log2-transformed using TBtools software [84, 85] and displayed as a heat map.

### Total RNA isolation, cDNA synthesis, and quantitative real-time reverse transcriptase-polymerase chain reaction (qRT-PCR)

Wheat tissue/organ samples kept at -80 °C were used for RNA isolation. Total RNA was extracted from all the collected samples using the TIANGEN® RNAprep Pure Plant Kit according to the manufacturer’s protocol. The RNA concentration was measured using a Nanodrop 2000 spectrophotometer (ND2000; Thermo Scientific, Wilmington, NC) and the quality of RNA samples was determined by agarose gel electrophoresis. The first-strand cDNA was synthesized with the TIANGEN® FastKing gDNA Dispelling RT SuperMix.

The qPT-PCR was performed using TIANGEN® FastReal qPCR PreMix (SYBR Green) on the Q2000C fluorescence quantitative PCR system (Long Gene®, China). The wheat *Tubulin* gene [70, 92] (TraesCS1A01G350200.1) was used as a reference gene for wheat tissue development expression analysis, and wheat *TaActin1* [93] (TraesCS1A02G020500.1) and *TaActin3/TaAct* (TraesCS5D02G132200.1) [94] was used as reference genes for wheat expression analysis under different stress treatment. The reaction procedure was as follows: Thermal cycling at 95 °C for 120 s, followed by 39 cycles at 95 °C for 5 s, 58 °C for 10 s, and 72 °C for 15 s. All data were obtained from three biological repeats. The expression levels of wheat root were used as a control for wheat tissue development expression analysis, and the expression levels of wheat untreated leaves was used as a control for wheat stress expression analysis. The 2^−ΔΔCt^ method was used to calculate the relative quantity [95]. To determine statistical for result significance, one-way analysis of variance (ANOVA) was performed in IBM SPSS Statistics 25.0 software (http://www.ibm.com). The primers used are listed in Table S7. The primers efficiency calculation for each primer pair are listed in Table S9. The details on relative expression calculation and statistical methods for result significance are listed in Table S10.

### Haplotype analysis of the *TaUBC25* gene in wheat

The Wheat Union Database (http://wheat.cau.edu.cn/WheatUnion/?language=en) was used to obtain the variant loci of the *TaUBC25* gene in different wheat materials based on the variant information query module using 681 newly sequenced data of hexaploid wheat [32, 34, 36, 96–98]. The differences between the different haplotypes and phenotypic traits were determined using a one-way ANOVA with TASSEL 5.0 software. Phenotypic data of 122 wheat test accessions were obtained from Prof. Xueyong Zhang team at the Institute of Crop Research, Chinese Academy of Agricultural Sciences (CAAS). The phenotypic traits including thousand kernel weight (TKW), kernel length (KL), kernel width (KW) and kernel thickness (KT), were measured on grains produced at Luoyang, Henan Province, in 2002 and 2005, and at Shunyi, Beijing, in 2010 [39]. Wheat genotypes used for spatiotemporal distribution were obtained from the Wheat Union Database (http://wheat.cau.edu.cn/WheatUnion/) [32, 34, 36, 96]. To determine significant statistical differences between the two haplotypes and phenotypic traits, one-way analysis of variance (ANOVA) was performed in IBM SPSS Statistics 25.0 software (http://www.ibm.com).

PlantPAN 3.0 (http://PlantPAN.itps.ncku.edu.tw) was used to analyze the *cis*-regulatory elements in the promoter of the *TaUBC25* gene. The distribution of the identified *cis*-regulatory elements was drawn from GSDS (http://gsds.gao-lab.org/). The Wheat Union database in China was used to analyze the geographical distribution of the two haplotypes of *TaUBC25* in wheat. The map was downloaded from the Standard Map Service System (http://bzdt.ch.mnr.gov.cn/).

### Supplementary Information

**Additional file 1: Figure S1.** Phylogenetic relationships, gene structure and conserved motifs of *TaUBC* genes in wheat. A: The different color blocks indicated different subclasses of the *TaUBC* genes family. B: The distribution of conserved motifs constructed by the MEME. Boxes with different colors represent different conserved motifs (Motif 1–10) C: Structure of *TaUBC* genes, with blue boxes representing UTRs, yellow boxes representing exons, and black lines representing introns. **Figure S2.** Conserved domains of TaUBCs in wheat. **Figure S3.** Collinearity and chromosome localization analysis of *TaUBCs.* A: Collinearity analysis of *UBCs* between wheat and Arabidopsis, rice, which were constructed by TBtools. The gray lines in the background indicate the orthologous genes of wheat and the other two species, while the blue lines highlight the collinear *TaUBC* gene pairs of wheat and the other two species. B: Distribution of *TaUBC* genes on chromosomes. **Figure S4.** Expression heatmap of *TaUBC* genes under different abiotic stress in Chinese spring. The heatmap shows the phylogenetic clustering of 93 *TaUBC* genes. Color scale: Blue represents low expression and red represents high expression levels. **Figure S5.** Analysis of *cis*-elements in the promoter of *TaUBC* genes. **Figure S6.** qRT-PCR analysis of selected *TaUBCs* under 200 mM NaCl, 20% PEG6000, and 100 uM ABA stress. A: Relative expression patterns of *TaUBCs* in leaves after 200 mM NaCl treatment. B: Relative expression patterns of *TaUBCs* in leaves after 20% PEG6000 treatment. C: Relative expression patterns of *TaUBCs* in leaves after treatment with 100 uM ABA. Relative expression values in the control sample (CK 0h) were normalized to 1. *TaAct* was used as a reference gene. Each bar value is the average value ± standard deviation based on three biological replicates. The different letters denote a significant difference between means (*P* < 0.05). **Table S1.** Characteristics of *TaUBC* genes family members. **Table S2.** Information on duplication events of *TaUBC* genes. **Table S3.** Analysis of *cis*-elements in the promoter of *TaUBC* genes. **Table S4.** Primer sequences used in this study. **Table S5.** Variation sites and genotypes of *TaUBC25* gene in 681 wheat materials. **Table S6.** Association analysis of TKW, KL, KW and KT between two haplotypes of *TaUBC25* gene. **Table S7.** Wheat diversity panel information and distribution of*TaUBC25* alleles among genotypes. **Table S8.** Information on genotypes containing the *TaUBC25* gene in the wheat diversity panel over decades. **Table S9.** The results of the amplification efficiency of each pair of primers.** Table S10 and Table S11.** The details on relative expression calculation and statistical methods for result significance.

## Data Availability

All data generated or analyzed during this study are included in this published article and its supplementary information files. The datasets used and/or analyzed during the current study are available from the corresponding author up on reasonable request.

## Abbreviations

DAA: Days After Anthesis
KL: Kernel length
KT: Kernel thickness
KW: Kernel width
TKW: Thousand-kernel weight
SNP: Single nucleotide polymorphism
UBC: E1 ubiquitin-activating enzyme
UPS: Ubiquitin–proteasome system

## References

[1] Cruz ER, Nguyen H, Nguyen T (2019). Functional analysis tools for post-translational modification: a post-translational modification database for analysis of proteins and metabolic pathways. Plant J.

[2] Pickart CM (2001). Mechanisms underlying ubiquitination. Annu Rev Biochem.

[3] Collins GA, Goldberg AL (2017). The Logic of the 26S Proteasome. Cell.

[4] Vierstra RD (2009). The ubiquitin-26S proteasome system at the nexus of plant biology. Nat Rev Mol Cell Biol.

[5] Gao Y, Wang Y, Xin H (2017). Involvement of ubiquitin-conjugating enzyme (*E2* Gene Family) in ripening process and response to cold and heat stress of vitis vinifera. Sci Rep.

[6] Xu F, Xue H (2019). The ubiquitin-proteasome system in plant responses to environments. Plant Cell Environ.

[7] Stone SL (2019). Role of the ubiquitin proteasome system in plant response to abiotic stress. Int Rev Cell Mol Biol.

[8] Chen K, Tang W, Zhou Y (2020). Overexpression of
* GmUBC9
* gene enhances plant drought resistance and affects flowering time via histone H2B monoubiquitination. Front Plant Sci.

[9] Su T, Yang M, Wang P (2020). Interplay between the ubiquitin proteasome system and ubiquitin-mediated autophagy in plants. Cells.

[10] Bae H, Kim WT (2013). The N-terminal tetra-peptide (IPDE) short extension of the U-box motif in rice SPL11 E3 is essential for the interaction with E2 and ubiquitin-ligase activity. Biochem Biophys Res Commun.

[11] Ye Y, Rape M (2009). Building ubiquitin chains: E2 enzymes at work. Nat Rev Mol Cell Biol.

[12] Bae H, Kim WT (2014). Classification and interaction modes of 40 rice E2 ubiquitin-conjugating enzymes with 17 rice ARM-U-box E3 ubiquitin ligases. Biochem Biophys Res Commun.

[13] van Wijk SJ, Timmers HT (2010). The family of ubiquitin-conjugating enzymes (E2s): deciding between life and death of proteins. Faseb J.

[14] Jue D, Sang X, Liu L (2018). The ubiquitin-conjugating enzyme gene family in Longan (Dimocarpus longan Lour.): Genome-wide identification and gene expression during flower induction and abiotic stress responses. Molecules.

[15] Jia L, Zhao Q, Chen S (2019). Evolution and expression analysis of the sorghum ubiquitin-conjugating enzyme family. Funct Plant Biol.

[16] Liu W, Tang X, Zhu X (2019). Genome-wide identification and expression analysis of the E2 gene family in potato. Mol Biol Rep.

[17] Sharma B, Bhatt TK (2017). Genome-wide identification and expression analysis of E2 ubiquitin-conjugating enzymes in tomato. Sci Rep.

[18] Dong C, Hu H, Jue D (2016). The banana E2 gene family: Genomic identification, characterization, expression profiling analysis. Plant Sci.

[19] Khan N, Hu CM, Amjad Khan W (2018). Evolution and expression divergence of E2 gene family under multiple abiotic and phytohormones stresses in brassica rapa. Biomed Res Int.

[20] Kraft E, Stone SL, Ma L (2005). Genome analysis and functional characterization of the E2 and RING-type E3 ligase ubiquitination enzymes of Arabidopsis. Plant Physiol.

[21] Feussner K, Feussner I, Leopold I (1997). Isolation of a cDNA coding for an ubiquitin-conjugating enzyme UBC1 of tomato–the first stress-induced UBC of higher plants. FEBS Lett.

[22] Cui F, Liu L, Zhao Q (2012). Arabidopsis ubiquitin conjugase UBC32 is an ERAD component that functions in brassinosteroid-mediated salt stress tolerance. Plant Cell.

[23] Ahn MY, Oh TR, Seo DH (2018). Arabidopsis group XIV ubiquitin-conjugating enzymes AtUBC32, AtUBC33, and AtUBC34 play negative roles in drought stress response. J Plant Physiol.

[24] Feng H, Wang S, Dong D (2020). Arabidopsis ubiquitin-conjugating enzymes UBC7, UBC13, and UBC14 are required in plant responses to multiple stress conditions. Plants (Basel).

[25] Xu L, Ménard R, Berr A (2009). The E2 ubiquitin-conjugating enzymes, AtUBC1 and AtUBC2, play redundant roles and are involved in activation of FLC expression and repression of flowering in Arabidopsis thaliana. Plant J.

[26] Lau OS, Deng X (2009). Effect of Arabidopsis COP10 ubiquitin E2 enhancement activity across E2 families and functional conservation among its canonical homologues. Biochem J.

[27] Wen R, Wang S, Xiang D (2014). UBC13, an E2 enzyme for Lys63-linked ubiquitination, functions in root development by affecting auxin signaling and Aux/IAA protein stability. Plant J.

[28] Wang S, Li Q, Zhao L (2020). Arabidopsis UBC22, an E2 able to catalyze lysine-11 specific ubiquitin linkage formation, has multiple functions in plant growth and immunity. Plant Sci.

[29] Wang Y, Wang W, Cai J (2014). Tomato nuclear proteome reveals the involvement of specific E2 ubiquitin-conjugating enzymes in fruit ripening. Genome Biol.

[30] Zhang J, Zhang Z, Zhang R (2024). Type I MADS-box transcription factor *TaMADS-GS* regulates grain size by stabilizing cytokinin signalling during endosperm cellularization in wheat. Plant Biotechnol J.

[31] Marcussen T, Sandve SR, Heier L (2014). Ancient hybridizations among the ancestral genomes of bread wheat. Science.

[32] Zhou Y, Zhao X, Li Y (2020). Triticum population sequencing provides insights into wheat adaptation. Nat Genet.

[33] Brinton J, Uauy C (2019). A reductionist approach to dissecting grain weight and yield in wheat. J Integr Plant Biol.

[34] Cheng H, Liu J, Wen J (2019). Frequent intra-and inter-species introgression shapes the landscape of genetic variation in bread wheat. Genome Biol.

[35] Wang W, Wang Z, Li X (2020). SnpHub: an easy-to-set-up web server framework for exploring large-scale genomic variation data in the post-genomic era with applications in wheat. Gigascience.

[36] Hao C, Jiao C, Hou J (2020). Resequencing of 145 Landmark cultivars reveals asymmetric sub-genome selection and strong founder genotype effects on wheat breeding in China. Mol Plant.

[37] Bailey TL, Boden M, Buske FA (2009). MEME SUITE: tools for motif discovery and searching. Nucleic Acids Res.

[38] Moore RC, Purugganan MD (2005). The evolutionary dynamics of plant duplicate genes. Curr Opin Plant Biol.

[39] Ma L, Li T, Hao C (2016). TaGS5-3A, a grain size gene selected during wheat improvement for larger kernel and yield. Plant Biotechnol J.

[40] Yu K, Feng M, Yang G (2020). Changes in alternative splicing in response to domestication and polyploidization in Wheat. Plant Physiol.

[41] Lai D, Yan J, Fan Y (2021). Genome-wide identification and phylogenetic relationships of the Hsp70 gene family of Aegilops tauschii, wild emmer wheat (Triticum dicoccoides) and bread wheat (Triticum aestivum). 3 Biotech.

[42] Jiang S, Ma Z, Ramachandran S (2010). Evolutionary history and stress regulation of the lectin superfamily in higher plants. BMC Evol Biol.

[43] Hanada K, Zou C, Lehti-shiu MD (2008). Importance of lineage-specific expansion of plant tandem duplicates in the adaptive response to environmental stimuli. Plant Physiol.

[44] Na N, Zhen L, Huang P (2022). Genome-wide identification and expression analysis of potato
* GAUT
* gene family. Acta Agron Sin.

[45] Huang J, Li L, Mao X, et al. dCAPS markers developed for nitrate transporter genes *TaNRT2L12s* associating with 1 000-grain weight in wheat. J Integr Agr. 2020; 19(6):1543–53.

[46] Yu F, Wu Y, Xie Q (2016). Ubiquitin-proteasome system in ABA signaling: From perception to action. Mol Plant.

[47] Lorick KL, Jensen JP, Fang S (1999). RING fingers mediate ubiquitin-conjugating enzyme (E2)-dependent ubiquitination. Proc Natl Acad Sci U S A.

[48] Christensen DE, Klevit RE (2009). Dynamic interactions of proteins in complex networks: identifying the complete set of interacting E2s for functional investigation of E3-dependent protein ubiquitination. Febs j.

[49] E Z, Zhang Y, Li T (2015). Characterization of the ubiquitin-conjugating enzyme gene family in rice and evaluation of expression profiles under abiotic stresses and hormone treatments. PLoS One.

[50] Peng L, Che L, Hao S (2021). ldentification and analysis of non-specific lipid transfer protein family intobacco. Acta Agron Sin.

[51] Errum A, Rehman N, Khan MR (2021). Genome-wide characterization and expression analysis of pseudo-response regulator gene family in wheat. Mol Biol Rep.

[52] Jeon EH, Pak JH, Kim MJ (2012). Ectopic expression of ubiquitin-conjugating enzyme gene from wild rice, *OgUBC1*, confers resistance against UV-B radiation and Botrytis infection in Arabidopsis thaliana. Biochem Biophys Res Commun.

[53] Zhou G, Chang R, Qiu L (2010). Overexpression of soybean ubiquitin-conjugating enzyme gene
* GmUBC2
* confers enhanced drought and salt tolerance through modulating abiotic stress-responsive gene expression in Arabidopsis. Plant Mol Biol.

[54] Tang S, Zhao Z, Liu X (2023). An E2–E3 pair contributes to seed size control in grain crops. Nat Commun.

[55] Liu X, Song L, Zhang H (2021). Rice ubiquitin-conjugating enzyme OsUBC26 is essential for immunity to the blast fungus Magnaporthe oryzae. Mol Plant Pathol.

[56] Wang Y, Yue J, Yang N (2023). An ERAD-related ubiquitin-conjugating enzyme boosts broad-spectrum disease resistance and yield in rice. Nat Food.

[57] Li J, Zhang B, Duan P (2023). An endoplasmic reticulum-associated degradation-related E2–E3 enzyme pair controls grain size and weight through the brassinosteroid signaling pathway in rice. Plant Cell.

[58] Gao X, Zhang JQ, Zhang X (2019). Rice qGL3/OsPPKL1 functions with the GSK3/SHAGGY-Like kinase OsGSK3 to modulate brassinosteroid signaling. Plant Cell.

[59] Yao Y, Ni Z, Zhang Y (2005). Identification of differentially expressed genes in leaf and root between wheat hybrid and its parental inbreds using PCR-based cDNA subtraction. Plant Mol Biol.

[60] Chen X, Zhang Y, Tong Y (2017). Harvesting more grain zinc of wheat for human health. Sci Rep.

[61] El Habti A, Fleury D, Jewell N (2020). Tolerance of combined drought and heat stress is associated with transpiration maintenance and water soluble carbohydrates in wheat grains. Front Plant Sci.

[62] Yoshida T, Fujita Y, Sayama H (2010). AREB1, AREB2, and ABF3 are master transcription factors that cooperatively regulate ABRE-dependent ABA signaling involved in drought stress tolerance and require ABA for full activation. Plant J.

[63] Zheng Z, Yang X, Fu Y (2017). Overexpression of *PvPin1*, a bamboo homolog of PIN1-type Parvulin 1, delays flowering time in transgenic Arabidopsis and rice. Front Plant Sci.

[64] Li R, Zhu F, Duan D (2020). Function analysis and stress-mediated *cis*-element identification in the promoter region of *VqMYB15*. Plant Signal Behav.

[65] Su Z, Hao C, Wang L (2011). Identification and development of a functional marker of TaGW2 associated with grain weight in bread wheat (*Triticum aestivum* L.). Theor Appl Genet.

[66] Maphosa L, Langridge P, Taylor H (2014). Genetic control of grain yield and grain physical characteristics in a bread wheat population grown under a range of environmental conditions. Theor Appl Genet.

[67] Liu H, Li H, Hao C (2020). TaDA1, a conserved negative regulator of kernel size, has an additive effect with TaGW2 in common wheat (*Triticum aestivum* L.). Plant Biotechnol J.

[68] Wang W, Pan Q, Tian B (2019). Gene editing of the wheat homologs of TONNEAU1-recruiting motif encoding gene affects grain shape and weight in wheat. Plant J.

[69] Hou J, Jiang Q, Hao C (2014). Global selection on sucrose synthase haplotypes during a century of wheat breeding. Plant Physiol.

[70] Guo L, Ma M, Wu L (2022). Modified expression of TaCYP78A5 enhances grain weight with yield potential by accumulating auxin in wheat (*Triticum aestivum* L.). Plant Biotechnol J.

[71] Dong B, Zheng X, Liu H (2017). Effects of drought stress on pollen sterility, grain yield, abscisic acid and protective enzymes in two winter wheat cultivars. Front Plant Sci.

[72] Yang J, Zhu J, Wang S (2013). Drought-resistance of local wheat varieties in Shanxi Province of China: a comprehensive evaluation by using GGE biplot and subordinate function. Ying Yong Sheng Tai Xue Bao.

[73] Zhang P, Zhang L, Chen T (2022). Genome-wide identification and expression analysis of the GSK gene family in wheat (Triticum aestivum L.). Mol Biol Rep.

[74] Finn RD, Mistry J, Schuster-böckler B (2006). Pfam: clans, web tools and services. Nucleic Acids Res.

[75] Eddy SR (2011). Accelerated Profile HMM Searches. PLoS Comput Biol.

[76] El-gebali S, Mistry J, Bateman A (2019). The Pfam protein families database in 2019. Nucleic Acids Res.

[77] Marchler-bauer A, Derbyshire MK, Gonzales NR (2015). CDD: NCBI's conserved domain database. Nucleic Acids Res.

[78] Letunic I, Khedkar S, Bork P (2021). SMART: recent updates, new developments and status in 2020. Nucleic Acids Res.

[79] Youn J, Kim T (2015). Functional insights of plant GSK3-like kinases: multi-taskers in diverse cellular signal transduction pathways. Mol Plant.

[80] Chou K, Shen H (2010). Plant-mPLoc: a top-down strategy to augment the power for predicting plant protein subcellular localization. PLoS One.

[81] Kumar S, Stecher G, Tamura K (2016). MEGA7: Molecular evolutionary genetics analysis version 7.0 for bigger datasets. Mol Biol Evol.

[82] Li J, Wang T, Han J (2020). Genome-wide identification and characterization of cucumber bHLH family genes and the functional characterization of *CsbHLH041* in NaCl and ABA tolerance in Arabidopsis and cucumber. BMC Plant Biol.

[83] Xie J, Chen Y, Cai G (2023). Tree Visualization By One Table (tvBOT): a web application for visualizing, modifying and annotating phylogenetic trees. Nucleic Acids Res.

[84] Chen C, Chen H, Zhang Y (2020). TBtools: An integrative toolkit developed for interactive analyses of big biological data. Mol Plant.

[85] Zhang Z, Li J, Zhao X (2006). KaKs_Calculator: calculating Ka and Ks through model selection and model averaging. Genomics Proteomics Bioinformatics.

[86] Lai W, Zhou Y, Pan R (2020). Identification and expression analysis of stress-associated proteins (SAPs) Containing A20/AN1 Zinc Finger in Cucumber. Plants (Basel).

[87] Wang Y, Li J, Paterson AH (2013). MCScanX-transposed: detecting transposed gene duplications based on multiple colinearity scans. Bioinformatics.

[88] Borrill P, Ramirez-Gonzalez R, Uauy C (2016). expVIP: a Customizable RNA-seq data analysis and visualization platform. Plant Physiol.

[89] Ramírez-González RH, Borrill P, Lang D (2018). The transcriptional landscape of polyploid wheat. Science.

[90] Pertea M, Pertea GM, Antonescu CM (2015). StringTie enables improved reconstruction of a transcriptome from RNA-seq reads. Nat Biotechnol.

[91] Wagner GP, Kin K, Lynch VJ (2013). A model based criterion for gene expression calls using RNA-seq data. Theory Biosci.

[92] Mei F, Chen B, Du L (2022). A gain-of-function allele of a DREB transcription factor gene ameliorates drought tolerance in wheat. Plant Cell.

[93] He J, Li C, Hu N (2022). ECERIFERUM1-6A is required for the synthesis of cuticular wax alkanes and promotes drought tolerance in wheat. Plant Physiol.

[94] Kobayashi F, Takumi S, Handa H (2010). Identification of quantitative trait loci for ABA responsiveness at the seedling stage associated with ABA-regulated gene expression in common wheat. Theor Appl Genet.

[95] Schmittgen TD, Livak KJ (2008). Analyzing real-time PCR data by the comparative C(T) method. Nat Protoc.

[96] Guo W, Xin M, Wang Z (2020). Origin and adaptation to high altitude of Tibetan semi-wild wheat. Nat Commun.

[97] Walkowiak S, Gao L, Monat C (2020). Multiple wheat genomes reveal global variation in modern breeding. Nature.

[98] Yang Z, Wang Z, Wang W (2022). ggComp enables dissection of germplasm resources and construction of a multiscale germplasm network in wheat. Plant Physiol.

