# Supplementary material for: Genome‑wide identification and expression analysis of the UBC gene family in wheat (Triticum aestivum L.)
Source: BMC Plant Biol. 2024 Apr 26;24:341. doi: 10.1186/s12870-024-05042-3 (PMC11047035; doi:10.1186/s12870-024-05042-3)
Supplement: Supplementary file 1 — Additional file 1: Figure S1. Phylogenetic relationships, gene structure and conserved motifs of TaUBC genes in wheat. A: The different color blocks indicated different subclasses of the TaUBC genes family. B: The distribution of conserved motifs constructed by the MEME. Boxes with different colors represent different conserved motifs (Motif 1–10) C: Structure of TaUBC genes, with blue boxes representing UTRs, yellow boxes representing exons, and black lines representing introns. Figure S2. Conserved domains of TaUBCs in wheat. Figure S3. Collinearity and chromosome localization analysis of TaUBCs. A: Collinearity analysis of UBCs between wheat and Arabidopsis, rice, which were constructed by TBtools. The gray lines in the background indicate the orthologous genes of wheat and the other two species, while the blue lines highlight the collinear TaUBC gene pairs of wheat and the other two species. B: Distribution of TaUBC genes on chromosomes. Figure S4. Expression heatmap of TaUBC genes under different abiotic stress in Chinese spring. The heatmap shows the phylogenetic clustering of 93 TaUBC genes. Color scale: Blue represents low expression and red represents high expression levels. Figure S5. Analysis of cis-elements in the promoter of TaUBC genes. Figure S6. qRT-PCR analysis of selected TaUBCs under 200 mM NaCl, 20% PEG6000, and 100 uM ABA stress. A: Relative expression patterns of TaUBCs in leaves after 200 mM NaCl treatment. B: Relative expression patterns of TaUBCs in leaves after 20% PEG6000 treatment. C: Relative expression patterns of TaUBCs in leaves after treatment with 100 uM ABA. Relative expression values in the control sample (CK 0h) were normalized to 1. TaAct was used as a reference gene. Each bar value is the average value ± standard deviation based on three biological replicates. The different letters denote a significant difference between means (P < 0.05). Table S1. Characteristics of TaUBC genes family members. Table S2. Information on duplicat [file 12870_2024_5042_MOESM1_ESM.zip › Supplemental materials.docx]

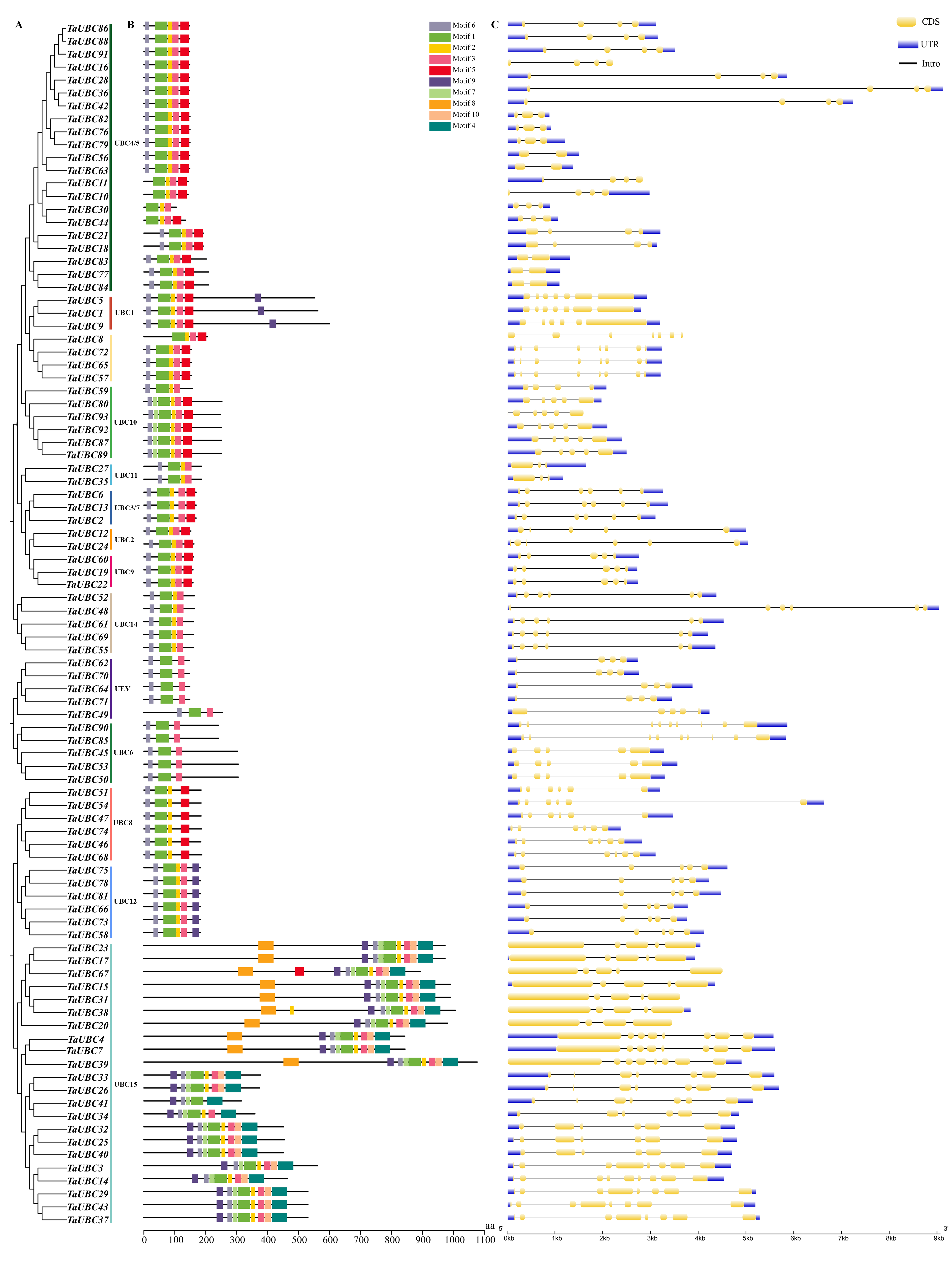
**SUPPLEMENTAL MATERIALS**

**Figure S1.** Phylogenetic relationships, gene structure and conserved motifs of *TaUBC* genes in wheat. **A:** The different color blocks indicated different subclasses of the *TaUBC* genes family. **B:** The distribution of conserved motifs constructed by the MEME. Boxes with different colors represent different conserved motifs (Motif 1–10). **C:** Structure of *TaUBC* genes, with blue boxes representing UTRs, yellow boxes representing exons, and black lines representing introns.

**
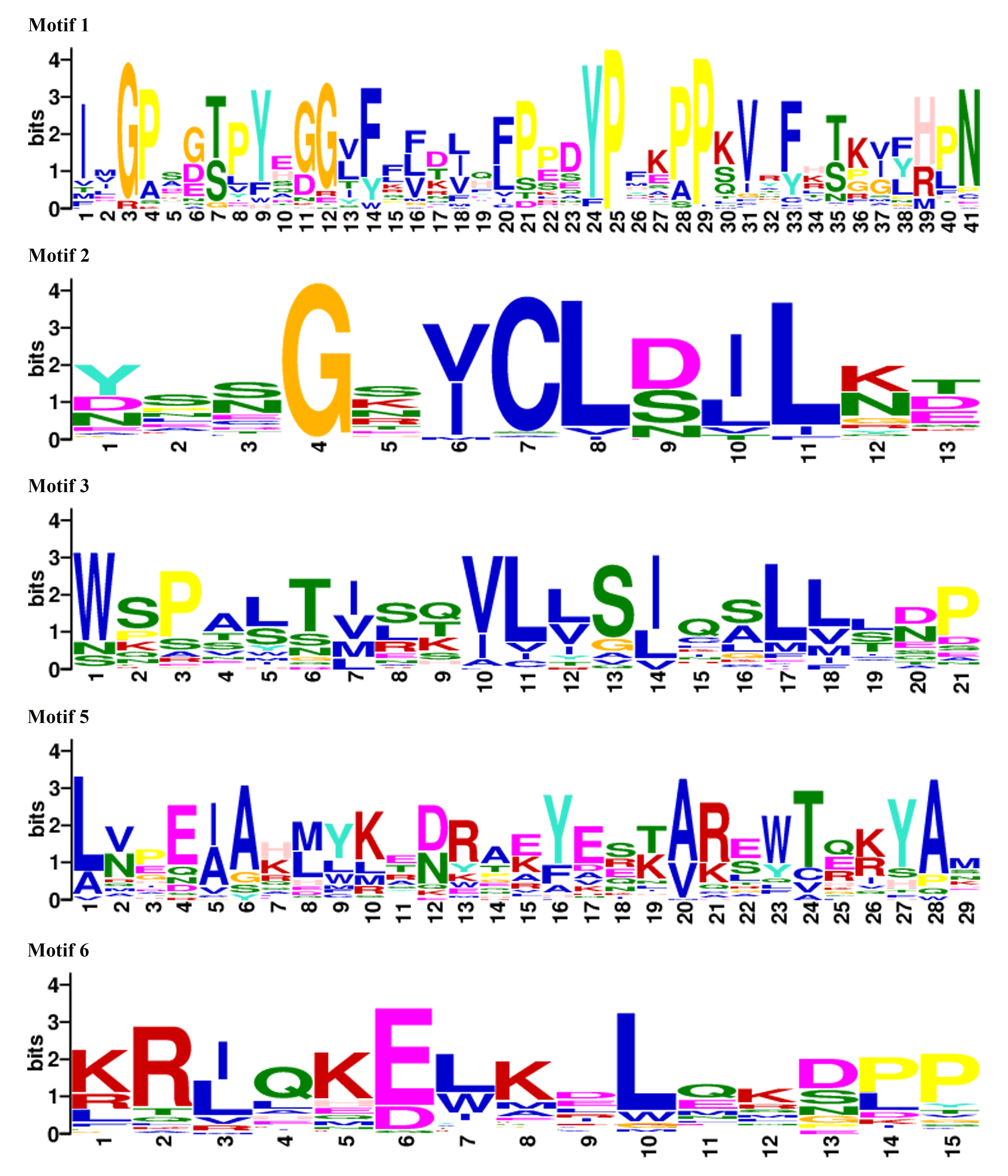
Figure S2.** Conserved domains of *TaUBCs* in wheat

**
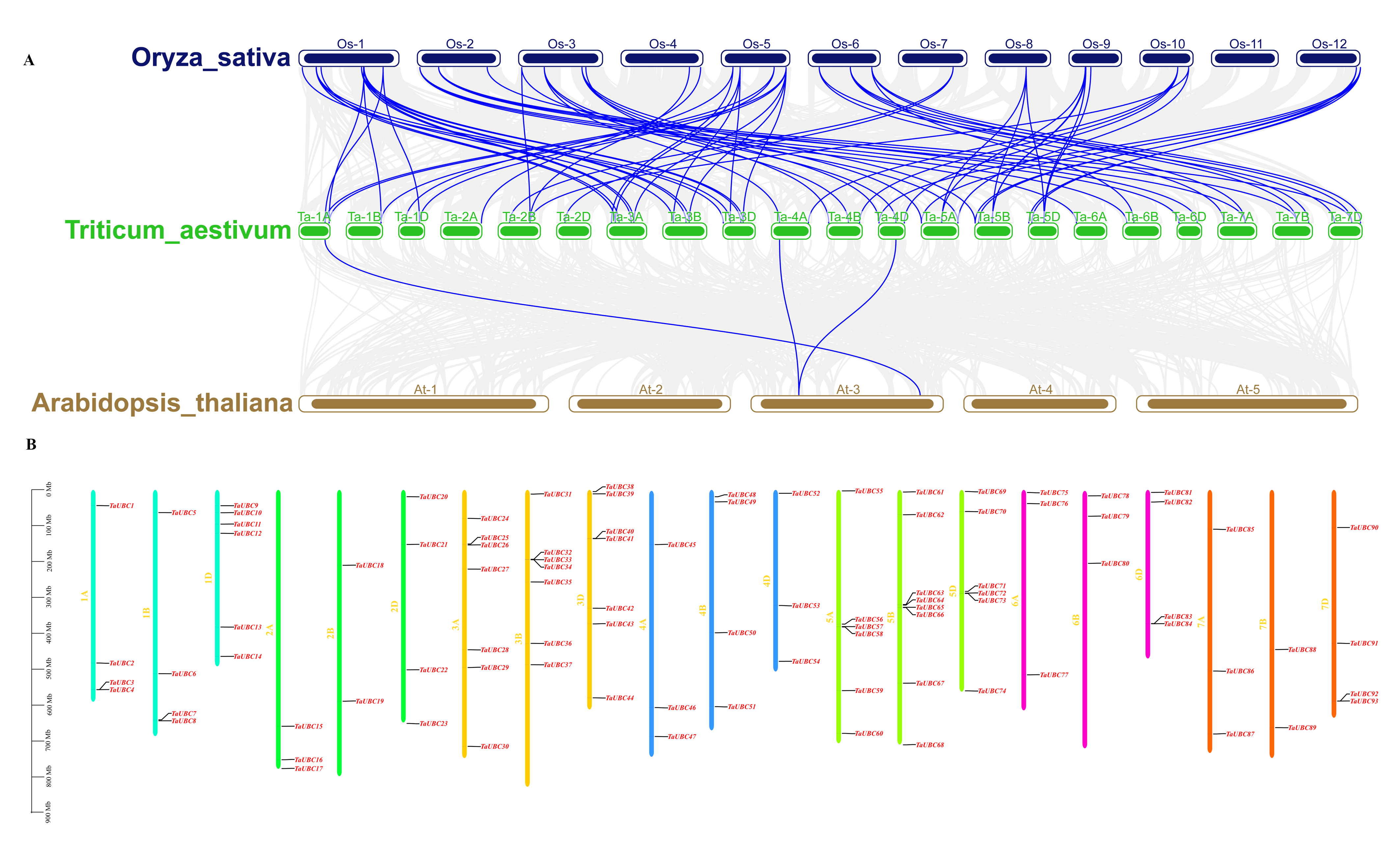
Figure S3.** Collinearity and chromosome localization analysis of *TaUBCs.* **A:** Collinearity analysis of *UBCs* between wheat and *Arabidopsis*, rice, which were constructed by TBtools. The gray lines in the background indicate the orthologous genes of wheat and the other two species, while the blue lines highlight the collinear *TaUBC* gene pairs of wheat and the other two species. **B:** Distribution of *TaUBC* genes on chromosomes.

**
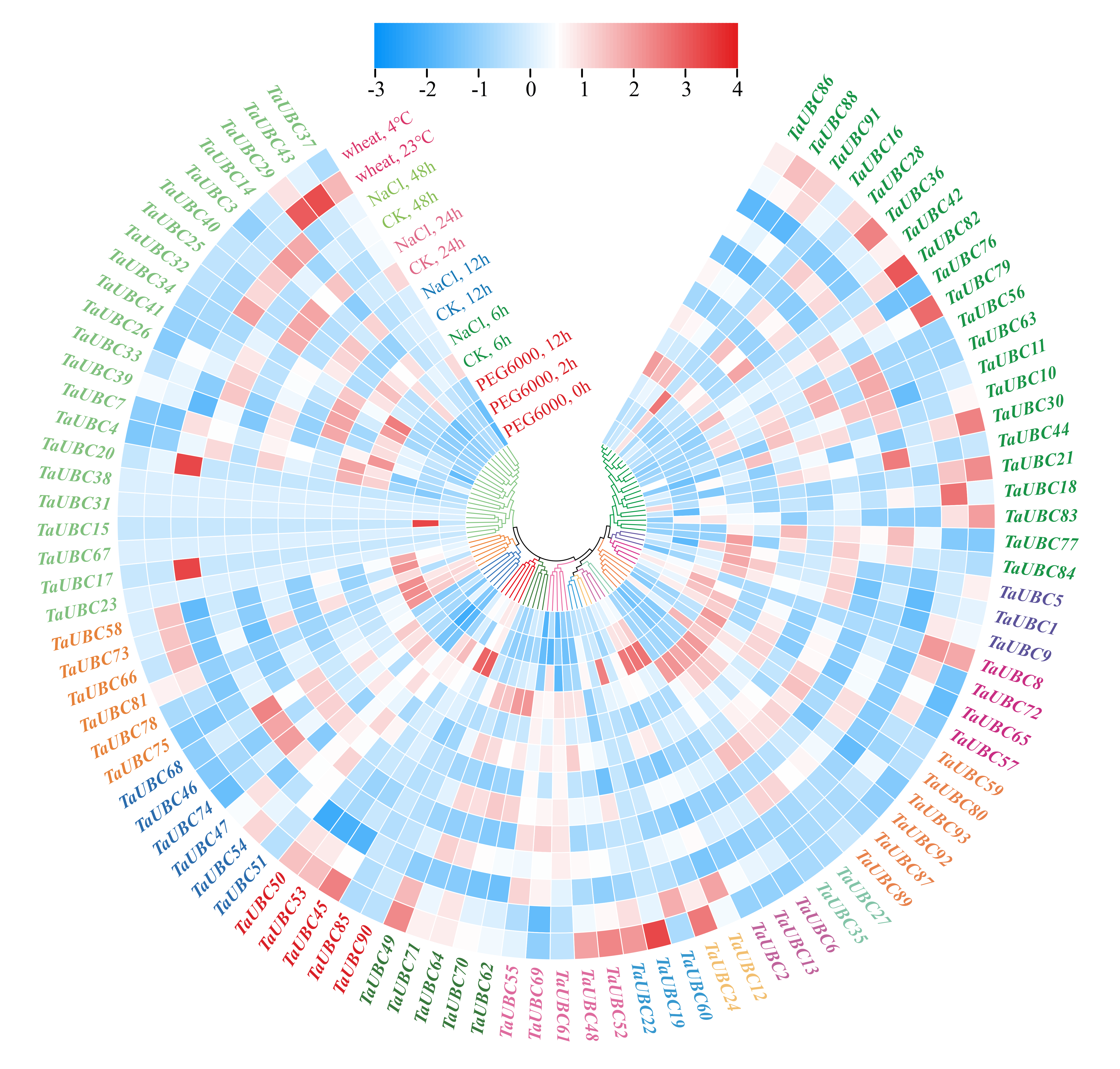
Figure S4.** Expression heatmap of *TaUBC* genes under different abiotic stress in Chinese spring. The heatmap shows the phylogenetic clustering of 93 *TaUBC* genes. Color scale: Blue represents low expression and red represents high expression level.

**
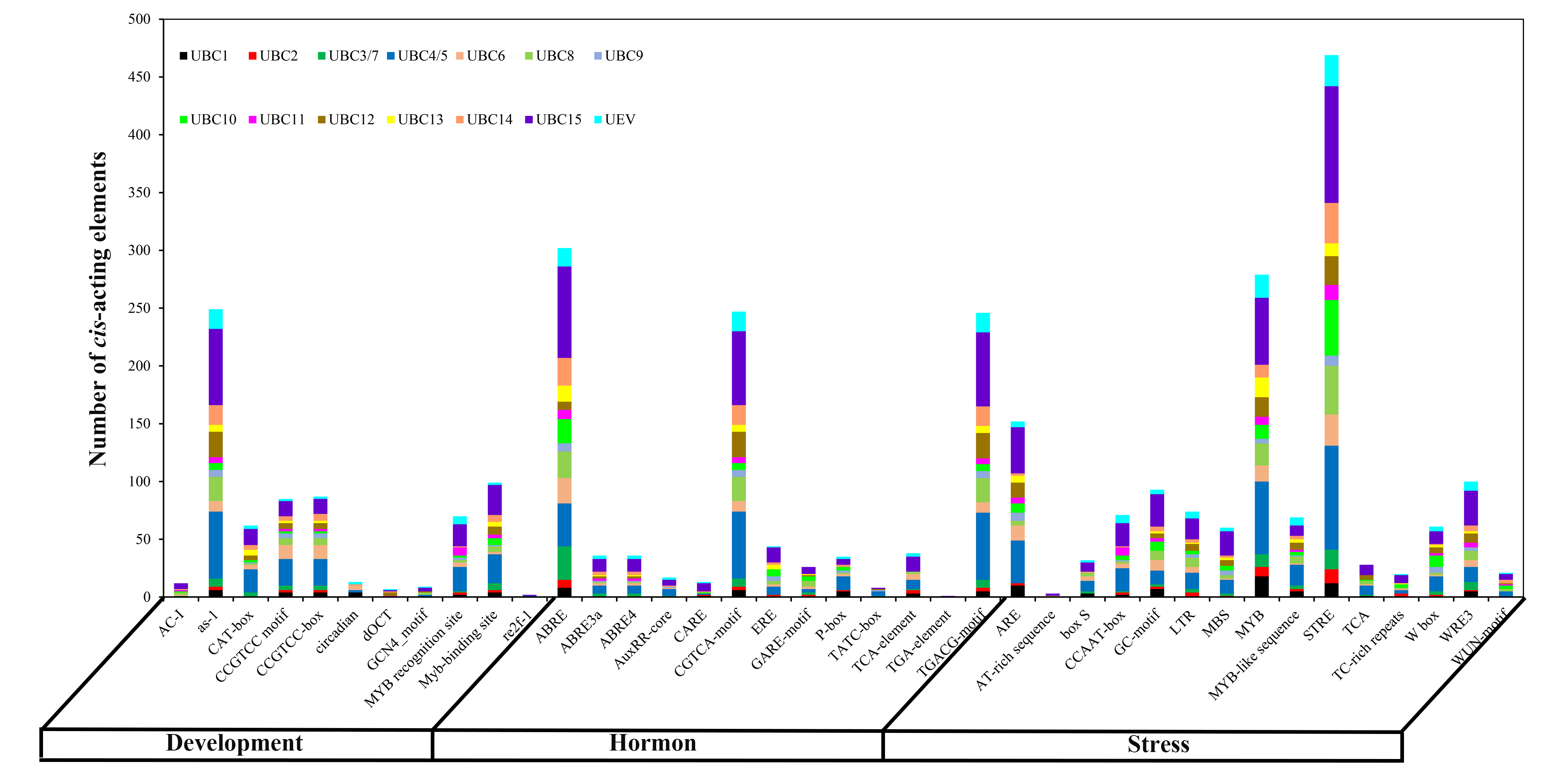
Figure S5.** Analysis of *cis*-elements in the promoter of *TaUBC* genes.

**Figure S6.** qRT-PCR analysis of selected *TaUBCs* under 200 mM NaCl, 20% PEG6000, and 100 uM ABA stress. A: Relative expression patterns of *TaUBCs* in leaves after 200 mM NaCl treatment. B: Relative expression patterns of *TaUBCs* in leaves after 20% PEG6000 treatment. C: Relative expression patterns of *TaUBCs* in leaves after treatment with 100 uM ABA. Relative expression values in the control sample (CK 0h) were normalized to 1. *TaActin3*/*TaAct* was used as a reference gene. Each bar value is the average value ± standard deviation based on three biological replicates. The different letters denote a significant difference between means (*P* < 0.05)
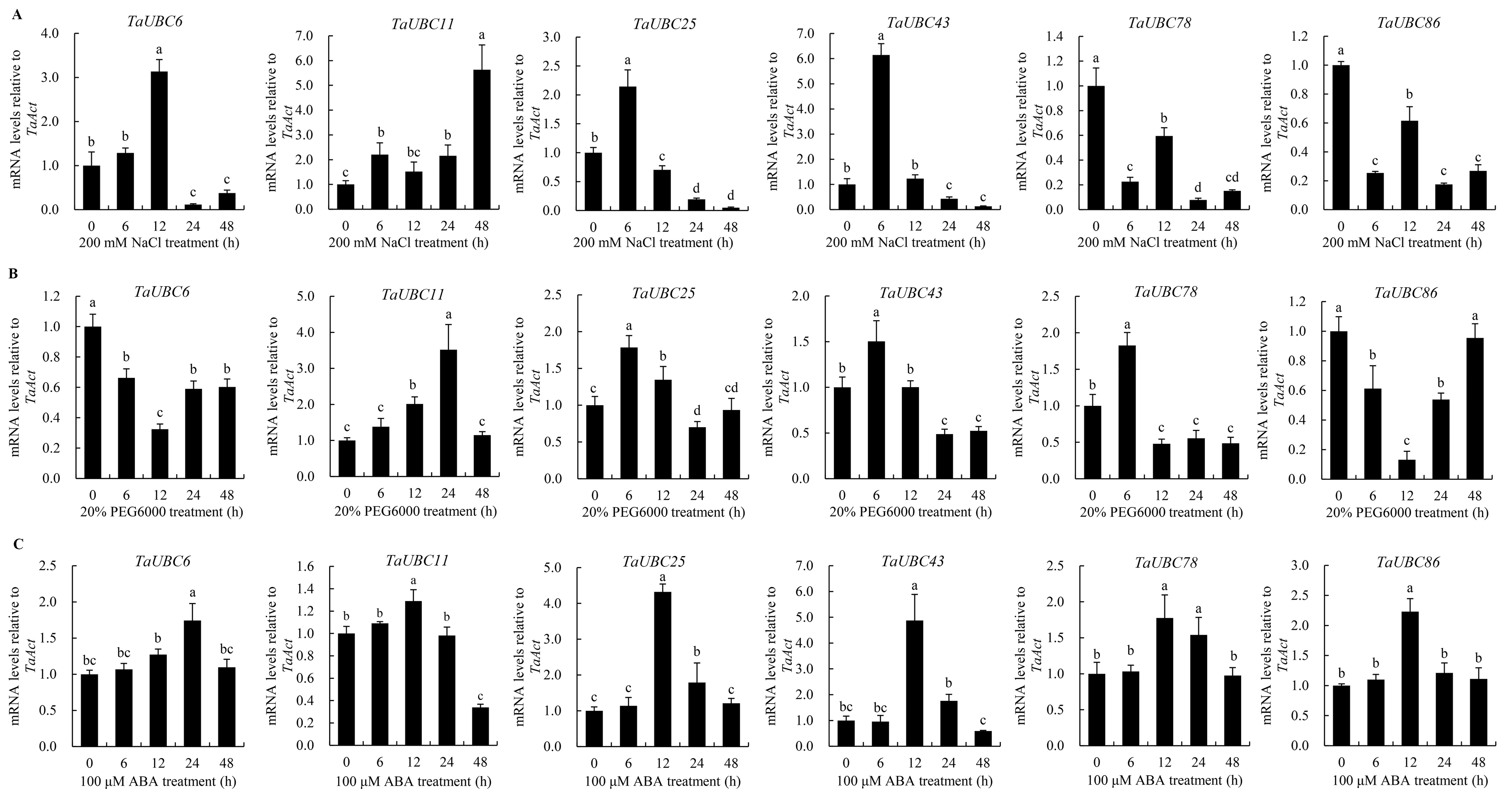


**Table S1**. Characteristics of *TaUBC* genes family members.

| **Gene ID** | **Gene Name** | **Chromosome location** | **Gene Size(bp)** | **pl** | **Mw (kD)** | **GRAVY** | **Intron** | **Subcellular localization** |
| --- | --- | --- | --- | --- | --- | --- | --- | --- |
| TraesCS1A02G063600 | *TaUBC1* | 1A:44408101-44411901 | 1689 | 8.46 | 61.75 | -0.65 | 6 | nucleus |
| TraesCS1A02G285200 | *TaUBC2* | 1A:482758866-482761946 | 510 | 5.04 | 18.89 | -0.43 | 5 | chloroplast |
| TraesCS1A02G388000 | *TaUBC3* | 1A:556796077-556800728 | 1686 | 5.51 | 62.37 | -0.51 | 7 | mitochondria |
| TraesCS1A02G388400 | *TaUBC4* | 1A:556938246-556945562 | 2532 | 4.74 | 93.73 | -0.24 | 7 | nucleus/ cytoplasm |
| TraesCS1B02G081500 | *TaUBC5* | 1B:63960700-63964607 | 1659 | 8.99 | 60.61 | -0.64 | 6 | nucleus |
| TraesCS1B02G294400 | *TaUBC6* | 1B:512419275-512422511 | 510 | 5.04 | 18.89 | -0.43 | 5 | chloroplast |
| TraesCS1B02G415800 | *TaUBC7* | 1B:640845168-640852534 | 2535 | 4.8 | 93.74 | -0.22 | 7 | nucleus |
| TraesCS1B02G420600 | *TaUBC8* | 1B:643587156-643590805 | 618 | 8.95 | 23.26 | -0.05 | 6 | vacuole |
| TraesCS1D02G064100 | *TaUBC9* | 1D:44643108-44647221 | 1803 | 8.99 | 65.88 | -0.65 | 5 | nucleus |
| TraesCS1D02G082400 | *TaUBC10* | 1D:64335489-64338446 | 432 | 5.61 | 16.01 | -0.13 | 3 | nucleus |
| TraesCS1D02G104600 | *TaUBC11* | 1D:96034437-96037255 | 432 | 5.3 | 15.80 | -0.09 | 3 | Extracellular |
| TraesCS1D02G121800 | *TaUBC12* | 1D:121619178-121626281 | 459 | 5.37 | 17.32 | -0.54 | 4 | nucleus |
| TraesCS1D02G284200 | *TaUBC13* | 1D:382632558-382635902 | 510 | 5.04 | 18.89 | -0.43 | 5 | chloroplast |
| TraesCS1D02G396000 | *TaUBC14* | 1D:464322487-464326997 | 1395 | 8.37 | 51.14 | -0.39 | 8 | mitochondria |
| TraesCS2A02G403400 | *TaUBC15* | 2A:658906258-658910585 | 2976 | 4.83 | 108.64 | -0.34 | 4 | nucleus |
| TraesCS2A02G539800 | *TaUBC16* | 2A:752092926-752095121 | 447 | 7.72 | 16.55 | -0.33 | 3 | nucleus |
| TraesCS2A02G586300 | *TaUBC17* | 2A:776600150-776604053 | 2922 | 4.94 | 106.29 | -0.33 | 4 | nucleus |
| TraesCS2B02G220200 | *TaUBC18* | 2B:210663098-210666214 | 579 | 7.77 | 20.27 | -0.02 | 3 | chloroplast |
| TraesCS2B02G413400 | *TaUBC19* | 2B:589089255-589091957 | 480 | 9.06 | 17.98 | -0.60 | 4 | peroxisome |
| TraesCS2D02G051900 | *TaUBC20* | 2D:19643248-19646678 | 2946 | 5.14 | 107.82 | -0.29 | 3 | cytomembrane |
| TraesCS2D02G200900 | TaUBC21 | 2D:152658075-152661258 | 579 | 7.77 | 20.17 | -0.08 | 3 | chloroplast |
| TraesCS2D02G393100 | *TaUBC22* | 2D:501668330-501671050 | 480 | 9.08 | 18.03 | -0.56 | 4 | peroxisome |
| TraesCS2D02G600300 | *TaUBC23* | 2D:651068017-651072034 | 2922 | 4.79 | 105.97 | -0.32 | 4 | nucleus |
| TraesCS3A02G112600 | *TaUBC24* | 3A:80088519-80093525 | 489 | 7.76 | 18.66 | -0.53 | 5 | chloroplast |
| TraesCS3A02G156300 | *TaUBC25* | 3A:151314356-151319141 | 1365 | 4.76 | 50.93 | -0.74 | 6 | nucleus |
| TraesCS3A02G157200 | *TaUBC26* | 3A:154032915-154038572 | 1125 | 8.93 | 41.57 | -0.42 | 6 | nucleus |
| TraesCS3A02G186500 | *TaUBC27* | 3A:221277371-221279002 | 561 | 5.15 | 19.86 | -0.38 | 2 | nucleus |
| TraesCS3A02G238400 | *TaUBC28* | 3A:445865032-445870854 | 444 | 7.69 | 16.56 | -0.39 | 3 | nucleus |
| TraesCS3A02G269200 | *TaUBC29* | 3A:495251709-495256881 | 1593 | 5.51 | 58.76 | -0.34 | 7 | nucleus |
| TraesCS3A02G487000 | *TaUBC30* | 3A:714787620-714789640 | 318 | 6.56 | 12.05 | -0.02 | 2 | cytoplasm |
| TraesCS3B02G029000 | *TaUBC31* | 3B:12472486-12476081 | 2973 | 4.74 | 109.68 | -0.30 | 4 | nucleus |
| TraesCS3B02G183000 | *TaUBC32* | 3B:193957721-193962456 | 1359 | 4.68 | 50.62 | -0.74 | 5 | nucleus |
| TraesCS3B02G183600 | *TaUBC33* | 3B:194760094-194765652 | 1134 | 8.85 | 41.77 | -0.41 | 6 | nucleus |
| TraesCS3B02G183800 | *TaUBC34* | 3B:195098448-195103274 | 1080 | 9.14 | 40.10 | -0.37 | 5 | nucleus |
| TraesCS3B02G216000 | *TaUBC35* | 3B:257102044-257103200 | 561 | 5.15 | 19.86 | -0.38 | 2 | nucleus |
| TraesCS3B02G266900 | *TaUBC36* | 3B:428089317-428098389 | 444 | 7.69 | 16.56 | -0.39 | 3 | nucleus |
| TraesCS3B02G303300 | *TaUBC37* | 3B:487493622-487498873 | 1593 | 5.51 | 58.79 | -0.34 | 7 | nucleus |
| TraesCS3D02G015300 | *TaUBC38* | 3D:5406391-5410203 | 3021 | 4.82 | 111.06 | -0.33 | 4 | nucleus |
| TraesCS3D02G032000 | *TaUBC39* | 3D:11758089-11762963 | 3234 | 4.53 | 118.59 | -0.52 | 7 | nucleus |
| TraesCS3D02G164200 | *TaUBC40* | 3D:135731657-135736326 | 1356 | 4.67 | 50.66 | -0.73 | 5 | nucleus |
| TraesCS3D02G164900 | *TaUBC41* | 3D:136414720-136419826 | 948 | 8.96 | 35.02 | -0.47 | 6 | nucleus |
| TraesCS3D02G238700 | *TaUBC42* | 3D:330267649-330274849 | 444 | 7.69 | 16.56 | -0.39 | 3 | nucleus |
| TraesCS3D02G269000 | *TaUBC43* | 3D:373805548-373810711 | 1593 | 5.58 | 58.82 | -0.35 | 7 | nucleus |
| TraesCS3D02G482300 | *TaUBC44* | 3D:579687042-579689173 | 408 | 6.94 | 15.39 | -0.16 | 2 | chloroplast |
| TraesCS4A02G121400 | *TaUBC45* | 4A:150227302-150230566 | 912 | 6.03 | 33.09 | -0.36 | 4 | E.R. |
| TraesCS4A02G313600 | *TaUBC46* | 4A:604101159-604103952 | 555 | 4.4 | 21.13 | -0.64 | 5 | nucleus/ cytoplasm |
| TraesCS4A02G414200 | *TaUBC47* | 4A:684660858-684664306 | 558 | 4.19 | 21.01 | -0.60 | 5 | chloroplast/ cytoplasm |
| TraesCS4B02G026000 | *TaUBC48* | 4B:19719798-19728793 | 492 | 6.95 | 18.47 | -0.47 | 5 | mitochondria |
| TraesCS4B02G046900 | *TaUBC49* | 4B:34033677-34037883 | 765 | 8.27 | 28.11 | -0.44 | 4 | chloroplast |
| TraesCS4B02G181500 | *TaUBC50* | 4B:398556848-398560120 | 918 | 6.12 | 33.42 | -0.38 | 4 | E.R. |
| TraesCS4B02G314900 | *TaUBC51* | 4B:604203797-604206975 | 558 | 4.21 | 20.97 | -0.59 | 5 | nucleus |
| TraesCS4D02G023400 | *TaUBC52* | 4D:10240317-10244668 | 492 | 6.95 | 18.47 | -0.47 | 5 | mitochondria |
| TraesCS4D02G184500 | *TaUBC53* | 4D:322673339-322676876 | 918 | 6.12 | 33.42 | -0.37 | 4 | E.R. |
| TraesCS4D02G312000 | *TaUBC54* | 4D:478032965-478039565 | 558 | 4.21 | 21.00 | -0.60 | 5 | nucleus |
| TraesCS5A02G006200 | *TaUBC55* | 5A:3524460-3528789 | 486 | 7.7 | 18.24 | -0.48 | 5 | nucleus |
| TraesCS5A02G177100 | *TaUBC56* | 5A:374476464-374478950 | 447 | 6.82 | 16.56 | -0.44 | 1 | nucleus |
| TraesCS5A02G181700 | *TaUBC57* | 5A:381301631-381304818 | 462 | 6.14 | 17.29 | -0.36 | 7 | mitochondria |
| TraesCS5A02G182800 | *TaUBC58* | 5A:382334601-382338696 | 552 | 8.36 | 20.64 | -0.65 | 4 | cytoplasm |
| TraesCS5A02G357500 | *TaUBC59* | 5A:559579387-559583941 | 474 | 8.36 | 17.83 | -0.25 | 3 | cytoplasm |
| TraesCS5A02G515400 | *TaUBC60* | 5A:678625001-678627740 | 486 | 8.43 | 18.07 | -0.53 | 4 | nucleus |
| TraesCS5B02G005100 | *TaUBC61* | 5B:6650056-6654556 | 486 | 7.7 | 18.24 | -0.48 | 5 | nucleus |
| TraesCS5B02G062300 | *TaUBC62* | 5B:69861189-69863897 | 441 | 6.42 | 16.71 | -0.58 | 3 | cytoplasm |
| TraesCS5B02G174300 | *TaUBC63* | 5B:319904135-319906452 | 447 | 6.82 | 16.52 | -0.45 | 1 | nucleus |
| TraesCS5B02G175500 | *TaUBC64* | 5B:321529843-321533695 | 447 | 6.51 | 16.67 | -0.52 | 3 | chloroplast |
| TraesCS5B02G179700 | *TaUBC65* | 5B:327598483-327601705 | 462 | 6.14 | 17.29 | -0.36 | 7 | mitochondria |
| TraesCS5B02G180900 | *TaUBC66* | 5B:328371308-328375058 | 552 | 8.68 | 20.61 | -0.64 | 4 | cytoplasm |
| TraesCS5B02G359000 | *TaUBC67* | 5B:538934360-538938839 | 2682 | 4.59 | 97.29 | -0.25 | 4 | nucleus |
| TraesCS5B02G566100 | *TaUBC68* | 5B:710609860-710612942 | 564 | 4.34 | 21.51 | -0.69 | 5 | nucleus |
| TraesCS5D02G006000 | *TaUBC69* | 5D:5170287-5174463 | 486 | 7.7 | 18.24 | -0.48 | 5 | nucleus |
| TraesCS5D02G065400 | *TaUBC70* | 5D:60892442-60895183 | 441 | 6.42 | 16.71 | -0.58 | 3 | cytoplasm |
| TraesCS5D02G182300 | *TaUBC71* | 5D:283341442-283344863 | 447 | 6.51 | 16.67 | -0.52 | 3 | chloroplast |
| TraesCS5D02G186100 | *TaUBC72* | 5D:288174517-288177725 | 462 | 6.14 | 17.29 | -0.36 | 7 | mitochondria |
| TraesCS5D02G187200 | *TaUBC73* | 5D:289078120-289081854 | 552 | 8.36 | 20.64 | -0.65 | 4 | cytoplasm |
| TraesCS5D02G558500 | *TaUBC74* | 5D:560226208-560228561 | 561 | 4.31 | 21.37 | -0.67 | 5 | cytoplasm |
| TraesCS6A02G016200 | *TaUBC75* | 6A:7997318-8002713 | 552 | 8.36 | 20.67 | -0.64 | 4 | cytoplasm |
| TraesCS6A02G071100 | *TaUBC76* | 6A:38769595-38770499 | 450 | 8.36 | 16.61 | -0.29 | 2 | nucleus |
| TraesCS6A02G284200 | *TaUBC77* | 6A:515643076-515644173 | 630 | 9.77 | 23.29 | -0.21 | 1 | chloroplast |
| TraesCS6B02G023900 | *TaUBC78* | 6B:14522066-14527173 | 552 | 8.36 | 20.67 | -0.64 | 4 | cytoplasm |
| TraesCS6B02G095200 | *TaUBC79* | 6B:71308235-71309436 | 450 | 8.36 | 16.61 | -0.29 | 2 | nucleus |
| TraesCS6B02G181200 | *TaUBC80* | 6B:202541170-202544929 | 759 | 9.02 | 27.30 | -0.42 | 4 | nucleus |
| TraesCS6D02G019200 | *TaUBC81* | 6D:7735095-7740456 | 552 | 8.36 | 20.67 | -0.64 | 4 | cytoplasm |
| TraesCS6D02G069000 | *TaUBC82* | 6D:34540596-34541467 | 450 | 8.36 | 16.61 | -0.29 | 2 | nucleus |
| TraesCS6D02G264400 | *TaUBC83* | 6D:372949875-372951174 | 609 | 9.59 | 23.03 | -0.22 | 1 | cytoplasm |
| TraesCS6D02G264600 | *TaUBC84* | 6D:373391397-373392476 | 630 | 9.72 | 23.42 | -0.15 | 1 | cytoplasm |
| TraesCS7A02G157200 | *TaUBC85* | 7A:110252382-110258176 | 726 | 8.87 | 27.08 | -0.31 | 8 | cytoplasm |
| TraesCS7A02G343800 | *TaUBC86* | 7A:505022624-505025714 | 447 | 7.72 | 16.49 | -0.29 | 3 | nucleus |
| TraesCS7A02G491300 | *TaUBC87* | 7A:680341311-680345280 | 756 | 9.25 | 27.42 | -0.45 | 4 | cytoplasm |
| TraesCS7B02G239300 | *TaUBC88* | 7B:445287967-445291092 | 447 | 7.72 | 16.49 | -0.29 | 3 | nucleus |
| TraesCS7B02G395100 | *TaUBC89* | 7B:662674333-662678337 | 756 | 9.23 | 27.34 | -0.40 | 4 | cytoplasm |
| TraesCS7D02G157700 | *TaUBC90* | 7D:105428672-105434501 | 726 | 8.87 | 26.99 | -0.30 | 8 | cytoplasm |
| TraesCS7D02G335800 | *TaUBC91* | 7D:427620683-427624173 | 447 | 7.72 | 16.49 | -0.29 | 3 | nucleus |
| TraesCS7D02G477600 | *TaUBC92* | 7D:588554001-588557303 | 756 | 9.02 | 27.35 | -0.41 | 4 | nucleus |
| TraesCS7D02G477900 | *TaUBC93* | 7D:588696830-588698411 | 744 | 9.52 | 26.82 | -0.38 | 5 | chloroplast/ nucleus |

**Note:** Mw, Molecular weight; pi, Isoelectric point; GRAVY, Grand average of Hydropathy index.

**Table S2.** Information on duplication events of *TaUBC* genes.

| **No.** | **Segmental duplication** | | **Ka** | **Ks** | **Ka/Ks** | **Group** |
| --- | --- | --- | --- | --- | --- | --- |
| 1 | TaUBC12 | TaUBC24 | 0.027 | 0.913 | 0.030 | UBC2 |
| 2 | TaUBC2 | TaUBC13 | 0.000 | 0.054 | 0.000 | UBC3/7 |
| 3 | TaUBC18 | TaUBC21 | 0.014 | 0.057 | 0.249 | UBC4/5 |
| 4 | TaUBC28 | TaUBC36 | 0.000 | 0.117 | 0.000 | UBC4/5 |
| 5 | TaUBC30 | TaUBC44 | 0.012 | 0.045 | 0.275 | UBC4/5 |
| 6 | TaUBC28 | TaUBC42 | 0.000 | 0.105 | 0.000 | UBC4/5 |
| 7 | TaUBC36 | TaUBC42 | 0.000 | 0.094 | 0.000 | UBC4/5 |
| 8 | TaUBC56 | TaUBC63 | 0.006 | 0.051 | 0.115 | UBC4/5 |
| 9 | TaUBC57 | TaUBC65 | 0.000 | 0.029 | 0.000 | UBC13 |
| 10 | TaUBC57 | TaUBC72 | 0.000 | 0.049 | 0.000 | UBC13 |
| 11 | TaUBC65 | TaUBC72 | 0.000 | 0.059 | 0.000 | UBC13 |
| 12 | TaUBC76 | TaUBC79 | 0.000 | 0.010 | 0.000 | UBC4/5 |
| 13 | TaUBC77 | TaUBC84 | 0.035 | 0.121 | 0.291 | UBC4/5 |
| 14 | TaUBC76 | TaUBC82 | 0.000 | 0.010 | 0.000 | UBC4/5 |
| 15 | TaUBC79 | TaUBC82 | 0.000 | 0.019 | 0.000 | UBC4/5 |
| 16 | TaUBC86 | TaUBC88 | 0.000 | 0.166 | 0.000 | UBC4/5 |
| 17 | TaUBC86 | TaUBC91 | 0.000 | 0.118 | 0.000 | UBC4/5 |
| 18 | TaUBC88 | TaUBC91 | 0.000 | 0.041 | 0.000 | UBC4/5 |
| 19 | TaUBC45 | TaUBC50 | 0.016 | 0.183 | 0.088 | UBC6 |
| 20 | TaUBC45 | TaUBC53 | 0.012 | 0.177 | 0.066 | UBC6 |
| 21 | TaUBC50 | TaUBC53 | 0.009 | 0.099 | 0.087 | UBC6 |
| 22 | TaUBC85 | TaUBC90 | 0.009 | 0.050 | 0.180 | UBC6 |
| 23 | TaUBC47 | TaUBC51 | 0.005 | 0.144 | 0.032 | UBC8 |
| 24 | TaUBC47 | TaUBC54 | 0.002 | 0.134 | 0.017 | UBC8 |
| 25 | TaUBC46 | TaUBC68 | 0.000 | 0.235 | 0.000 | UBC8 |
| 26 | TaUBC46 | TaUBC74 | 0.002 | 0.211 | 0.011 | UBC8 |
| 27 | TaUBC51 | TaUBC54 | 0.002 | 0.077 | 0.030 | UBC8 |
| 28 | TaUBC68 | TaUBC74 | 0.002 | 0.113 | 0.020 | UBC8 |
| 29 | TaUBC80 | TaUBC92 | 0.142 | 0.893 | 0.160 | UBC10 |
| 30 | TaUBC58 | TaUBC66 | 0.005 | 0.095 | 0.050 | UBC12 |
| 31 | TaUBC58 | TaUBC73 | 0.000 | 0.104 | 0.000 | UBC12 |
| 32 | TaUBC66 | TaUBC73 | 0.005 | 0.113 | 0.042 | UBC12 |
| 33 | TaUBC75 | TaUBC81 | 0.000 | 0.123 | 0.000 | UBC12 |
| 34 | TaUBC52 | TaUBC55 | 0.051 | 0.770 | 0.066 | UBC14 |
| 35 | TaUBC55 | TaUBC61 | 0.000 | 0.037 | 0.000 | UBC14 |
| 36 | TaUBC55 | TaUBC69 | 0.000 | 0.056 | 0.000 | UBC14 |
| 37 | TaUBC61 | TaUBC69 | 0.000 | 0.018 | 0.000 | UBC14 |
| 38 | TaUBC4 | TaUBC7 | 0.012 | 0.044 | 0.271 | UBC15 |
| 39 | TaUBC3 | TaUBC29 | 0.275 | 1.104 | 0.249 | UBC15 |
| 40 | TaUBC3 | TaUBC43 | 0.274 | 1.152 | 0.238 | UBC15 |
| 41 | TaUBC17 | TaUBC23 | 0.040 | 0.103 | 0.383 | UBC15 |
| 42 | TaUBC29 | TaUBC37 | 0.010 | 0.047 | 0.210 | UBC15 |
| 43 | TaUBC25 | TaUBC32 | 0.005 | 0.062 | 0.078 | UBC15 |
| 44 | TaUBC26 | TaUBC33 | 0.012 | 0.043 | 0.275 | UBC15 |
| 45 | TaUBC29 | TaUBC43 | 0.008 | 0.027 | 0.301 | UBC15 |
| 46 | TaUBC25 | TaUBC40 | 0.007 | 0.037 | 0.180 | UBC15 |
| 47 | TaUBC37 | TaUBC43 | 0.007 | 0.041 | 0.159 | UBC15 |
| 48 | TaUBC32 | TaUBC40 | 0.010 | 0.055 | 0.175 | UBC15 |
| 49 | TaUBC31 | TaUBC38 | 0.107 | 0.300 | 0.356 | UBC15 |
| 50 | TaUBC64 | TaUBC71 | 0.000 | 0.050 | 0.000 | UEV |
| 51 | TaUBC62 | TaUBC70 | 0.000 | 0.052 | 0.000 | UEV |

**Note:** Ks: Synonymous substitution; Ka: Non-synonymous substitution rate. Ka/Ks>1, positive selection; Ka/Ks=1, neutral evolution; Ka/Ks<1, negative purifying selection

**Table S3.** Analysis of *cis*-elements in the promoter of *TaUBC* genes.

| ***Cis*-element** | **Number of genes** | **Sequence** | **Response of type** |
| --- | --- | --- | --- |
| ARE | 71 | AAACCA | Stress |
| AT-rich sequence | 3 | TAAAATACT |  |
| box S | 28 | AGCCACC |  |
| CCAAT-box | 45 | CAACGG/CCCCCG |  |
| GC-motif | 56 | AGCGCGCCG |  |
| LTR | 49 | CCGAAA |  |
| MBS | 44 | CAACTG |  |
| MYB | 84 | CAACCA/TAACCA |  |
| MYB-like sequence | 44 | TAACCA |  |
| STRE | 85 | AGGGG |  |
| TCA | 24 | TCATCTTCAT/GTTTTCTTAC |  |
| TC-rich repeats | 18 | ATTCTCTAAC |  |
| W box | 40 | TTGACC |  |
| WRE3 | 59 | CCACCT |  |
| WUN-motif | 18 | AAATTTCCT |  |
| ABRE | 81 | ACGTG/AACCCGG | Hormone |
| ABRE3a | 28 | TACGTG |  |
| ABRE4 | 28 | CACGTA |  |
| AuxRR-core | 16 | GGTCCAT |  |
| CARE | 11 | CAACTCAC |  |
| CGTCA-motif | 83 | CGTCA |  |
| ERE | 33 | ATTTCATA |  |
| GARE-motif | 23 | TCTGTTG |  |
| P-box | 25 | CCTTTTG/CAACAAACCCCTT |  |
| TATC-box | 8 | TATCCCA |  |
| TCA-element | 30 | CCATCTTTTT/TCAGAAGAGG |  |
| TGA-element | 1 | TGACGTAA |  |
| TGACG-motif | 82 | TGACG |  |
| AC-I | 12 | (T/C)C(T/C)(C/T)ACC(T/C)ACC | Development |
| as-1 | 82 | TGACG |  |
| CAT-box | 46 | GCCACT |  |
| CCGTCC motif | 50 | CCGTCC |  |
| CCGTCC-box | 51 | CCGTCC |  |
| circadian | 11 | CAAAGATATC |  |
| dOCT | 7 | CTCGGATC/CACGGATC |  |
| GCN4_motif | 8 | TGAGTCA |  |
| MYB recognition site | 45 | CCGTTG |  |
| Myb-binding site | 60 | CAACAG |  |
| re2f-1 | 2 | GCGGGAAA |  |

**Table S4.** Primer sequences used in this study.

| Gene | Forward primer (5′–3′) | Reverse primer (5′–3′) |
| --- | --- | --- |
| *TaUBC6* | CCCAACAGTCCTCCATCAGT | TGCTGGAGACTCATCGTTTG |
| *TaUBC9* | GAAGAGGAAAGTGCCTTGACG | TGTTGGGGTGATAGATGGGAG |
| *TaUBC11* | CCACCCAAACATCAACAGCAAT | GTACATGTGAGCAATCTCAGGC |
| *TaUBC21* | GGAGCTGCTGGACCTCAAC | CGTTGCAGTGGTAAATCCTTG |
| *TaUBC25* | AGGGTAACATGGTCGGATCG | TCTTCAAACAGCGTCTTGAGGT |
| *TaUBC43* | GATTACTCCGACCACCACTATGC | CCAACAATGGCAGCCCTAA |
| *TaUBC78* | AGGCAGCCAACTCAAATGGA | AAGTGAATGACCCGCCTAGA |
| *TaUBC79* | TGAGGAGGATTGTCAAGGAGC | AAGTGGATGGTCACCAGGAAC |
| *TaUBC83* | CTCCTGTCCGTCGTCTCCA | GCTCATCGAACGTCTCCGTC |
| *TaUBC84* | GGAGCTGAATTTGCTCTGGC | TGGGGTACTTCTTGGGGATC |
| *TaUBC86* | CCCCCAGACTACCCATTCAAA | AGAGAGCAGATTGAAAGGAGCA |
| *TaActin3/TaAct* | GCCGTGCTTTCCCTCTATG | GCTTCTCCTTGATGTCCCTTA |
| *TaActin1* | AAATCTGGCATCACACTTTCTAC | GTCTCAAACATAATCTGGGTCATC |
| *Tubulin* | ACCGTGGTGATGTTGTGC | TGGTGGCTGGTAGTTGATA |

| **Table S5.** Variation sites and genotypes of *TaUBC25* gene in 681 wheat materials. | | | | | | | | | | | | | |
| --- | --- | --- | --- | --- | --- | --- | --- | --- | --- | --- | --- | --- | --- |
| No. | CHROM POS REF/ALT | chr3A 151312704 T/C | chr3A 151312740 C/A | chr3A 151312965 T/C | chr3A 151313199 C/A | chr3A 151313351 A/G | chr3A 151313498 T/C | chr3A 151313504 C/T | chr3A 151313687 T/C | chr3A 151315079 T/C | chr3A 151315116 C/T | chr3A 151318591 C/T | chr3A 151318711 T/C |
| 1 | Zang1817 | T/T | A/A | T/T | C/C | A/A |  |  | C/C | T/T | C/C | C/C | T/T |
| 2 | TW001 | T/T | C/C | T/T |  |  | T/T | C/C |  | T/T | C/C |  | T/T |
| 3 | TW002 | T/T | C/C | T/T | C/C | A/A | T/T | C/C | C/C | T/T | C/C |  | T/T |
| 4 | TW003 | T/T | C/C | T/T |  | A/A | T/T | C/C | C/C |  |  | C/C | T/T |
| 5 | TW004 | T/T |  | T/T | C/C | A/A | T/T | C/C |  |  |  | C/C | T/T |
| 6 | TW005 |  |  | T/T |  | A/A | T/T | C/C |  | T/T | C/C |  |  |
| 7 | TW006 | T/T |  | T/T | C/C | A/A | T/T | C/C |  | T/T | C/C |  | T/T |
| 8 | TW007 | C/C | C/C | C/C |  |  | C/C | T/T |  | C/C | T/T | T/T |  |
| 9 | TW008 | T/T | C/C |  | C/C | A/A |  |  | C/C | T/T | C/C | C/C |  |
| 10 | TW009 | T/T | C/C | T/T | C/C |  | T/T | C/C | C/C | T/T | C/C | C/C | T/T |
| 11 | TW010 | T/T | C/C | T/T | C/C |  | T/C | C/T | C/C | T/T |  | C/C |  |
| 12 | TW011 | C/C | C/C |  | A/A |  |  |  |  | C/C |  |  |  |
| 13 | TW012 |  |  |  |  |  |  |  | C/C | C/C | T/T | T/T |  |
| 14 | TW013 | C/C |  | C/C |  |  | C/C | T/T |  |  |  |  | T/C |
| 15 | TW014 |  |  |  |  |  |  |  |  | C/C |  | T/T | C/C |
| 16 | TW015 | C/C | C/C | C/C |  | G/G | C/C | T/T |  | C/C | T/T | T/T | C/C |
| 17 | TW016 | C/C | C/C |  |  |  |  |  | C/C | C/C |  | T/T | T/C |
| 18 | TW017 |  |  |  | C/C | G/G | C/C | T/T |  | C/C | T/T | T/T | C/C |
| 19 | TW018 | C/C | C/C | C/C |  |  | C/C | T/T |  |  |  | T/T |  |
| 20 | TW019 | C/C |  |  |  |  |  |  |  |  |  | T/T | C/C |
| 21 | TW020 |  |  | C/C |  | G/G | C/C | T/T | C/C | C/C | T/T | T/T | C/C |
| 22 | TW021 | C/C | C/C | C/C | A/A | G/G |  |  | C/C |  |  |  |  |
| 23 | TW022 | C/C |  |  | A/A | G/G | C/C | T/T |  |  |  |  | C/C |
| 24 | TW023 | C/C | C/C |  |  | G/G |  |  | C/C |  |  | C/T | C/C |
| 25 | TW024 | C/C | C/C | C/C | A/A | G/G | C/C | T/T | C/C |  |  |  |  |
| 26 | TW025 | T/T | C/C |  |  | A/A | T/T | C/C | T/T | T/T | C/C |  | T/T |
| 27 | TW026 |  |  | T/T |  | A/A |  |  |  |  |  | C/C |  |
| 28 | TW027 | T/T | C/C | T/T |  | A/A |  |  |  | T/T | C/C |  | T/T |
| 29 | TW028 | T/T | C/C | T/T | C/C | A/A | T/T | C/C | T/T | T/T | C/C | C/C | T/T |
| 30 | TW029 | T/T | C/C | T/T |  | A/A | T/T | C/C | T/T |  |  | C/C | T/T |
| 31 | TW030 |  | C/C |  |  | A/A |  |  | T/T | T/T | C/C | C/C |  |
| 32 | TW031 |  |  | T/T |  | A/A | T/T | C/C | T/T | T/T | C/C |  |  |
| 33 | TW032 | T/T | C/C |  | C/C | A/A | T/T | C/C | T/T | T/T |  | C/C | T/T |
| 34 | TW033 | T/T | C/C | T/T |  | A/A | T/T | C/C | T/T | T/T | C/C | C/C | T/T |
| 35 | TW034 |  |  |  |  |  |  |  |  |  |  | C/C |  |
| 36 | TW035 | T/T | C/C |  | C/C | A/A | T/T | C/C | C/C | T/T |  |  |  |
| 37 | TW036 |  |  | T/T | C/C | A/A | T/T | C/C |  | T/T | C/C |  |  |
| 38 | TW037 | T/T | C/C |  | C/C | A/A |  |  | C/C | T/T | C/C | C/C |  |
| 39 | TW038 |  |  |  |  |  |  |  |  |  |  |  |  |
| 40 | TW039 | T/T | C/C |  |  | A/A | T/T | C/C | C/C |  |  | C/C | T/T |
| 41 | TW040 | T/T |  |  |  |  |  | C/C | C/C |  |  |  | T/T |
| 42 | TW041 | T/T | C/C | T/T | C/C | A/A | T/T | C/C | C/C |  |  |  |  |
| 43 | TW043 | T/T | C/C |  |  |  |  |  |  | T/T | C/C |  | T/T |
| 44 | TW044 | T/T | C/C | T/T |  | A/A | T/T | C/C | C/C | T/T | C/C | C/C | T/T |
| 45 | TW045 | T/T | C/C |  |  |  | T/T | C/C |  | T/T | C/C | C/C | T/T |
| 46 | TW046 | T/T | C/C |  | C/C |  |  | C/C | C/C | T/T |  | C/C |  |
| 47 | TW047 | T/T | C/C |  | C/C |  |  |  |  | T/T | C/C | C/C |  |
| 48 | TW048 | T/T | C/C |  | C/C | A/A |  |  | C/C |  |  |  |  |
| 49 | TW049 | T/T | C/C | T/T | C/C | A/A | T/T | C/C | C/C | T/T | C/C | C/C | T/T |
| 50 | TW050 | T/T | A/A | T/T |  |  |  |  |  |  | C/C | C/C | T/T |
| 51 | TW051 | T/T | C/C | T/T | C/C |  |  | C/C |  | T/T | C/C | C/C | T/T |
| 52 | TW052 | T/T | C/C | T/T | C/C | A/A | T/T | C/C | C/C | T/T | C/C |  | T/T |
| 53 | TW053 |  |  | T/T | C/C | A/A | T/T | C/C | C/C |  |  | C/C |  |
| 54 | TW054 | T/T | C/C | T/T | C/C | A/A |  |  | C/C | T/T | C/C | C/C | T/T |
| 55 | AFG-L1 | T/T | C/C | T/T |  | A/A | T/T | C/C | C/C | T/T | C/C | C/C | T/T |
| 56 | AFG-L2 | T/T | A/A | T/T |  |  |  |  |  | T/T | C/C |  |  |
| 57 | AFG-L3 |  |  |  |  | A/A | T/T | C/C | C/C |  |  |  |  |
| 58 | AMN | T/T | C/C | T/T |  |  |  |  | C/C | T/T | C/C | C/C | T/T |
| 59 | AUT-L1 |  | C/C | T/T |  |  |  |  |  | T/T | C/C | C/C | T/T |
| 60 | BDHHM | T/T | C/C | T/T |  |  |  |  |  | T/T | C/C | C/C | T/T |
| 61 | BIH-L1 | T/T | C/C | T/T |  | A/A |  |  |  | T/T | C/C | C/C | T/T |
| 62 | BJBH |  |  |  |  | A/A | T/T | C/C |  |  |  |  |  |
| 63 | BMZ | T/T | A/A |  | C/C | A/A | T/T | C/C | C/C |  | C/C | C/C | T/T |
| 64 | BQM |  | C/C |  |  |  |  |  |  | T/T | C/C | C/C | T/T |
| 65 | BRA-L1 | T/T | C/C | T/T | C/C | A/A | T/T | C/C | C/C | T/T | C/C | C/C | T/T |
| 66 | BYM | T/T | C/C | T/T | C/C | A/A | T/T | C/C | T/C | T/T | C/C | C/C | T/T |
| 67 | CAN-C1 |  |  |  |  |  |  |  | C/C | T/T |  |  |  |
| 68 | CHE-L1 | T/T | C/C |  | C/C |  |  |  |  |  |  |  |  |
| 69 | CHL-C1 |  | C/C |  |  |  |  |  |  | T/T | C/C | C/C | T/T |
| 70 | CSB | T/T | C/C | T/T | C/C | A/A | T/T | C/C | C/C | T/T | C/C | C/C | T/T |
| 71 | CYHM |  | C/C | T/T | C/C | A/A |  |  |  |  |  | C/C | T/T |
| 72 | CZ6406 | T/T | C/C |  |  |  |  |  |  | T/T | C/C | C/C | T/T |
| 73 | DEU-L1 | T/T | C/C | T/T | C/C | A/A | T/T | C/C | C/C | T/T | C/C | C/C | T/T |
| 74 | DHM | T/T | A/A | T/T |  |  | T/T | C/C | C/C | T/T | C/C | C/C | T/T |
| 75 | DKM | T/T | C/C | T/T | C/C | A/A | T/T | C/C |  | T/T | C/C | C/C | T/T |
| 76 | DXHKYM | T/T | C/C | T/T | C/C | A/A | T/T | C/C | T/T |  | C/C |  |  |
| 77 | DYH | T/T | C/C | T/T | C/C | A/A | T/T | C/C |  | T/T | C/C | C/C | T/T |
| 78 | ESP-L1 |  |  | T/T | C/C | A/A | T/T | C/C | C/C | T/T | C/C | C/C | T/T |
| 79 | FIN-L1 | T/T | C/C |  |  | A/A | T/T | C/C | C/C | T/T | C/C | C/C | T/T |
| 80 | FMSY |  |  | T/T | C/C | A/A | T/T | C/C | C/C | T/T |  |  |  |
| 81 | FRA-C1 |  |  | T/T | C/C | A/A | T/T | C/C |  | T/T | C/C |  | T/T |
| 82 | GBR-L1 |  | C/C |  |  |  |  |  |  |  |  | C/C | T/T |
| 83 | GEO-L1 | T/T | C/C | T/T | C/C |  | T/T | C/C |  | T/T | C/C | C/C | T/T |
| 84 | GRC-L1 | T/T | C/C | T/T |  | A/A | T/T | C/C |  | T/T | C/C | C/C | T/T |
| 85 | GYWLL | T/T | A/A | T/T |  |  | T/T | C/C | C/C | T/T | C/C | C/C | T/T |
| 86 | HCM | T/T | C/C | T/T |  |  | T/T | C/C | T/T | T/T | C/C |  | T/T |
| 87 | HLM |  |  |  |  |  |  |  | C/C |  |  |  | T/T |
| 88 | HMM | T/T | C/C | T/T | C/C | A/A |  |  | C/C | T/T | C/C | C/C | T/T |
| 89 | HM | T/T | C/C | T/T | C/C |  |  |  | T/T | T/T | C/C | C/C | T/T |
| 90 | HNSH |  |  |  |  |  |  |  | C/C | T/T | C/C |  |  |
| 91 | HRV-L1 | T/T | C/C | T/T |  | A/A |  |  |  | T/T | C/C | C/C | T/T |
| 92 | HTZ |  |  | T/T | C/C | A/A | T/T | C/C | C/C |  |  | C/C | T/T |
| 93 | HUN-L1 | T/T | C/C | T/T | C/C | A/A | T/T | C/C | C/C | T/T | C/C | C/C | T/T |
| 94 | HZB |  |  | T/T | C/C | A/A |  |  | T/T |  |  |  |  |
| 95 | IND-L1 | T/T | C/C | T/T |  | A/A | T/T | C/C | C/C | T/T | C/C | C/C | T/T |
| 96 | IND-L2 | T/T | C/C | T/T | C/C | A/A | T/T | C/C | C/C | T/T | C/C | C/C | T/T |
| 97 | IRN-L1 | T/T | C/C |  |  |  |  |  |  |  |  | C/C | T/T |
| 98 | IRN-L2 |  |  |  |  |  |  |  |  | T/T | C/C |  |  |
| 99 | IRN-L3 | T/T | C/C | T/T | C/C | A/A | T/T | C/C | C/C | T/T | C/C | C/C | T/T |
| 100 | IRN-L4 | T/T | C/C | T/T | C/C | A/A | T/T | C/C | C/C | T/T | C/C | C/C | T/T |
| 101 | IRQ-L1 | T/T | C/C | T/T |  |  |  |  |  | T/T | C/C |  |  |
| 102 | IRQ-L2 | T/T | C/C | T/T |  |  |  |  |  | T/T | C/C | C/C | T/T |
| 103 | IRQ-L3 | T/T | C/C | T/T | C/C |  | T/T | C/C |  | T/T | C/C | C/C | T/T |
| 104 | IRQ-L4 | T/T | C/C |  |  |  |  |  |  | T/T | C/C | C/C | T/T |
| 105 | ITA-C1 | T/T | C/C | T/T | C/C | A/A |  |  | C/C | T/T | C/C | C/C | T/T |
| 106 | ITA-L1 | T/T | A/A | T/T | C/C | A/A | T/T | C/C |  | T/T | C/C | C/C | T/T |
| 107 | ITA-L2 |  |  | T/T | C/C | A/A | T/T | C/C | C/C | T/T | C/C | C/C | T/T |
| 108 | JCYLYL | T/T | C/C | T/T |  | A/A | T/T | C/C | C/C | T/T | C/C | C/C | T/T |
| 109 | JiaHM | T/T | C/C | T/T | C/C | A/A | T/T | C/C | T/T | T/T | C/C | C/C | T/T |
| 110 | JinHM | T/T | C/C | T/T |  | A/A | T/T | C/C |  | T/T | C/C | C/C | T/T |
| 111 | JM | T/T | C/C | T/T |  | A/A |  | C/C | T/T | T/T | C/C | C/C | T/T |
| 112 | JM8H | T/T | C/C | T/T |  | A/A |  |  |  | T/T | C/C | C/C | T/T |
| 113 | JPN-L1 | T/T | C/C | T/T | C/C | A/A | T/T | C/C |  | T/T | C/C | C/C | T/T |
| 114 | KAZ-L1 | T/T | C/C | T/T |  | A/A | T/T | C/C |  | T/T | C/C | C/C | T/T |
| 115 | KFEH | T/T | C/C | T/T | C/C | A/A | T/T | C/C | C/C | T/T | C/C | C/C | T/T |
| 116 | KFSH | T/T |  | T/T | C/C | A/A | T/T | C/C | C/C | T/T | C/C |  | T/T |
| 117 | KSBP | T/T | C/C | T/T | C/C | A/A | T/T | C/C | C/C | T/T | C/C | C/C | T/T |
| 118 | KXSH | T/T | C/A | T/T | C/C | A/A | T/T | C/C | T/C | T/T | C/C | C/C | T/T |
| 119 | LGD | T/T | C/C | T/T |  |  |  |  |  | T/T | C/C |  | T/T |
| 120 | LLX | T/T |  |  |  |  |  |  |  | T/T | C/C |  |  |
| 121 | LM | T/T | C/C | T/T | C/C | A/A | T/T | C/C | C/C | T/T | C/C | C/C | T/T |
| 122 | LTT | T/T | C/C | T/T | C/C | A/A | T/T | C/C | T/T | T/T | C/C | C/C | T/T |
| 123 | LXZXM | T/T | C/C | T/T |  | A/A | T/T | C/C | T/T |  | C/C | C/C | T/T |
| 124 | LZT | T/T | C/C | T/T | C/C | A/A |  |  |  | T/T | C/C | C/C |  |
| 125 | MEX-L1 | T/T | C/C | T/T | C/C | A/A |  |  | C/C | T/T | C/C | C/C | T/T |
| 126 | MKD-L1 | T/T | C/A | T/T | C/C | A/A |  |  |  | T/T | C/C | C/C | T/T |
| 127 | MXM | T/T | C/C | T/T | C/C |  |  |  |  | T/T | C/C | C/C | T/T |
| 128 | NLD-C1 | T/T | C/C | T/T | C/C | A/A |  |  |  |  |  | C/C | T/T |
| 129 | NLD-L1 | T/T |  | T/T | C/C | A/A | T/T | C/C | C/C | T/T | C/C | C/C | T/T |
| 130 | NXWH | T/T | C/A | T/T |  | A/A | T/T | C/C | T/C | T/T | C/C | C/C | T/T |
| 131 | PAK-L1 | T/T | C/C | T/T | C/C | A/A |  |  |  | T/T | C/C | C/C | T/T |
| 132 | PAK-L2 | T/T | C/C | T/T | C/C |  | T/T | C/C |  | T/T | C/C | C/C | T/T |
| 133 | PAK-L3 | T/T | C/C | T/T | C/C | A/A | T/T | C/C | T/T | T/T | C/C | C/C | T/T |
| 134 | PRT-L1 | T/T | C/C | T/T |  | A/A | T/T | C/C |  | T/T | C/C | C/C | T/T |
| 135 | PYEQ | T/T | C/C | T/T |  | A/A | T/T | C/C | C/C | T/T | C/C | C/C | T/T |
| 136 | QCM | T/T | C/C | T/T | C/C | A/A |  |  |  | T/T | C/C | C/C | T/T |
| 137 | ROM-L1 | T/T | C/C | T/T | C/C | A/A | T/T | C/C | T/T | T/T | C/C | C/C | T/T |
| 138 | RUS-L1 | T/T | C/C | T/T |  |  |  |  |  | T/T | C/C | C/C | T/T |
| 139 | SLZ | T/T | C/C | T/T |  |  | T/T |  | T/T | T/T | C/C | C/C |  |
| 140 | SM | T/T | C/C |  | C/C |  |  |  |  |  |  | C/C | T/T |
| 141 | SRB-L1 | T/T | C/C | T/T | C/C | A/A |  |  |  | T/T | C/C | C/C | T/T |
| 142 | SRM4H | T/T | C/C | T/T | C/C | A/A | T/T | C/C | T/T | T/T | C/C | C/C | T/T |
| 143 | SWE-L1 | T/T | C/C | T/T |  |  |  |  |  | T/T | C/C |  | T/T |
| 144 | SYR-L1 |  |  |  |  |  |  |  |  |  |  |  |  |
| 145 | SYR-L2 | T/T | C/C | T/T |  |  |  |  |  |  |  |  |  |
| 146 | TJBXM | T/T | C/C |  | C/C | A/A | T/T | C/C | T/T | T/T | C/C | C/C | T/T |
| 147 | TJK-L1 | T/T | C/C | T/T | C/C |  |  |  | C/C | T/T | C/C | C/C | T/T |
| 148 | TMM |  |  | T/T | C/C | A/A | T/T | C/C | T/T |  |  | C/C | T/T |
| 149 | TUR-L1 | T/T | C/C | T/T |  |  |  |  |  | T/T | C/C | C/C | T/T |
| 150 | TUR-L2 | T/T | C/C | T/T | C/C | A/A | T/T | C/C | C/C |  |  | C/C | T/T |
| 151 | TUR-L3 | T/T |  | T/T |  |  |  |  |  |  |  |  |  |
| 152 | TUR-L4 | T/T | C/C | T/T | C/C | A/A | T/T | C/C |  | T/T | C/C | C/C | T/T |
| 153 | UKR-L1 | T/T | C/C | T/T | C/C | A/A |  |  | C/C |  | C/C |  | T/T |
| 154 | UKR-L2 | T/T | C/A | T/T | C/C | A/A | T/T | C/C | C/C |  | C/C | C/C | T/T |
| 155 | URY-L1 | T/T | C/C | T/T |  |  | T/T | C/C |  |  | C/C | C/C | T/T |
| 156 | URY-L2 | T/T | C/C | T/T | C/C |  | T/T | C/C | C/C | T/T | C/C | C/C |  |
| 157 | USA-C1 | T/T | C/C |  |  | A/A | T/T | C/C | C/C | T/T | C/C | C/C | T/T |
| 158 | UZB-C1 | T/T | C/C | T/T | C/C | A/A | T/T | C/C | T/T | T/T | C/C | C/C | T/T |
| 159 | UZB-L1 | T/T | C/C | T/T | C/C |  |  |  |  | T/T | C/C | C/C | T/T |
| 160 | WYM | T/T | C/C | T/T | C/C | A/A |  |  | T/T | T/T | C/C | C/C | T/T |
| 161 | XFS | T/T | C/C | T/T |  |  |  |  |  | T/T | C/C | C/C | T/T |
| 162 | XHP | T/T | C/C | T/T | C/C | A/A |  |  | T/T | T/T | C/C |  | T/T |
| 163 | XYLH | T/T | C/C | T/T |  | A/A |  | C/C | C/C | T/T | C/C | C/C | T/T |
| 164 | YASY | T/T | C/C | T/T | C/C |  | T/T | C/C |  | T/T | C/C | C/C | T/T |
| 165 | YMBF | T/T |  | T/T | C/C | A/A | T/T | C/C | T/T | T/T | C/C |  | T/T |
| 166 | YM | T/T | C/C | T/T | C/C | A/A | T/T | C/C | T/T | T/T | C/C |  | T/T |
| 167 | YNSW | T/T | C/C |  | C/C |  |  |  | C/C |  |  | C/C | T/T |
| 168 | YZM | T/T | C/C | T/T |  | A/A | T/T | C/C | C/C | T/T |  | C/C | T/T |
| 169 | ZDSH | T/T | C/C | T/T | C/C | A/A | T/T | C/C | C/C | T/T | C/C | C/C | T/T |
| 170 | ZJH | T/T | C/C | T/T |  |  | T/T | C/C | C/C | T/T | C/C | C/C | T/T |
| 171 | ZP | T/T | C/C | T/T | C/C |  | T/T |  | T/T | T/T | C/C | C/C | T/T |
| 172 | ZWT | T/T | C/C | T/T |  |  |  |  |  | T/T | C/C | C/C | T/T |
| 173 | ZYJWLQ | T/T | C/C |  | C/C | A/A | T/T | C/C |  | T/T | C/C | C/C | T/T |
| 174 | MP001 | T/T | A/A | T/T | C/C | A/A | T/T |  |  | T/T | C/C | C/C | T/T |
| 175 | MP002 | T/T | C/C | T/T | C/C | A/A | T/T | C/C | C/C | T/T | C/C | C/C | T/T |
| 176 | MP003 | T/T | C/C |  | C/C | A/A | T/T | C/C | C/C | T/T | C/C | C/C | T/T |
| 177 | MP004 | C/C | C/C | C/C | A/A |  | C/C | T/T | C/C | C/C | T/T | T/T | C/C |
| 178 | MP005 | T/T | A/A | T/T | C/C | A/A | T/T | C/C | C/C |  |  | C/C | T/T |
| 179 | MP006 | T/T | C/C | T/T | C/C | A/A | T/T | C/C | C/C | T/T | C/C | C/C | T/T |
| 180 | MP007 |  |  | T/T | C/C |  | T/T | C/C | C/C | T/T | C/C | C/C | T/T |
| 181 | MP008 | T/T | C/C |  | C/C | A/A | T/T | C/C | C/C |  |  | C/C | T/T |
| 182 | MP009 | T/T | C/C | T/T | C/C | A/A | T/T | C/C | C/C | T/T | C/C | C/C | T/T |
| 183 | MP010 | T/T | C/C | T/T | C/C | A/A | T/T | C/C | C/C | T/T | C/C | C/C | T/T |
| 184 | MP011 | T/T | C/C | T/T | C/C | A/A | T/T | C/C | C/C | T/T | C/C | C/C | T/T |
| 185 | MP012 | T/T | C/C | T/T | C/C | A/A | T/T | C/C | C/C | T/T | C/C | C/C | T/T |
| 186 | MP013 | T/T | C/C | T/T |  | A/A | T/T | C/C | C/C | T/T |  | C/C | T/T |
| 187 | MP014 | T/T | C/C | T/T | C/C | A/A | T/T | C/C |  | T/T | C/C | C/C | T/T |
| 188 | MP015 | T/T | C/C | T/T | C/C | A/A | T/T | C/C | C/C | T/T | C/C | C/C | T/T |
| 189 | MP016 | T/T | C/C | T/T | C/C | A/A | T/T | C/C |  | T/T | C/C | C/C | T/T |
| 190 | MP017 | T/T | C/C | T/T | C/C | A/A | T/T | C/C | C/C |  |  | C/C | T/T |
| 191 | MP018 | T/T | C/C | T/T | C/C |  |  |  |  | T/T | C/C | C/C | T/T |
| 192 | MP019 |  |  | T/T | C/C |  |  |  | C/C | T/T | C/C | C/C | T/T |
| 193 | MP020 | T/T | C/C | T/T | C/C |  | T/T | C/C | C/C |  |  | C/C | T/T |
| 194 | MP021 | T/T | C/C | T/T | C/C | A/A | T/T | C/C | T/T | T/T | C/C | C/C | T/T |
| 195 | MP022 | T/T | C/C | T/T | C/C | A/A | T/T | C/C | T/T | T/T | C/C | C/C | T/T |
| 196 | MP023 | T/T | C/C | T/T | C/C | A/A | T/T | C/C | C/C | T/T | C/C | C/C | T/T |
| 197 | MP024 | T/T | C/C | T/T | C/C | A/A | T/T | C/C | T/T | T/T | C/C | C/C | T/T |
| 198 | MP025 | T/T | C/C |  |  | A/A |  | C/C | T/T | T/T | C/C | C/C | T/T |
| 199 | MP026 | T/T | C/C | T/T | C/C | A/A | T/T | C/C | T/T | T/T | C/C | C/C | T/T |
| 200 | MP027 | T/T | C/C | T/T |  | A/A | T/T | C/C | T/T | T/T | C/C | C/C | T/T |
| 201 | MP028 | T/T | C/C |  | C/C | A/A | T/T | C/C | C/C | T/T | C/C | C/C | T/T |
| 202 | MP029 | T/T | C/C | T/T | C/C | A/A | T/T | C/C | T/T | T/T | C/C | C/C | T/T |
| 203 | MP030 | T/T | C/C | T/T | C/C | A/A |  |  |  | T/T | C/C | C/C | T/T |
| 204 | MP031 | T/T |  |  | C/C | A/A | T/T | C/C |  | T/T | C/C | C/C |  |
| 205 | MP032 | T/T | C/C | T/T | C/C |  |  |  |  | T/T | C/C | C/C | T/T |
| 206 | MP033 | C/C |  | C/C | A/A |  |  |  | C/C | C/C | T/T | T/T | C/C |
| 207 | MP034 | T/T | C/C | T/T | C/C |  |  |  | C/C | T/T | C/C | C/C | T/T |
| 208 | MP035 |  |  | T/T | C/C | A/A |  |  | T/T | T/T | C/C | C/C | T/T |
| 209 | MP036 | T/T | C/C | T/T | C/C |  | T/T | C/C |  | T/T | C/C | C/C | T/T |
| 210 | MP037 | T/T | C/C | T/T | C/C |  |  |  | C/C | T/T | C/C | C/C | T/T |
| 211 | MP038 | T/T | C/C | T/T | C/C | A/A |  |  |  | T/T | C/C | C/C | T/T |
| 212 | MP039 | T/T | C/C | T/T | C/C |  |  |  | C/C | T/T | C/C | C/C | T/T |
| 213 | MP040 | T/T | C/C | T/T | C/C | A/A |  |  |  | T/T | C/C | C/C | T/T |
| 214 | MP041 | T/T | C/C | T/T | C/C | A/A | T/T | C/C | T/T | T/T | C/C | C/C | T/T |
| 215 | MP042 | T/T | A/A | T/T | C/C |  | T/T | C/C | C/C |  |  | C/C | T/T |
| 216 | MP043 | T/T |  | T/T | C/C |  |  |  |  | T/T | C/C | C/C | T/T |
| 217 | MP044 | T/T | C/C | T/T | C/C | A/A | T/T | C/C | T/T | T/T | C/C | C/C | T/T |
| 218 | MP045 | T/T | C/C | T/T | C/C | A/A | T/T | C/C | T/T | T/T | C/C | C/C | T/T |
| 219 | MP046 | T/T | C/C | T/T | C/C | A/A | T/T | C/C | T/T | T/T | C/C | C/C | T/T |
| 220 | MP047 | T/T | C/C | T/T | C/C | A/A | T/T | C/C | T/T | T/T | C/C | C/C | T/T |
| 221 | MP048 |  |  | T/T | C/C | A/A |  |  | C/C | T/T | C/C | C/C | T/T |
| 222 | MP049 | T/T | C/C | T/T | C/C | A/A |  |  | T/T | T/T | C/C | C/C | T/T |
| 223 | MP050 | T/T | C/C | T/T | C/C | A/A | T/T | C/C | C/C | T/T | C/C | C/C | T/T |
| 224 | MP051 | T/T | C/C | T/T | C/C | A/A | T/T | C/C | T/T | T/T | C/C | C/C | T/T |
| 225 | MP052 | T/T | C/C | T/T |  | A/A | T/T | C/C |  |  | C/C | C/C | T/T |
| 226 | MP053 | T/T | C/C | T/T | C/C | A/A | T/T | C/C | C/C | T/T | C/C | C/C | T/T |
| 227 | MP054 | T/T | C/C | T/T | C/C | A/A | T/T | C/C | C/C |  |  | C/C | T/T |
| 228 | MP055 |  |  | T/T | C/C |  | T/T | C/C | C/C | T/T | C/C | C/C | T/T |
| 229 | MP056 |  |  | T/T | C/C | A/A | T/T | C/C | C/C | T/T | C/C | C/C | T/T |
| 230 | MP057 | T/T | C/C | T/T | C/C |  | T/T | C/C | C/C | T/T | C/C | C/C | T/T |
| 231 | MP058 |  |  |  | C/C | A/A | T/T | C/C | T/T | T/T | C/C | C/C | T/T |
| 232 | MP059 | T/T | C/C | T/T | C/C | A/A | T/T | C/C | C/C | T/T | C/C | C/C | T/T |
| 233 | MP060 |  |  |  | C/C | A/A | T/T | C/C | C/C | T/T | C/C |  |  |
| 234 | MP061 | T/T | C/C | T/T | C/C | A/A | T/T |  | C/C | T/T | C/C | C/C |  |
| 235 | MP062 | T/T | C/C | T/T |  | A/A | T/T | C/C | C/C | T/T | C/C | C/C | T/T |
| 236 | MP063 | T/T | C/C |  |  | A/A | T/T | C/C | C/C | T/T | C/C | C/C | T/T |
| 237 | MP064 | T/T | C/C | T/T | C/C | A/A | T/T | C/C | C/C | T/T | C/C | C/C | T/T |
| 238 | MP065 |  | C/C | T/T | C/C | A/A | T/T | C/C | C/C | T/T | C/C | C/C | T/T |
| 239 | MP066 | T/T | C/C | T/T | C/C |  | T/T | C/C | C/C | T/T | C/C | C/C | T/T |
| 240 | MP067 | T/T | C/C | T/T | C/C | A/A | T/T | C/C | C/C | T/T | C/C | C/C | T/T |
| 241 | MP068 | T/T | C/C | T/T | C/C | A/A | T/T | C/C | C/C | T/T |  | C/C | T/T |
| 242 | MP069 | T/T | C/C | T/T | C/C | A/G | T/T | C/C | C/C | T/T | C/C | C/C | T/T |
| 243 | MP070 | T/T | C/C | T/T | C/C | A/A | T/T | C/C | C/C | T/T | C/C | C/C | T/T |
| 244 | MP071 | T/T | C/C | T/T | C/C | A/A | T/T | C/C | C/C | T/T | C/C |  | T/T |
| 245 | MP072 | T/T | C/C | T/T | C/C | A/A | T/T | C/C | C/C |  |  |  | T/T |
| 246 | MP073 | T/T | C/C | T/T | C/C | A/A | T/T | C/C | C/C | T/T | C/C | C/C | T/T |
| 247 | MP074 |  | C/C | T/T | C/C |  | T/T | C/C | C/C |  | C/C | C/C | T/T |
| 248 | MP075 |  |  | T/T | C/C | A/A | T/T | C/C | C/C | T/T |  | C/C | T/T |
| 249 | MP076 | T/T | C/C | T/T |  |  | T/T | C/C | C/C | T/T | C/C | C/C | T/T |
| 250 | MP077 | T/T | C/C | T/T | C/C | A/A | T/T | C/C | C/C | T/T | C/C | C/C | T/T |
| 251 | MP078 |  |  |  |  |  |  |  |  |  |  |  |  |
| 252 | MP079 | T/T | C/C | T/T | C/C | A/A | T/T | C/C | C/C | T/T | C/C | C/C | T/T |
| 253 | MP080 | T/T | C/C | T/T | C/C | A/A | T/T | C/C |  | T/T | C/C |  | T/T |
| 254 | MP081 | T/T | C/C | T/T | C/C | A/A |  |  | C/C | T/T | C/C | C/C | T/T |
| 255 | MP082 |  | C/C | T/T |  | A/A |  |  | C/C |  |  | C/C | T/T |
| 256 | MP083 | T/T | C/C | T/T |  | A/A | T/T | C/C | C/C | T/T | C/C | C/C | T/T |
| 257 | MP084 | T/T | C/C | T/T | C/C | A/A | T/T | C/C |  |  |  | C/C | T/T |
| 258 | MP085 | T/T | C/C | T/T | C/C | A/A |  |  | C/C |  |  | C/C | T/T |
| 259 | MP086 | T/T | C/C | T/T | C/C | A/A | T/T | C/C | C/C | T/T | C/C | C/C | T/T |
| 260 | MP087 | T/T | C/C | T/T |  | A/A | T/T | C/C | C/C | T/T |  | C/C | T/T |
| 261 | MP088 | T/T | C/C |  | C/C | A/A | T/T | C/C | C/C | T/T | C/C | C/C | T/T |
| 262 | MP089 | T/T | C/C | T/T | C/C | A/A | T/T | C/C | C/C | T/T | C/C | C/C | T/T |
| 263 | MP090 | T/T |  | T/T | C/C | A/A | T/T | C/C | C/C | T/T | C/C | C/C | T/T |
| 264 | MP091 | T/T | C/C | T/T | C/C | A/A |  |  | C/C | T/T | C/C | C/C | T/T |
| 265 | MP092 | T/T | C/C | T/T |  | A/A | T/T | C/C | C/C | T/T | C/C | C/C | T/T |
| 266 | MP093 | T/T | C/C | T/T | C/C | A/A | T/T | C/C | C/C | T/T | C/C | C/C | T/T |
| 267 | MP094 | T/T | C/C | T/T | C/C | A/A | T/T | C/C | C/C | T/T | C/C | C/C | T/T |
| 268 | MP095 | T/T | C/C | T/T | C/C | A/A | T/T | C/C | C/C | T/T | C/C | C/C | T/T |
| 269 | MP096 | T/T | C/C | T/T | C/C | A/A | T/T | C/C | C/C | T/T | C/C | C/C | T/T |
| 270 | MP097 | T/T | A/A | T/T |  |  | T/T | C/C | C/C |  | C/C | C/C | T/T |
| 271 | MP098 | T/T | C/C | T/T | C/C |  | T/T | C/C | C/C | T/T | C/C | C/C | T/T |
| 272 | MP099 | T/T | C/C | T/T |  | A/A | T/T | C/C | C/C | T/T | C/C | C/C | T/T |
| 273 | MP100 | T/T | C/C | T/T | C/C | A/A | T/T | C/C | C/C | T/T | C/C | C/C | T/T |
| 274 | MP101 | T/T | C/C | T/T | C/C | A/A | T/T | C/C | T/C | T/T | C/C | C/C | T/T |
| 275 | MP102 | T/T | C/C | T/T | C/C | A/A | T/T | C/C | C/C | T/T | C/C | C/C | T/T |
| 276 | MP103 | T/T | A/A | T/T | C/C | A/A | T/T | C/C | C/C | T/T | C/C | C/C | T/T |
| 277 | MP104 | T/T | C/C | T/T | C/C | A/A | T/T | C/C | C/C | T/T | C/C | C/C | T/T |
| 278 | MP105 | T/T | C/C |  | C/C |  |  |  |  | T/T | C/C | C/C | T/T |
| 279 | MP106 | T/T | C/C | T/T | C/C | A/A | T/T | C/C | C/C | T/T | C/C | C/C | T/T |
| 280 | MP107 | T/T | C/C | T/T | C/C | A/A | T/T | C/C |  | T/T | C/C | C/C | T/T |
| 281 | MP108 | T/T | C/C | T/T | C/C | A/A | T/T | C/C | C/C | T/T | C/C | C/C | T/T |
| 282 | MP109 | T/T | C/C | T/T |  | A/A | T/T | C/C | C/C | T/T | C/C |  | T/T |
| 283 | MP110 | T/T | C/C | T/T | C/C | A/A | T/T | C/C | C/C | T/T | C/C | C/C | T/T |
| 284 | MP111 | T/T | C/C | T/T | C/C |  |  |  | C/C | T/T | C/C | C/C | T/T |
| 285 | MP112 | T/T | C/C | T/T | C/C |  | T/T | C/C | C/C | T/T | C/C | C/C | T/T |
| 286 | MP113 | T/T | C/C | T/T | C/C | A/A | T/T | C/C | C/C | T/T | C/C | C/C | T/T |
| 287 | MP114 | T/T | C/C | T/T | C/C | A/A | T/T | C/C | C/C | T/T | C/C | C/C |  |
| 288 | MP115 | T/T | C/C | T/T | C/C | A/A | T/T | C/C | C/C | T/T | C/C | C/C | T/T |
| 289 | MP116 |  |  |  |  | A/A |  | C/C | C/C |  |  |  | T/T |
| 290 | MP117 | T/T | C/C | T/T | C/C | A/A | T/T | C/C | C/C | T/T | C/C | C/C | T/T |
| 291 | MP118 | T/T | C/C | T/T | C/C | A/A |  |  | C/C | T/T | C/C | C/C | T/T |
| 292 | MP119 | T/T | C/C | T/T | C/C | A/A | T/T | C/C | C/C | T/T | C/C | C/C | T/T |
| 293 | MP120 | T/T | C/C | T/T | C/C | A/A | T/T | C/C | C/C | T/T | C/C | C/C | T/T |
| 294 | MP121 |  |  |  |  | A/A |  |  | C/C | T/T | C/C | C/C | T/T |
| 295 | MP122 | T/T | C/C | T/T |  | A/A | T/T | C/C |  | T/T | C/C | C/C | T/T |
| 296 | MP123 | T/T | C/C | T/T | C/C | A/A |  | C/C |  | T/T | C/C | C/C | T/T |
| 297 | MP124 | T/T | C/C |  |  |  | T/T | C/C | C/C | T/T | C/C | C/C | T/T |
| 298 | MP125 | T/T | C/C | T/T | C/C | A/A | T/T | C/C | C/C | T/T | C/C | C/C | T/T |
| 299 | MP126 | T/T | C/C | T/T | C/C | A/A | T/T | C/C | C/C | T/T | C/C | C/C | T/T |
| 300 | MP127 | T/T | C/C | T/T | C/C | A/A | T/T | C/C | C/C | T/T | C/C | C/C | T/T |
| 301 | MP128 | T/T | C/C | T/T | C/C | A/A | T/T | C/C | C/C | T/T | C/C | C/C | T/T |
| 302 | MP129 | T/T | C/C | T/T | C/C | A/A | T/T | C/C | C/C | T/T | C/C | C/C | T/T |
| 303 | MP130 |  | C/C | T/T |  | A/A | T/T | C/C | C/C | T/T | C/C | C/C | T/T |
| 304 | MP131 | T/T | C/C | T/T | C/C | A/A |  |  | C/C | T/T | C/C | C/C | T/T |
| 305 | MP132 | T/T | C/C | T/T |  | A/A | T/T | C/C | C/C |  |  | C/C |  |
| 306 | MP133 | T/T | C/C | T/T |  |  |  |  | C/C | T/T | C/C | C/C | T/T |
| 307 | MP134 | T/T | C/C | T/T | C/C | A/A | T/T | C/C | C/C | T/T | C/C |  | T/T |
| 308 | MP135 | T/T | C/C | T/T | C/C | A/A | T/T | C/C | C/C | T/T | C/C | C/C | T/T |
| 309 | MP136 | T/T | C/C | T/T | C/C | A/A | T/T | C/C | C/C | T/T | C/C | C/C | T/T |
| 310 | MP137 | T/T | C/C | T/T | C/C | A/A | T/T | C/C | C/C | T/T | C/C | C/C | T/T |
| 311 | MP138 | T/T | C/C | T/T | C/C | A/A |  |  | C/C | T/T | C/C | C/C |  |
| 312 | MP139 | T/T | C/C |  | C/C | A/A | T/T | C/C | C/C | T/T | C/C | C/C | T/T |
| 313 | MP140 | T/T | C/C | T/T |  | A/A |  |  |  | T/T | C/C | C/C | T/T |
| 314 | MP141 | T/T |  | T/T | C/C | A/A | T/T | C/C | C/C | T/T | C/C | C/C | T/T |
| 315 | MP142 | T/T | C/C | T/T | C/C | A/A | T/T | C/C | C/C | T/T | C/C | C/C | T/T |
| 316 | MP143 | T/T | C/C | T/T | C/C | A/A | T/T | C/C | C/C | T/T | C/C |  | T/T |
| 317 | MP144 | T/T | C/C | T/T | C/C | A/A | T/T | C/C | C/C | T/T | C/C | C/C | T/T |
| 318 | MP145 | T/T | C/C | T/T |  | A/A | T/T |  | C/C | T/T | C/C | C/C | T/T |
| 319 | S1 | T/T | C/C | T/T |  | A/A | T/T | C/C | C/C | T/T | C/C | C/C | T/T |
| 320 | S2 | T/T | C/C | T/T | C/C | A/A | T/T | C/C | C/C | T/T | C/C | C/C | T/T |
| 321 | S3 | T/T | C/C | T/T | C/C | A/A | T/T | C/C | T/T | T/T | C/C | C/C | T/T |
| 322 | S4 | T/T | C/C | T/T | C/C | A/A | T/T | C/C | C/C | T/T | C/C | C/C | T/T |
| 323 | S5 | T/T | C/C | T/T | C/C | A/A | T/T | C/C | C/C | T/T | C/C | C/C | T/T |
| 324 | S6 | T/T | C/C | T/T | C/C | A/A | T/T | C/C | T/T | T/T | C/C | C/C | T/T |
| 325 | S7 | C/C | C/C | C/C | A/A | G/G | C/C | T/T | C/C | C/C | T/T | T/T | C/C |
| 326 | S8 | C/C | C/C | C/C | A/A | G/G | C/C | T/T | C/C | C/C | T/T | T/T | C/C |
| 327 | S9 | T/T | C/C | T/T | C/C | A/A | T/T | C/C | C/C | T/T | C/C | C/C | T/T |
| 328 | S10 | T/T | C/C | T/T | C/C | A/A | T/T | C/C | C/C | T/T | C/C | C/C | T/T |
| 329 | S11 | T/T | C/C | T/T | C/C | A/A | T/T | C/C | T/T | T/T | C/C | C/C | T/T |
| 330 | S12 | T/T | C/C | T/T | C/C | A/A | T/T | C/C | C/C | T/T | C/C | C/C | T/T |
| 331 | S13 | T/T | A/A | T/T | C/C | A/A | T/T | C/C | C/C | T/T | C/C | C/C | T/T |
| 332 | S14 | T/T | C/C | T/T | C/C | A/A | T/T | C/C | C/C | T/T |  | C/C | T/T |
| 333 | S15 | T/T | C/C | T/T | C/C | A/A | T/T | C/C | C/C | T/T | C/C | C/C | T/T |
| 334 | S16 | T/T | C/C | T/T | C/C | A/A | T/T | C/C | T/T | T/T | C/C | C/C | T/T |
| 335 | S17 | T/T | C/C | T/T | C/C | A/A | T/T | C/C | C/C | T/T | C/C | C/C | T/T |
| 336 | S18 | T/T | C/C | T/T | C/C |  | T/T | C/C | C/C | T/T | C/C | C/C | T/T |
| 337 | S19 | T/T | C/C | T/T | C/C | A/A | T/T | C/C | C/C | T/T | C/C |  | T/T |
| 338 | S20 | T/T | C/C | T/T | C/C | A/A | T/T | C/C | C/C | T/T | C/C | C/C | T/T |
| 339 | S21 | T/T | C/C | T/T | C/C | A/A | T/T | C/C | C/C | T/T | C/C | C/C | T/T |
| 340 | S22 | T/T | C/C | T/T | C/C | A/A | T/T | C/C | C/C | T/T | C/C | C/C | T/T |
| 341 | S23 | T/T | C/C | T/T | C/C | A/A | T/T | C/C | T/T | T/T | C/C | C/C | T/T |
| 342 | S24 | T/T | C/C | T/T | C/C | A/A | T/T | C/C | T/T | T/T | C/C | C/C | T/T |
| 343 | S25 | T/T | C/C | T/T | C/C | A/A | T/T | C/C | C/C | T/T | C/C | C/C | T/T |
| 344 | S26 | T/T |  | T/T | C/C | A/A | T/T | C/C | C/C | T/T | C/C | C/C | T/T |
| 345 | S27 | T/T | C/C | T/T | C/C | A/A | T/T | C/C | C/C |  |  | C/C | T/T |
| 346 | S28 | T/T | C/C | T/T | C/C | A/A | T/T | C/C | C/C | T/T | C/C | C/C |  |
| 347 | S29 | T/T | C/C | T/T |  | A/A | T/T | C/C | C/C | T/T | C/C | C/C | T/T |
| 348 | S30 | T/T | C/C | T/T | C/C | A/A | T/T | C/C | C/C | T/T | C/C | C/C | T/T |
| 349 | S31 | T/T | C/C | T/T | C/C | A/A | T/T | C/C | T/T | T/T | C/C | C/C | T/T |
| 350 | S32 | T/T | C/C | T/T | C/C |  | T/T | C/C | C/C | T/T | C/C | C/C | T/T |
| 351 | S33 | T/T | C/C | T/T | C/C | A/A | T/T | C/C | C/C | T/T | C/C | C/C | T/T |
| 352 | S34 | T/T | C/C | T/T |  |  |  |  | C/C | T/T | C/C | C/C | T/T |
| 353 | S35 | T/T | C/C | T/T | C/C | A/A | T/T | C/C | C/C | T/T | C/C | C/C | T/T |
| 354 | S36 | T/T | C/C |  | C/C | A/A | T/T | C/C | C/C | T/T | C/C | C/C | T/T |
| 355 | S37 | T/T | C/C | T/T | C/C | A/A |  | C/C | C/C | T/T | C/C | C/C | T/T |
| 356 | S38 | T/T | C/C | T/T | C/C | A/A | T/T | C/C | C/C | T/T | C/C | C/C | T/T |
| 357 | S39 | T/T | C/C | T/T | C/C | A/A |  |  | C/C | T/T | C/C |  | T/T |
| 358 | S40 | T/T | C/C | T/T | C/C |  |  |  | T/T | T/T | C/C | C/C | T/T |
| 359 | S41 | T/T | C/C |  | C/C | A/A | T/T | C/C | C/C | T/T | C/C | C/C | T/T |
| 360 | S42 | T/T | C/C | T/T | C/C | A/A | T/T | C/C | C/C | T/T | C/C | C/C | T/T |
| 361 | S43 | T/T | C/C | T/T | C/C | A/A | T/T | C/C | C/C | T/T | C/C | C/C | T/T |
| 362 | S44 | T/T | C/C | T/T | C/C | A/A | T/T | C/C | C/C | T/T | C/C | C/C | T/T |
| 363 | S45 | T/T | C/C | T/T | C/C |  | T/T | C/C | C/C | T/T | C/C | C/C | T/T |
| 364 | S46 | T/T | C/C | T/T | C/C | A/A | T/T | C/C | C/C | T/T | C/C | C/C | T/T |
| 365 | S47 | T/T | C/C | T/T | C/C | A/A | T/T | C/C | C/C | T/T | C/C | C/C | T/T |
| 366 | S48 | T/T | C/C | T/T |  | A/A | T/T | C/C | C/C | T/T |  | C/C | T/T |
| 367 | S49 | T/T | C/C | T/T | C/C | A/A | T/T | C/C | C/C |  | C/C | C/C | T/T |
| 368 | S50 | T/T | C/C | T/T | C/C | A/A | T/T | C/C | C/C | T/T | C/C | C/C | T/T |
| 369 | S51 | T/T | C/C | T/T | C/C | A/A | T/T | C/C | C/C | T/T | C/C |  |  |
| 370 | S52 | T/T | C/C | T/T | C/C | A/A | T/T | C/C | T/T |  | C/C | C/C | T/T |
| 371 | S56 | T/T | C/C | T/T | C/C | A/A | T/T | C/C | T/T | T/T | C/C | C/C | T/T |
| 372 | S57 | T/T | C/C | T/T | C/C | A/A | T/T | C/C | T/T | T/T | C/C | C/C | T/T |
| 373 | S58 | T/T | C/C | T/T | C/C | A/A | T/T | C/C | C/C | T/T | C/C | C/C | T/T |
| 374 | S59 | T/T | C/C | T/T | C/C | A/A | T/T | C/C | C/C | T/T | C/C | C/C | T/T |
| 375 | S60 | T/T | C/C | T/T | C/C |  |  | C/C | C/C | T/T | C/C | C/C | T/T |
| 376 | S61 | T/T | C/C | T/T | C/C | A/A | T/T | C/C | T/T | T/T | C/C |  | T/T |
| 377 | S62 |  | C/C |  | C/C | A/A | T/T | C/C | C/C | T/T | C/C |  | T/T |
| 378 | S64 | T/T |  |  | C/C | A/A | T/T | C/C | C/C | T/T | C/C | C/C | T/T |
| 379 | S67 |  | C/C | T/T | C/C | A/A |  |  | C/C | T/T | C/C | C/C | T/T |
| 380 | S72 | T/T | C/C | T/T | C/C | A/A | T/T | C/C | C/C | T/T | C/C | C/C | T/T |
| 381 | S73 | T/T | C/C | T/T | C/C | A/A | T/T | C/C | C/C | T/T | C/C | C/C | T/T |
| 382 | S74 | T/T | C/C | T/T |  | A/A | T/T | C/C | C/C |  | C/C | C/C | T/T |
| 383 | S75 | T/T | C/C | T/T |  | A/A | T/T | C/C | C/C | T/T | C/C | C/C | T/T |
| 384 | S76 | T/T | C/C | T/T |  | A/A |  |  |  | T/T | C/C | C/C |  |
| 385 | S77 | T/T | C/C | T/T | C/C | A/A | T/T | C/C | C/C | T/T | C/C | C/C | T/T |
| 386 | S78 | T/T | C/C | T/T | C/C | A/A | T/T | C/C | C/C | T/T | C/C | C/C | T/T |
| 387 | S79 | T/T | C/C | T/T | C/C | A/A | T/T | C/C | C/C | T/T | C/C | C/C | T/T |
| 388 | S80 | T/T | C/C | T/T | C/C | A/A | T/T | C/C |  | T/T | C/C | C/C | T/T |
| 389 | S81 | T/T | C/C | T/T | C/C | A/A | T/T | C/C | T/T | T/T | C/C | C/C | T/T |
| 390 | S82 | T/T | C/C | T/T | C/C | A/A | T/T | C/C | C/C | T/T | C/C | C/C | T/T |
| 391 | S83 | T/T | C/C | T/T | C/C | A/A | T/T | C/C | C/C | T/T | C/C | C/C | T/T |
| 392 | S84 | T/T | C/C | T/T | C/C | A/A | T/T | C/C | C/C | T/T | C/C |  |  |
| 393 | S86 | T/T | C/C | T/T | C/C | A/A | T/T | C/C | C/C | T/T | C/C | C/C | T/T |
| 394 | S87 | T/T | C/C | T/T | C/C | A/A | T/T | C/C | C/C | T/T | C/C | C/C | T/T |
| 395 | S88 | T/T | C/C | T/T | C/C | A/A | T/T | C/C |  | T/T | C/C | C/C | T/T |
| 396 | S89 | T/T | C/C | T/T | C/C | A/A | T/T | C/C | T/T | T/T | C/C | C/C | T/T |
| 397 | S90 | T/T | C/C | T/T |  | A/A | T/T | C/C | C/C | T/T | C/C | C/C | T/T |
| 398 | S91 | T/T | C/C | T/T | C/C | A/A | T/T | C/C | C/C | T/T | C/C | C/C | T/T |
| 399 | S92 | T/T | C/C | T/T | C/C | A/A | T/T | C/C | C/C | T/T | C/C | C/C | T/T |
| 400 | S93 | T/T | C/C | T/T | C/C | A/A | T/T | C/C |  | T/T | C/C |  | T/T |
| 401 | S94 | T/T | C/C | T/T | C/C | A/A | T/T | C/C | T/T | T/T | C/C | C/C | T/T |
| 402 | S95 | T/T | C/C | T/T | C/C | A/A | T/T | C/C | T/T | T/T | C/C | C/C | T/T |
| 403 | S96 | T/T | C/C |  | C/C | A/A | T/T | C/C | T/T | T/T | C/C | C/C | T/T |
| 404 | S97 | T/T | C/C | T/T |  | A/A | T/T | C/C | T/T | T/T | C/C | C/C | T/T |
| 405 | S98 | T/T | C/C |  | C/C | A/A | T/T | C/C | T/T | T/T | C/C | C/C | T/T |
| 406 | S99 | T/T | C/C | T/T | C/C | A/A | T/T | C/C | T/T | T/T | C/C | C/C | T/T |
| 407 | S100 | T/T | C/C | T/T | C/C | A/A | T/T | C/C | T/T | T/T | C/C | C/C | T/T |
| 408 | S101 | T/T | C/C | T/T | C/C | A/A | T/T | C/C | T/T | T/T | C/C | C/C | T/T |
| 409 | S102 | T/T | C/C | T/T | C/C | A/A | T/T | C/C | T/T | T/T | C/C | C/C | T/T |
| 410 | S103 | T/T | C/C | T/T | C/C | A/A | T/T | C/C | C/C | T/T | C/C | C/C | T/T |
| 411 | S104 | T/T | C/C | T/T |  | A/A | T/T | C/C | C/C | T/T | C/C | C/C | T/T |
| 412 | S105 | T/T | C/C | T/T | C/C | A/A | T/T | C/C | T/T | T/T | C/C | C/C | T/T |
| 413 | S106 | T/T | C/C | T/T | C/C | A/A | T/T | C/C | C/C | T/T | C/C | C/C | T/T |
| 414 | S108 |  |  |  | C/C | A/A | T/T | C/C | T/T |  |  |  | T/T |
| 415 | S109 | T/T | C/C | T/T | C/C | A/A | T/T | C/C |  | T/T | C/C | C/C | T/T |
| 416 | S110 | T/T | C/C | T/T | C/C | A/A | T/T | C/C |  | T/T | C/C | C/C | T/T |
| 417 | S111 | T/T | C/C | T/T |  | A/A | T/T | C/C | T/T | T/T | C/C | C/C | T/T |
| 418 | S112 | T/T | C/C | T/T | C/C | A/A | T/T | C/C | T/C | T/T | C/C | C/C | T/T |
| 419 | S113 | T/T | C/C | T/T | C/C | A/A | T/T | C/C | T/T | T/T | C/C | C/C | T/T |
| 420 | S114 | T/T |  | T/T | C/C | A/A | T/T | C/C | T/T | T/T | C/C | C/C | T/T |
| 421 | S115 |  | C/C | C/C | A/A | G/G | C/C | T/T | C/C | C/C | T/T | T/T | C/C |
| 422 | S116 | C/C | C/C | C/C | A/A | G/G | C/C | T/T | C/C | C/C | T/T | T/T | C/C |
| 423 | S117 | C/C | C/C | C/C |  | G/G | C/C | T/T | C/C | C/C | T/T |  | C/C |
| 424 | S118 | C/C | C/C | C/C | A/A | G/G | C/C | T/T |  | C/C | T/T | T/T | C/C |
| 425 | S119 | C/C | C/C | C/C | A/A | G/G | C/C | T/T | C/C | C/C | T/T | T/T | C/C |
| 426 | S120 | C/C | C/C | C/C | A/A | G/G | C/C | T/T | C/C | C/C | T/T | T/T | C/C |
| 427 | S121 | C/C | C/C | C/C | A/A | G/G | C/C | T/T | C/C | C/C | T/T | T/T | C/C |
| 428 | S122 | T/T | C/C | T/T | C/C | A/A | T/T | C/C | C/C | T/T | C/C | C/C | T/T |
| 429 | S123 |  |  | T/T | C/C | A/A | T/T | C/C | C/C | T/T | C/C | C/C | T/T |
| 430 | S124 | T/T | C/C | T/T | C/C | A/A | T/T | C/C | C/C | T/T | C/C | C/C | T/T |
| 431 | S125 | T/T | C/C | T/T | C/C | A/A | T/T | C/C | C/C | T/T | C/C | C/C | T/T |
| 432 | S126 | T/T | C/C | T/T | C/C | A/A | T/T | C/C | C/C | T/T | C/C | C/C |  |
| 433 | S127 | T/T | C/C |  | C/C | A/A | T/T | C/C |  |  |  | C/C | T/T |
| 434 | S128 | T/T | C/C | T/T | C/C | A/A | T/T | C/C | C/C |  | C/C | C/C |  |
| 435 | S129 | T/T | C/C | T/T | C/C | A/A | T/T | C/C | C/C | T/T | C/C |  | T/T |
| 436 | S130 | T/T | C/C | T/T | C/C | A/A | T/T | C/C | C/C | T/T | C/C | C/C | T/T |
| 437 | S131 | T/T | C/C | T/T | C/C | A/A | T/T | C/C | C/C | T/T | C/C | C/C | T/T |
| 438 | S132 |  |  |  |  | A/A | T/T | C/C | C/C | T/T | C/C | C/C | T/T |
| 439 | S133 | T/T | C/C | T/T | C/C | A/A |  |  | C/C | T/T | C/C | C/C | T/T |
| 440 | S134 | T/T | C/C | T/T | C/C | A/A | T/T | C/C | C/C | T/T | C/C | C/C | T/T |
| 441 | S135 |  |  | T/T | C/C | A/A | T/T | C/C |  |  |  | C/C | T/T |
| 442 | S136 | T/T | C/C | T/T | C/C | A/A |  |  | C/C | T/T | C/C |  | T/T |
| 443 | S137 | T/T | C/C | T/T | C/C | A/A | T/T | C/C | C/C | T/T | C/C | C/C | T/T |
| 444 | S139 | T/T | C/C | T/T | C/C | A/A | T/T | C/C | C/C |  | C/C | C/C | T/T |
| 445 | S140 | T/T | C/C | T/T |  | A/A | T/T | C/C | C/C |  | C/C | C/C | T/T |
| 446 | S142 | T/T | C/C | T/T | C/C | A/A | T/T | C/C | C/C | T/T | C/C | C/C | T/T |
| 447 | S143 | T/T | C/C | T/T | C/C | A/A | T/T | C/C | C/C | T/T | C/C | C/C | T/T |
| 448 | S144 | T/T | C/C | T/T | C/C | A/A |  |  | C/C | T/T | C/C | C/C | T/T |
| 449 | S145 | T/T |  | T/T | C/C | A/A | T/T | C/C | T/T | T/T | C/C | C/C | T/T |
| 450 | S146 | T/T | C/C | T/T | C/C | A/A | T/T | C/C | C/C | T/T | C/C | C/C | T/T |
| 451 | S147 | T/T | C/C | T/T | C/C | A/A | T/T | C/C | C/C | T/T | C/C | C/C | T/T |
| 452 | S148 | T/T | C/C | T/T | C/C | A/A | T/T | C/C | C/C | T/T | C/C | C/C | T/T |
| 453 | S149 | T/T | C/C |  | C/C | A/A | T/T | C/C | C/C |  |  | C/C | T/T |
| 454 | S150 | T/T | C/C | T/T | C/C | A/A | T/T | C/C | C/C | T/T | C/C | C/C | T/T |
| 455 | S151 | T/T | C/C | T/T | C/C | A/A |  |  | C/C |  |  | C/C | T/T |
| 456 | S152 | T/T | C/C | T/T | C/C |  |  |  | C/C | T/T | C/C | C/C | T/T |
| 457 | S153 | T/T | C/C | T/T | C/C | A/A | T/T | C/C | C/C |  |  | C/C | T/T |
| 458 | S154 | T/T | C/C | T/T | C/C | A/A | T/T | C/C | C/C | T/T | C/C | C/C | T/T |
| 459 | S155 | T/T | C/C | T/T | C/C | A/A | T/T | C/C | C/C | T/T | C/C | C/C | T/T |
| 460 | S156 | T/T | C/C | T/T | C/C | A/A | T/T | C/C | C/C | T/T | C/C | C/C | T/T |
| 461 | S157 | T/T | C/C | T/T | C/C | A/A | T/T | C/C | C/C | T/T | C/C | C/C | T/T |
| 462 | S158 | T/T | C/C | T/T | C/C | A/A | T/T | C/C | C/C |  | C/C |  | T/T |
| 463 | S159 | T/T | C/C | T/T | C/C | A/A | T/T | C/C | C/C | T/T | C/C | C/C | T/T |
| 464 | S160 | T/T | C/C | T/T | C/C | A/A | T/T | C/C | C/C | T/T | C/C | C/C | T/T |
| 465 | S161 | T/T | C/C | T/T | C/C | A/A | T/T | C/C | C/C | T/T | C/C | C/C | T/T |
| 466 | S162 | T/T | C/C | T/T | C/C | A/A | T/T | C/C | T/T | T/T | C/C |  | T/T |
| 467 | S163 | T/T | C/C | T/T | C/C | A/A | T/T | C/C | C/C | T/T | C/C | C/C | T/T |
| 468 | S164 | T/T | C/C | T/T | C/C | A/A | T/T | C/C | C/C | T/T | C/C | C/C | T/T |
| 469 | S165 | T/T | C/C | T/T | C/C | A/A | T/T | C/C | C/C | T/T | C/C | C/C | T/T |
| 470 | S166 | T/T | C/C | T/T | C/C | A/A | T/T | C/C | C/C | T/T | C/C | C/C | T/T |
| 471 | S167 | T/T | C/C | T/T | C/C | A/A | T/T | C/C | C/C |  |  | C/C | T/T |
| 472 | S168 | T/T | C/C | T/T |  |  | T/T | C/C | C/C |  |  | C/C | T/T |
| 473 | S169 | T/T | C/C | T/T | C/C | A/A | T/T | C/C | C/C | T/T | C/C | C/C | T/T |
| 474 | S170 | T/T | C/C | T/T |  | A/A | T/T | C/C | T/T | T/T | C/C | C/C | T/T |
| 475 | S171 | T/T | C/C | T/T | C/C | A/A | T/T | C/C | C/C | T/T | C/C | C/C | T/T |
| 476 | S172 | T/T | C/C | T/T | C/C | A/A | T/T | C/C | C/C | T/T | C/C | C/C | T/T |
| 477 | S173 | T/T | C/C | T/T | C/C | A/A | T/T | C/C | C/C | T/T | C/C | C/C | T/T |
| 478 | S174 | T/T | C/C |  | C/C | A/A | T/T | C/C | C/C | T/T | C/C |  | T/T |
| 479 | S175 | T/T | A/A | T/T | C/C | A/A | T/T | C/C | T/C | T/T | C/C |  | T/T |
| 480 | S176 | T/T | C/C | T/T | C/C | A/A | T/T | C/C | T/T | T/T | C/C | C/C | T/T |
| 481 | S177 | T/T |  | T/T | C/C | A/A | T/T | C/C | T/T | T/T | C/C | C/C | T/T |
| 482 | S178 | T/T | C/C | T/T | C/C | A/A | T/T | C/C | T/T | T/T | C/C | C/C | T/T |
| 483 | S179 | T/T | C/C | T/T | C/C | A/A | T/T | C/C | T/T | T/T | C/C | C/C | T/T |
| 484 | S180 | T/T | C/C | T/T | C/C | A/A | T/T | C/C |  | T/T | C/C | C/C | T/T |
| 485 | S181 | T/T | C/C | T/T | C/C | A/A | T/T | C/C | T/T | T/T | C/C | C/C | T/T |
| 486 | S182 | T/T |  | T/T | C/C | A/A | T/T | C/C | T/T |  | C/C |  | T/T |
| 487 | S183 | T/T | C/C | T/T | C/C | A/A |  |  | T/T | T/T | C/C | C/C | T/T |
| 488 | S184 | T/T | C/C | T/T | C/C | A/A | T/T | C/C |  | T/T | C/C | C/C | T/T |
| 489 | S185 | T/T | C/C | T/T | C/C | A/A | T/T | C/C | T/C | T/T | C/C | C/C | T/T |
| 490 | S186 | T/T | C/C | T/T |  |  | T/T | C/C | T/T | T/T | C/C |  | T/T |
| 491 | S187 | T/T | C/C | T/T | C/C | A/A | T/T | C/C | C/C | T/T | C/C | C/C | T/T |
| 492 | S188 | T/T | C/C | T/T | C/C | A/A |  |  | C/C | T/T | C/C | C/C | T/T |
| 493 | S203 | T/T | A/A | T/T |  | A/A | T/T | C/C | C/C | T/T | C/C | C/C | T/T |
| 494 | S204 | T/T | C/C | T/T | C/C | A/A | T/T | C/C | C/C | T/T | C/C | C/C | T/T |
| 495 | S205 | T/T | C/C |  | C/C | A/A | T/T | C/C | T/T | T/T | C/C |  | T/T |
| 496 | S206 | T/T | C/C | T/T | C/C |  | T/T | C/C | T/T | T/T | C/C | C/C | T/T |
| 497 | S207 | T/T | C/C | T/T | C/C | A/A | T/T | C/C | C/C | T/T | C/C | C/C | T/T |
| 498 | S208 | T/T | C/C | T/T | C/C | A/A | T/T | C/C | C/C | T/T | C/C | C/C | T/T |
| 499 | S209 | T/T | C/C | T/T | C/C |  |  |  | C/C | T/T | C/C | C/C | T/T |
| 500 | S210 | T/T | C/C | T/T |  | A/A | T/T | C/C | C/C | T/T | C/C | C/C | T/T |
| 501 | S211 | T/T | C/C | T/T | C/C | A/A |  |  | C/C | T/T | C/C | C/C | T/T |
| 502 | S212 | T/T | C/C | T/T | C/C | A/A | T/T | C/C | C/C | T/T | C/C | C/C | T/T |
| 503 | S213 | T/T | C/C | T/T | C/C | A/A | T/T | C/C | T/T | T/T | C/C | C/C | T/T |
| 504 | S214 | T/T | C/C |  | C/C | A/A | T/T | C/C | T/T | T/T | C/C | C/C | T/T |
| 505 | S215 | T/T | C/C | T/T | C/C | A/A | T/T | C/C | C/C | T/T | C/C | C/C | T/T |
| 506 | S216 | T/T |  | T/T | C/C | A/A | T/T | C/C | C/C | T/T | C/C | C/C | T/T |
| 507 | S217 |  |  |  | C/C | A/A | T/T | C/C | C/C | T/T | C/C | C/C | T/T |
| 508 | S218 | T/T | C/C | T/T | C/C | A/A | T/T | C/C | C/C | T/T | C/C | C/C | T/T |
| 509 | S219 | T/T | C/C | T/T | C/C | A/A | T/T | C/C | C/C | T/T | C/C | C/C | T/T |
| 510 | S220 |  |  | T/T | C/C | A/A | T/T | C/C | C/C | T/T | C/C | C/C | T/T |
| 511 | S221 | T/T | C/C | T/T | C/C | A/A | T/T | C/C | C/C | T/T | C/C | C/C | T/T |
| 512 | S223 | T/T | C/C | T/T | C/C | A/A | T/T | C/C | C/C | T/T | C/C | C/C | T/T |
| 513 | S224 | T/T | C/C | T/T | C/C | A/A | T/T | C/C | C/C | T/T | C/C | C/C | T/T |
| 514 | S225 | T/T | C/C | T/T | C/C | A/A |  | C/C | C/C | T/T | C/C | C/C | T/T |
| 515 | S227 | T/T | C/C | T/T |  | A/A | T/T | C/C | C/C | T/T | C/C | C/C | T/T |
| 516 | S228 | T/T | C/C | T/T | C/C | A/A | T/T | C/C |  | T/T | C/C | C/C | T/T |
| 517 | S229 | T/T | C/C | T/T | C/C | A/A | T/T | C/C | C/C | T/T | C/C | C/C | T/T |
| 518 | S230 | T/T | C/C | T/T | C/C | A/A |  |  |  | T/T | C/C | C/C | T/T |
| 519 | S231 | T/T | C/C | T/T | C/C | A/A | T/T | C/C | C/C | T/T | C/C | C/C | T/T |
| 520 | S232 | C/C | C/C | C/C | A/A | G/G | C/C | T/T | C/C | C/C | T/T | T/T | C/C |
| 521 | S233 | T/T | C/C | T/T | C/C | A/A | T/T | C/C | C/C | T/T | C/C | C/C | T/T |
| 522 | S234 | T/T | A/A | T/T | C/C | A/A | T/T | C/C | C/C | T/T | C/C | C/C | T/T |
| 523 | S235 | T/T | C/C | T/T |  |  | T/T | C/C | C/C | T/T | C/C | C/C | T/T |
| 524 | S236 | T/T | C/C | T/T | C/C | A/A | T/T | C/C | C/C | T/T | C/C | C/C | T/T |
| 525 | S237 | T/T | C/C | T/T |  | A/A | T/T | C/C | C/C | T/T | C/C | C/C | T/T |
| 526 | S238 |  |  | T/T | C/C | A/A | T/T | C/C | C/C | T/T | C/C | C/C |  |
| 527 | S239 | T/T | C/C | T/T | C/C | A/A | T/T | C/C | C/C | T/T | C/C | C/C | T/T |
| 528 | S240 |  |  | T/T | C/C | A/A | T/T | C/C | T/T | T/T | C/C | C/C | T/T |
| 529 | S241 | T/T | C/C | T/T | C/C |  | T/T | C/C | T/T | T/T | C/C | C/C | T/T |
| 530 | S242 | T/T | C/C | T/T | C/C | A/A | T/T | C/C |  | T/T | C/C | C/C | T/T |
| 531 | S243 | T/T | C/C | T/T | C/C | A/A | T/T | C/C |  | T/T | C/C | C/C |  |
| 532 | S244 | T/T | C/C | T/T |  | A/A | T/T | C/C | T/T | T/T | C/C | C/C | T/T |
| 533 | S245 | T/T | C/C | T/T | C/C | A/A |  |  | C/C | T/T | C/C | C/C | T/T |
| 534 | S246 | T/T | C/C | T/T | C/C | A/A | T/T | C/C |  | T/T | C/C | C/C | T/T |
| 535 | S247 | T/T | C/C | T/T | C/C | A/A | T/T | C/C | C/C | T/T | C/C | C/C | T/T |
| 536 | S248 | T/T | C/C | T/T | C/C | A/A | T/T | C/C | T/T | T/T | C/C | C/C | T/T |
| 537 | S249 | T/T | C/C | T/T | C/C | A/A | T/T | C/C | T/T | T/T | C/C | C/C | T/T |
| 538 | S250 | T/T | C/C | T/T | C/C | A/A | T/T | C/C | T/T | T/T | C/C | C/C | T/T |
| 539 | S251 | T/T | C/C | T/T | C/C | A/A | T/T | C/C | T/T | T/T | C/C | C/C | T/T |
| 540 | S252 | T/T | C/C | T/T | C/C | A/A | T/T | C/C | C/C | T/T | C/C | C/C | T/T |
| 541 | S253 | T/T | C/C | T/T | C/C | A/A |  |  | C/C | T/T | C/C | C/C | T/T |
| 542 | S254 | T/T | C/C | T/T | C/C | A/A | T/T | C/C | C/C | T/T | C/C | C/C | T/T |
| 543 | S255 | T/T | C/C | T/T | C/C | A/A | T/T | C/C | T/T | T/T | C/C | C/C | T/T |
| 544 | S256 |  |  |  |  |  |  |  |  |  |  |  |  |
| 545 | S257 | T/T | C/C | T/T |  | A/A | T/T | C/C | T/T | T/T | C/C | C/C | T/T |
| 546 | S258 | T/T | C/C | T/T | C/C | A/A | T/T | C/C | C/C | T/T | C/C | C/C | T/T |
| 547 | S259 | T/T | C/C | T/T | C/C | A/A | T/T | C/C | T/T | T/T | C/C | C/C | T/T |
| 548 | S260 | T/T | C/C | T/T | C/C | A/A | T/T |  | C/C | T/T | C/C | C/C | T/T |
| 549 | S261 | T/T | C/C | T/T |  | A/A |  |  | C/C | T/T | C/C |  |  |
| 550 | S262 | T/T | C/C |  | C/C | A/A | T/T | C/C | T/T | T/T | C/C | C/C | T/T |
| 551 | S264 | T/T | C/C | T/T | C/C |  | T/T | C/C | C/C | T/T | C/C | C/C | T/T |
| 552 | S265 | T/T | C/C | T/T | C/C | A/A | T/T | C/C | C/C | T/T | C/C | C/C | T/T |
| 553 | S266 | T/T | C/C | T/T | C/C | A/A | T/T | C/C | C/C | T/T | C/C | C/C | T/T |
| 554 | S267 | T/T | C/C | T/T | C/C | A/A | T/T | C/C | C/C | T/T | C/C | C/C | T/T |
| 555 | S268 | T/T | C/C | T/T | C/C | A/A | T/T | C/C | C/C | T/T | C/C | C/C | T/T |
| 556 | S270 | T/T | C/C | T/T |  |  | T/T | C/C |  | T/T | C/C | C/C | T/T |
| 557 | S271 | T/T | C/C | T/T | C/C | A/A | T/T | C/C | C/C | T/T | C/C | C/C | T/T |
| 558 | S272 |  | C/C | T/T | C/C | A/A | T/T | C/C | C/C |  |  |  | T/T |
| 559 | S273 |  |  | T/T | C/C |  | T/T | C/C | C/C |  | C/C | C/C |  |
| 560 | S274 | T/T | C/C | T/T | C/C | A/A | T/T | C/C | C/C | T/T | C/C | C/C | T/T |
| 561 | S3331 | T/T | C/C | T/T | C/C |  | T/T | C/C |  | T/T | C/C | C/C | T/T |
| 562 | S6554 | T/T | C/C | T/T | C/C | A/A | T/T | C/C | C/C | T/T | C/C |  | T/T |
| 563 | CS | T/T | C/C | T/T | C/C | A/A |  |  | T/T | T/T | C/C | C/C | T/T |
| 564 | C1 |  | C/C | T/T |  |  | T/T | C/C | C/C |  | C/C |  |  |
| 565 | C2 | T/T | C/C |  | C/C | A/A | T/T | C/C | C/C |  |  | C/C | T/T |
| 566 | C3 | T/T | C/C | T/T | C/C | A/A | T/T | C/C |  | T/T | C/C | C/C | T/T |
| 567 | C4 |  |  | T/T | C/C |  | T/T | C/C | C/C | T/T | C/C | C/C |  |
| 568 | C5 | T/T | C/C | T/T |  |  |  | C/C | C/C | T/T | C/C | C/C | T/T |
| 569 | C6 | T/T | C/C | T/T | C/C | A/A | T/T | C/C | C/C |  |  | C/C | T/T |
| 570 | C7 | T/T | C/C |  | C/C | A/A | T/T | C/C | C/C | T/T |  | C/C | T/T |
| 571 | C8 | T/T | C/C | T/T |  |  |  |  | C/C |  | C/C | C/C | T/T |
| 572 | C9 | T/T | C/C | T/T | C/C | A/A | T/T | C/C | C/C | T/T | C/C | C/C | T/T |
| 573 | C10 |  |  | T/T | C/C | A/A | T/T | C/C | C/C | T/T | C/C |  |  |
| 574 | C11 | T/T | A/A | T/T |  | A/A | T/T | C/C |  |  |  |  | T/T |
| 575 | C12 | T/T |  | T/T | C/C | A/A | T/T | C/C | C/C | T/T | C/C | C/C | T/T |
| 576 | C13 | T/T | C/C |  | C/C | A/A | T/T | C/C | C/C |  |  | C/C | T/T |
| 577 | C14 | T/T | C/C | T/T | C/C | A/A | T/T | C/C |  | T/T | C/C |  |  |
| 578 | C15 |  |  | T/T | C/C | A/A | T/T | C/C | C/C | T/T | C/C | C/C | T/T |
| 579 | C16 | T/T | C/C | T/T |  |  |  |  | C/C | T/T | C/C | C/C | T/T |
| 580 | C17 | T/T | C/C | T/T | C/C | A/A | T/T | C/C | C/C | T/T | C/C | C/C | T/T |
| 581 | C18 | T/T |  | T/T | C/C | A/A | T/T | C/C | C/C | T/T | C/C | C/C | T/T |
| 582 | C19 |  |  | T/T | C/C | A/A | T/T | C/C | C/C | T/T | C/C | C/C | T/T |
| 583 | C20 | T/T | C/C | T/T | C/C | A/A | T/T | C/C | C/C | T/T | C/C | C/C | T/T |
| 584 | C21 |  | C/C | T/T | C/C |  | T/T | C/C | C/C | T/T |  | C/C |  |
| 585 | C22 | T/T | A/A | T/T | C/C | A/A |  | C/C |  | T/T | C/C | C/C | T/T |
| 586 | C23 | T/T | C/C | T/T | C/C |  | T/T | C/C | T/T | T/T | C/C | C/C | T/T |
| 587 | C24 | T/T | C/C | T/T | C/C | A/A | T/T | C/C | C/C | T/T | C/C | C/C | T/T |
| 588 | C25 | T/T | C/C | T/T | C/C | A/A | T/T | C/C | C/C |  |  |  | T/T |
| 589 | C26 |  |  | T/T | C/C | A/A | T/T | C/C | C/C | T/T | C/C |  | T/T |
| 590 | C27 |  | C/C |  | C/C | A/A | T/T | C/C |  | T/T | C/C |  | T/T |
| 591 | C28 | T/T | C/C | T/T | C/C | A/A | T/T | C/C | C/C | T/T | C/C | C/C | T/T |
| 592 | C29 | T/T | C/C | T/T |  | A/A | T/T | C/C | C/C | T/T | C/C | C/C | T/T |
| 593 | C30 | T/T | C/C | T/T |  |  | T/T | C/C |  |  |  | C/C | T/T |
| 594 | C31 | T/T | C/C | T/T | C/C | A/A | T/T | C/C | C/C |  | C/C | C/C | T/T |
| 595 | C32 |  |  | T/T | C/C | A/A | T/T | C/C | C/C |  |  | C/C | T/T |
| 596 | C33 | T/T | C/C | T/T | C/C | A/A | T/T | C/C | C/C | T/T |  | C/C | T/T |
| 597 | C34 | T/T | C/C | T/T | C/C | A/A | T/T | C/C |  |  |  | C/C | T/T |
| 598 | C35 | T/T |  | T/C |  | A/G | T/C | C/T | T/C |  |  |  |  |
| 599 | C36 | T/T | C/C | T/T | C/C | A/A | T/T | C/C | T/T | T/T | C/C | C/C |  |
| 600 | C37 | T/T | C/C | T/T | C/C | A/A | T/T | C/C | T/T | T/T | C/C | C/C | T/T |
| 601 | C38 | T/T | C/C | T/T | C/C | A/A | T/T | C/C | T/T | T/T | C/C | C/C | T/T |
| 602 | C39 | T/T | C/A | T/T | C/C | A/A | T/T | C/C |  | T/T | C/C | C/C | T/T |
| 603 | C40 | T/T | A/A | T/T | C/C | A/A | T/T | C/C | C/C | T/T | C/C | C/C | T/T |
| 604 | C41 | T/T | C/C | T/T | C/C | A/A | T/T | C/C | C/C | T/T | C/C | C/C |  |
| 605 | C42 | T/T | C/C | T/T | C/C | A/A | T/T | C/C | C/C | T/T | C/C | C/C | T/T |
| 606 | C43 | T/T | C/C | T/T | C/C | A/A | T/T | C/C | C/C | T/T | C/C | C/C | T/T |
| 607 | C44 |  |  | T/T | C/C | A/A | T/T | C/C | C/C |  |  | C/C | T/T |
| 608 | C45 | T/T | C/C | T/T | C/C | A/A | T/T | C/C | C/C | T/T | C/C | C/C | T/T |
| 609 | C46 |  | C/C | T/T | C/C | A/A | T/T | C/C | C/C | T/T | C/C | C/C | T/T |
| 610 | C47 | T/T | C/C | T/T | C/C | A/A | T/T | C/C | C/C | T/T | C/C | C/C | T/T |
| 611 | C48 |  |  |  | C/C | A/A | T/T | C/C | C/C | T/T | C/C |  | T/T |
| 612 | C49 | C/C | C/C | C/C | A/A | G/G | C/C | T/T | C/C | C/C | T/T | T/T | C/C |
| 613 | C50 | T/T | C/C | T/T | C/C | A/A | T/T | C/C | C/C | T/T | C/C | C/C | T/T |
| 614 | C51 | T/T | C/C | T/T | C/C | A/A | T/T | C/C | C/C | T/T | C/C | C/C | T/T |
| 615 | C52 | T/T | C/C | T/T | C/C | A/A | T/T | C/C | C/C | T/T | C/C | C/C | T/T |
| 616 | C53 | T/T | C/C | T/T | C/C | A/A | T/T | C/C | C/C | T/T | C/C | C/C | T/T |
| 617 | C54 | T/T | C/C | T/T |  |  | T/T | C/C | C/C | T/T | C/C | C/C | T/T |
| 618 | C55 | T/T | C/C | T/T | C/C | A/A | T/T | C/C | C/C | T/T | C/C | C/C | T/T |
| 619 | C56 | T/T | C/C | T/T | C/C | A/A | T/T | C/C | C/C | T/T | C/C | C/C | T/T |
| 620 | C57 | T/T | C/C | T/T |  | A/A |  |  | C/C | T/T | C/C |  |  |
| 621 | C58 | T/T | C/C | T/T | C/C | A/A | T/T | C/C | C/C | T/T | C/C |  | T/T |
| 622 | C59 | T/T | C/C | T/T | C/C | A/A | T/T | C/C | C/C | T/T | C/C | C/C | T/T |
| 623 | C60 |  |  |  | C/C | A/A | T/T | C/C | C/C | T/T | C/C | C/C | T/T |
| 624 | C61 |  | C/C | T/T | C/C | A/A | T/T | C/C | T/C | T/T | C/C | C/C | T/T |
| 625 | C62 | T/T | C/C | T/T | C/C | A/A |  |  |  | T/T | C/C | C/C | T/T |
| 626 | ArinaLrFor |  | C/C | T/T |  | A/A | T/T | C/C | C/C | T/T | C/C | C/C |  |
| 627 | CDC_Landmark | C/C | C/C | C/C | A/A |  |  |  |  |  |  |  |  |
| 628 | CDC_Stanley | T/T |  | T/T | C/C |  | T/T | C/C |  |  |  | C/C |  |
| 629 | Cadenza | T/T | A/A | T/T | C/C | A/A | T/T | C/C | C/C | T/T | C/C |  |  |
| 630 | Claire | T/T | C/C | T/T | C/C | A/A | T/T | C/C |  | T/T | C/C |  |  |
| 631 | Jagger | T/T | A/A | T/T | C/C | A/A | T/T | C/C | C/C | T/T | C/C | C/C | T/T |
| 632 | Julius | T/T | A/A | T/T |  | A/A | T/T | C/C | C/C | T/T | C/C |  |  |
| 633 | Lancer | T/T | C/C | T/T |  | A/A | T/T | C/C |  | T/T | C/C |  |  |
| 634 | Mace | T/T | C/C | T/T |  | A/A | T/T | C/C | C/C | T/T | C/C | C/C | T/T |
| 635 | Norin61 | T/T | C/C | T/T | C/C | A/A | T/T | C/C | T/T |  |  | C/C |  |
| 636 | Paragon | T/T | A/A | T/T | C/C | A/A | T/T | C/C | C/C | T/T | C/C |  |  |
| 637 | Robigus | T/T | C/C | T/T | C/C | A/A |  |  | C/C | T/T | C/C |  |  |
| 638 | SY_Mattis | T/T | C/C | T/T | C/C | A/A | T/T | C/C |  |  |  | C/C | T/T |
| 639 | Spelt |  | C/C |  |  |  |  |  |  |  |  |  |  |
| 640 | Weebil | T/T | C/C | T/T | C/C | A/A | T/T | C/C | C/C | T/T | C/C | C/C | T/T |
| 641 | Z8425B |  |  |  |  |  |  |  | C/C |  |  |  |  |
| 642 | PH46 | T/T | C/C | T/T | C/C |  | T/T | C/C | T/T | T/T | C/C | C/C | T/T |
| 643 | PH49 | T/T | C/C |  | C/C |  | T/T | C/C | C/C |  |  |  |  |
| 644 | PH51 | T/T | C/C | T/T | C/C | A/A | T/T | C/C |  | T/T | C/C | C/C | T/T |
| 645 | PH52 | T/T | C/C | T/T |  | A/A |  | C/C | C/C | T/T | C/C | C/C | T/T |
| 646 | PH56 |  |  | T/T |  | A/A | T/T | C/C | C/C | T/T | C/C | C/C |  |
| 647 | PH68 | T/T | C/C | T/T | C/C | A/A | T/T | C/C | T/T | T/T | C/C | C/C | T/T |
| 648 | PH148 | T/T | C/C | T/T | C/C | A/A | T/T | C/C | T/T | T/T | C/C | C/C | T/T |
| 649 | PH95 | T/T | C/C | T/T | C/C | A/A | T/T | C/C | C/C | T/T | C/C | C/C | T/T |
| 650 | PH105 | T/T | C/C | T/T | C/C |  | T/T | C/C | C/C | T/T | C/C |  |  |
| 651 | PH108 | T/T | C/C | T/T | C/C | A/A | T/T | C/C | C/C | T/T | C/C | C/C | T/T |
| 652 | PH135 | T/T | C/C | T/T | C/C |  |  |  | C/C |  |  | C/C | T/T |
| 653 | PH151 | T/T | A/A | T/T | C/C | A/A | T/T | C/C | C/C | T/T | C/C | C/C | T/T |
| 654 | PH153 |  | C/C | T/T | C/C | A/A | T/T | C/C | C/C | T/T | C/C | C/C | T/T |
| 655 | PH126 | T/T | C/C | T/T | C/C | A/A | T/T | C/C | C/C |  | C/C | C/C | T/T |
| 656 | PH132 | T/T | C/C | T/T | C/C | A/A | T/T | C/C | C/C | T/T | C/C | C/C | T/T |
| 657 | PH133 | T/T | C/C | T/T | C/C | A/A | T/T | C/C |  | T/T | C/C | C/C | T/T |
| 658 | PH09 | T/T | C/C | T/T | C/C | A/A | T/T | C/C | C/C | T/T |  | C/C | T/T |
| 659 | XM01 | T/T | C/C | T/T | C/C | A/A | T/T | C/C | C/C | T/T | C/C | C/C | T/T |
| 660 | PH10 | T/T | C/C | T/T | C/C | A/A | T/T | C/C | C/C | T/T | C/C | C/C | T/T |
| 661 | NZ05 | T/T | C/C | T/T | C/C | A/A | T/T | C/C | C/C | T/T | C/C | C/C | T/T |
| 662 | YM05 |  | C/C | T/T | C/C | A/A | T/T | C/C | C/C | T/T | C/C | C/C | T/T |
| 663 | YM02 | T/T | C/C | T/T | C/C | A/A |  |  | C/C | T/T | C/C | C/C | T/T |
| 664 | YM07 | T/T | C/C | T/T | C/C | A/A | T/T | C/C | C/C | T/T | C/C | C/C | T/T |
| 665 | YM03 | T/T | C/C | T/T | C/C | A/A | T/T | C/C | C/C | T/T | C/C | C/C | T/T |
| 666 | NZ10 |  | C/C | T/T | C/C | A/A | T/T | C/C | C/C |  |  |  | T/T |
| 667 | S141 | T/T | C/C |  | C/C | A/A | T/T | C/C | C/C | T/T | C/C | C/C | T/T |
| 668 | AS360 | T/T | C/C |  | C/C | A/A | T/T | C/C | C/C |  |  |  | T/T |
| 669 | AS362 | T/T | C/C |  |  | A/A | T/T | C/C | C/C | T/T | C/C | C/C | T/T |
| 670 | 18C429 | T/T | C/C | T/T | C/C |  |  |  |  | T/T | C/C | C/C | T/T |
| 671 | 18C430 | T/T | C/C | T/T | C/C | A/A |  |  |  | T/T | C/C | C/C | T/T |
| 672 | 18C448 | T/T | C/C | T/T | C/C | A/A | T/T | C/C |  |  |  | C/C | T/T |
| 673 | 18C450 | T/T | C/C |  | C/C | A/A | T/T | C/C | C/C | T/T | C/C |  |  |
| 674 | 3987 | T/T | C/C | T/T | C/C | A/A | T/T | C/C | C/C |  |  | C/C |  |
| 675 | 3989 | T/T | C/C | T/T | C/C | A/A | T/T | C/C | C/C | T/T | C/C | C/C | T/T |
| 676 | 3970 | T/T | C/C | T/T | C/C | A/A | T/T | C/C | C/C | T/T | C/C | C/C | T/T |
| 677 | AS356 | T/T | C/C | T/T |  | A/A | T/T | C/C | C/C |  | C/C | C/C | T/T |
| 678 | XD2 |  |  |  |  |  |  |  | C/C |  |  |  |  |
| 679 | XLM2 |  |  |  |  |  |  |  | C/C |  |  |  |  |
| 680 | XS2 |  |  |  |  |  |  |  | C/C |  |  |  |  |
| 681 | XZ892 |  |  |  |  |  |  |  | C/C |  |  |  |  |

| **Table S6.** Association analysis of TKW, KL, KW and KT between two haplotypes of *TaUBC25* gene. | | | | |
| --- | --- | --- | --- | --- |
| **Years and locations** | **Kernel traits** | **Haplotypes** | **Mean±SE** | ***P* value** |
| **2002LY** | TKW | *TaUBC25-HapⅠ* | 34.51±5.76 | 1.29E-07^**^ |
|  |  | *TaUBC25-HapⅡ* | 42.39±6.75 |  |
|  | KL | *TaUBC25-HapⅠ* | 0.61±0.06 | 1.53E-06^**^ |
|  |  | *TaUBC25-HapⅡ* | 0.67±0.06 |  |
|  | KW | *TaUBC25-HapⅠ* | 0.3±0.03 | 3.54E-07^**^ |
|  |  | *TaUBC25-HapⅡ* | 0.33±0.03 |  |
|  | KT | *TaUBC25-HapⅠ* | 0.27±0.03 | 2.44E-06^**^ |
|  |  | *TaUBC25-HapⅡ* | 0.29±0.02 |  |
| **2005LY** | TKW | *TaUBC25-HapⅠ* | 30.62±5.37 | 1.11E-11^**^ |
|  |  | *TaUBC25-HapⅡ* | 39.8±6.28 |  |
|  | KL | *TaUBC25-HapⅠ* | 0.62±0.05 | 4.03E-09^**^ |
|  |  | *TaUBC25-HapⅡ* | 0.68±0.05 |  |
|  | KW | *TaUBC25-HapⅠ* | 0.3±0.02 | 7.75E-11^**^ |
|  |  | *TaUBC25-HapⅡ* | 0.32±0.02 |  |
|  | KT | *TaUBC25-HapⅠ* | 0.27±0.02 | 5.53E-07^**^ |
|  |  | *TaUBC25-HapⅡ* | 0.29±0.02 |  |
| **2010SY** | TKW | *TaUBC25-HapⅠ* | 32.59±5.56 | 2.39E-08^**^ |
|  |  | *TaUBC25-HapⅡ* | 40.06±6.92 |  |
|  | KL | *TaUBC25-HapⅠ* | 0.65±0.05 | 4.31E-07^**^ |
|  |  | *TaUBC25-HapⅡ* | 0.7±0.05 |  |
|  | KW | *TaUBC25-HapⅠ* | 0.3±0.02 | 5.68E-09^**^ |
|  |  | *TaUBC25-HapⅡ* | 0.32±0.02 |  |
|  | KT | *TaUBC25-HapⅠ* | 0.29±0.03 | 0.0375^*^ |
|  |  | *TaUBC25-HapⅡ* | 0.3±0.02 |  |

**Note:** TKW, thousand kernel weight. KL, kernel length. KW, kernel width. KT, kernel thickness.

2002 LY, 2002 Luoyang. 2005 LY, 2005 Luoyang. 2010 SY, Shunyi.

| **Table S7.** Wheat diversity panel information and distribution of *TaUBC25* alleles among genotypes. | | | | | | |
| --- | --- | --- | --- | --- | --- | --- |
| **Number** | **Resequencing**  **cultivar ID** | **Accession** | **Nation** | **Origin** | **Allele** | **Source** |
| 1 | MP021 | YanDai1817 | China | Beijing | HapⅠ-T | WheatUnion |
| 2 | KFEH | Fengkang2 | China | Beijing | HapⅡ-C | WheatUnion |
| 3 | MP118 | LunXuan987 | China | Beijing | HapⅡ-C | WheatUnion |
| 4 | MP138 | KeChun14 | China | Beijing | HapⅡ-C | WheatUnion |
| 5 | S67 | YC_NongDa3338 | China | Beijing | HapⅡ-C | WheatUnion |
| 6 | S140 | YC_NongDa5181 | China | Beijing | HapⅡ-C | WheatUnion |
| 7 | S212 | YCL_Lovrin10 | China | Beijing | HapⅡ-C | WheatUnion |
| 8 | S225 | YCL_BeiJing8 | China | Beijing | HapⅡ-C | WheatUnion |
| 9 | S271 | YC_LX987 | China | Beijing | HapⅡ-C | WheatUnion |
| 10 | S273 | YC_Jing411 | China | Beijing | HapⅡ-C | WheatUnion |
| 11 | S274 | YC_NongDa3097 | China | Beijing | HapⅡ-C | WheatUnion |
| 12 | S6554 | YC_JingDong6 | China | Beijing | HapⅡ-C | WheatUnion |
| 13 | PH132 | Nongda179 | China | Beijing | HapⅡ-C | WheatUnion |
| 14 | YM02 | Nongda981 | China | Beijing | HapⅡ-C | WheatUnion |
| 15 | YM07 | Nongda3753 | China | Beijing | HapⅡ-C | WheatUnion |
| 16 | YM03 | Nongda3097 | China | Beijing | HapⅡ-C | WheatUnion |
| 17 | S101 | DF_GSHongQiMai | China | Gansu | HapⅠ-T | WheatUnion |
| 18 | S102 | DF_GSDuanYaoMai | China | Gansu | HapⅠ-T | WheatUnion |
| 19 | S105 | DF_GSHongHuoSui | China | Gansu | HapⅠ-T | WheatUnion |
| 20 | S240 | DF_GSLanHuaMai | China | Gansu | HapⅠ-T | WheatUnion |
| 21 | S250 | DF_GSYiZhiMai | China | Gansu | HapⅠ-T | WheatUnion |
| 22 | S251 | DF_GSHuoLiYan | China | Gansu | HapⅠ-T | WheatUnion |
| 23 | HMM | Hongmangmai | China | Gansu | HapⅡ-C | WheatUnion |
| 24 | HNSH | Huining10 | China | Gansu | HapⅡ-C | WheatUnion |
| 25 | MP004 | CI 12203 | China | Gansu | HapⅡ-C | WheatUnion |
| 26 | MP033 | HeZhouDaiMai | China | Gansu | HapⅡ-C | WheatUnion |
| 27 | MP056 | GanMai8Hao | China | Gansu | HapⅡ-C | WheatUnion |
| 28 | S103 | DF_GSDaBaiMai | China | Gansu | HapⅡ-C | WheatUnion |
| 29 | S104 | DF_GSBaiMuXianBan | China | Gansu | HapⅡ-C | WheatUnion |
| 30 | S106 | DF_GSBaiDaTou | China | Gansu | HapⅡ-C | WheatUnion |
| 31 | C36 | LR_China1 | China | Hebei | HapⅠ-T | WheatUnion |
| 32 | MP081 | JiMai26 | China | Hebei | HapⅡ-C | WheatUnion |
| 33 | MP098 | JiMai38 | China | Hebei | HapⅡ-C | WheatUnion |
| 34 | MP099 | GaoYou503 | China | Hebei | HapⅡ-C | WheatUnion |
| 35 | MP104 | GaoCheng8901 | China | Hebei | HapⅡ-C | WheatUnion |
| 36 | MP109 | HanDan6172 | China | Hebei | HapⅡ-C | WheatUnion |
| 37 | MP111 | DanJiaZhuang8Hao | China | Hebei | HapⅡ-C | WheatUnion |
| 38 | MP120 | HengGuan35 | China | Hebei | HapⅡ-C | WheatUnion |
| 39 | MP141 | JiMai30 | China | Hebei | HapⅡ-C | WheatUnion |
| 40 | MP145 | ZhongYou9507 | China | Hebei | HapⅡ-C | WheatUnion |
| 41 | S64 | YC_Shi4185 | China | Hebei | HapⅡ-C | WheatUnion |
| 42 | S142 | YC_ShiLuan02d1 | China | Hebei | HapⅡ-C | WheatUnion |
| 43 | S227 | YCL_ShiJiaZhuang54 | China | Hebei | HapⅡ-C | WheatUnion |
| 44 | MP027 | FuMai | China | Henan | HapⅠ-T | WheatUnion |
| 45 | MP051 | NeiXiang5Hao | China | Henan | HapⅠ-T | WheatUnion |
| 46 | S61 | BS_HNPuYang23 | China | Henan | HapⅠ-T | WheatUnion |
| 47 | CSB | Chushanbao | China | Henan | HapⅡ-C | WheatUnion |
| 48 | PYEQ | Pingyang27 | China | Henan | HapⅡ-C | WheatUnion |
| 49 | MP009 | St 1472/506 (ZhengYin1Hao) | China | Henan | HapⅡ-C | WheatUnion |
| 50 | MP023 | YouZiMai | China | Henan | HapⅡ-C | WheatUnion |
| 51 | MP034 | GuangTouBaiSanMai | China | Henan | HapⅡ-C | WheatUnion |
| 52 | MP060 | BoAi7023 | China | Henan | HapⅡ-C | WheatUnion |
| 53 | MP064 | YanShi4Hao | China | Henan | HapⅡ-C | WheatUnion |
| 54 | MP065 | BoNong3217 | China | Henan | HapⅡ-C | WheatUnion |
| 55 | MP071 | YuMai2Hao | China | Henan | HapⅡ-C | WheatUnion |
| 56 | MP075 | YuMai7Hao | China | Henan | HapⅡ-C | WheatUnion |
| 57 | MP082 | Zhou8425B | China | Henan | HapⅡ-C | WheatUnion |
| 58 | MP083 | YuMai13 | China | Henan | HapⅡ-C | WheatUnion |
| 59 | MP087 | YuMai17 | China | Henan | HapⅡ-C | WheatUnion |
| 60 | MP091 | YuMai21Hao (ZhouMai9Hao) | China | Henan | HapⅡ-C | WheatUnion |
| 61 | MP097 | YuMai41 | China | Henan | HapⅡ-C | WheatUnion |
| 62 | MP103 | WenMai6Hao (YuMai49) | China | Henan | HapⅡ-C | WheatUnion |
| 63 | MP110 | ZhengMai9023 | China | Henan | HapⅡ-C | WheatUnion |
| 64 | MP113 | ZhouMai16 | China | Henan | HapⅡ-C | WheatUnion |
| 65 | MP117 | YanZhan4110 | China | Henan | HapⅡ-C | WheatUnion |
| 66 | MP124 | ZhengMai366 | China | Henan | HapⅡ-C | WheatUnion |
| 67 | MP125 | BoNongAK58 | China | Henan | HapⅡ-C | WheatUnion |
| 68 | MP128 | ZhouMai22Hao | China | Henan | HapⅡ-C | WheatUnion |
| 69 | MP131 | XinMai26 | China | Henan | HapⅡ-C | WheatUnion |
| 70 | MP132 | FengDeCunMai5Hao | China | Henan | HapⅡ-C | WheatUnion |
| 71 | MP134 | ZhongMai66 | China | Henan | HapⅡ-C | WheatUnion |
| 72 | MP135 | BoNong201 | China | Henan | HapⅡ-C | WheatUnion |
| 73 | MP136 | BoNong4199 | China | Henan | HapⅡ-C | WheatUnion |
| 74 | S10 | DF_HNPingYuan50 | China | Henan | HapⅡ-C | WheatUnion |
| 75 | S60 | BS_HNPuYang21 | China | Henan | HapⅡ-C | WheatUnion |
| 76 | S137 | YC_LuMai21 | China | Henan | HapⅡ-C | WheatUnion |
| 77 | S139 | YC_YuMai18 | China | Henan | HapⅡ-C | WheatUnion |
| 78 | S217 | YCL_ZhengMai9023 | China | Henan | HapⅡ-C | WheatUnion |
| 79 | S218 | YCL_YanZhan1 | China | Henan | HapⅡ-C | WheatUnion |
| 80 | S221 | YCL_YuMai21 | China | Henan | HapⅡ-C | WheatUnion |
| 81 | S223 | YCL_ZhengYin4 | China | Henan | HapⅡ-C | WheatUnion |
| 82 | S224 | YCL_BaiNong3217 | China | Henan | HapⅡ-C | WheatUnion |
| 83 | C42 | VA_China2 | China | Henan | HapⅡ-C | WheatUnion |
| 84 | C46 | VA_China6 | China | Henan | HapⅡ-C | WheatUnion |
| 85 | PH135 | Zhongmai875 | China | Henan | HapⅡ-C | WheatUnion |
| 86 | SRM4H | Songruimai4 | China | Jiangsu | HapⅠ-T | WheatUnion |
| 87 | MP022 | JiangDongMen | China | Jiangsu | HapⅠ-T | WheatUnion |
| 88 | MP045 | WangShuiBai | China | Jiangsu | HapⅠ-T | WheatUnion |
| 89 | MP054 | XuZhou14 | China | Jiangsu | HapⅡ-C | WheatUnion |
| 90 | MP069 | ShanNong7859 | China | Jiangsu | HapⅡ-C | WheatUnion |
| 91 | MP077 | YangMai5Hao | China | Jiangsu | HapⅡ-C | WheatUnion |
| 92 | MP092 | YangMai158 | China | Jiangsu | HapⅡ-C | WheatUnion |
| 93 | MP112 | HuaiMai20 | China | Jiangsu | HapⅡ-C | WheatUnion |
| 94 | MP133 | XuMai35 | China | Jiangsu | HapⅡ-C | WheatUnion |
| 95 | S210 | YCL_SuiMai3 | China | Jiangsu | HapⅡ-C | WheatUnion |
| 96 | C41 | VA_China1 | China | Jiangsu | HapⅡ-C | WheatUnion |
| 97 | LTT | Laotutou | China | Ningxia | HapⅠ-T | WheatUnion |
| 98 | S108 | DF_NXShanMai | China | Ningxia | HapⅠ-T | WheatUnion |
| 99 | HTZ | Hongtuzi | China | Ningxia | HapⅡ-C | WheatUnion |
| 100 | MP042 | BaiMaZha | China | Ningxia | HapⅡ-C | WheatUnion |
| 101 | MP066 | NingChun4Hao (YongLiang4Hao) | China | Ningxia | HapⅡ-C | WheatUnion |
| 102 | S186 | DF_SDBaiBianSui | China | Shandong | HapⅠ-T | WheatUnion |
| 103 | PH68 | Yuejin5 | China | Shandong | HapⅠ-T | WheatUnion |
| 104 | AMN | Aimengniu | China | Shandong | HapⅡ-C | WheatUnion |
| 105 | XYLH | Xiaoyan6 | China | Shandong | HapⅡ-C | WheatUnion |
| 106 | MP039 | ZiJieHong | China | Shandong | HapⅡ-C | WheatUnion |
| 107 | MP050 | YueJin5Hao | China | Shandong | HapⅡ-C | WheatUnion |
| 108 | MP057 | JiNan9Hao | China | Shandong | HapⅡ-C | WheatUnion |
| 109 | MP059 | JiNing3Hao | China | Shandong | HapⅡ-C | WheatUnion |
| 110 | MP062 | TaiShan1Hao | China | Shandong | HapⅡ-C | WheatUnion |
| 111 | MP068 | ShanNongFu63 | China | Shandong | HapⅡ-C | WheatUnion |
| 112 | MP072 | LuMai1Hao (AiMengNiu) | China | Shandong | HapⅡ-C | WheatUnion |
| 113 | MP074 | JiNan13 | China | Shandong | HapⅡ-C | WheatUnion |
| 114 | MP086 | LuMai14 | China | Shandong | HapⅡ-C | WheatUnion |
| 115 | MP089 | LuMai15 | China | Shandong | HapⅡ-C | WheatUnion |
| 116 | MP090 | PH82-2-2 | China | Shandong | HapⅡ-C | WheatUnion |
| 117 | MP096 | LuMai21 | China | Shandong | HapⅡ-C | WheatUnion |
| 118 | MP102 | JiNan16 | China | Shandong | HapⅡ-C | WheatUnion |
| 119 | MP106 | JiNan17 | China | Shandong | HapⅡ-C | WheatUnion |
| 120 | MP108 | JiMai19 | China | Shandong | HapⅡ-C | WheatUnion |
| 121 | MP114 | YanNong21Hao | China | Shandong | HapⅡ-C | WheatUnion |
| 122 | MP115 | JiMai20 | China | Shandong | HapⅡ-C | WheatUnion |
| 123 | MP127 | JiMai22 | China | Shandong | HapⅡ-C | WheatUnion |
| 124 | MP130 | LiangXing66 | China | Shandong | HapⅡ-C | WheatUnion |
| 125 | S15 | BS_SDNingYang5 | China | Shandong | HapⅡ-C | WheatUnion |
| 126 | S62 | BS_SDChangQing49 | China | Shandong | HapⅡ-C | WheatUnion |
| 127 | S136 | YC_YanNong15 | China | Shandong | HapⅡ-C | WheatUnion |
| 128 | S187 | DF_SDDaLiBanMang | China | Shandong | HapⅡ-C | WheatUnion |
| 129 | S188 | DF_SDLaoLaiXia | China | Shandong | HapⅡ-C | WheatUnion |
| 130 | S215 | YCL_JiNan17 | China | Shandong | HapⅡ-C | WheatUnion |
| 131 | S220 | YCL_TaiShan1 | China | Shandong | HapⅡ-C | WheatUnion |
| 132 | S272 | YC_JiMai22 | China | Shandong | HapⅡ-C | WheatUnion |
| 133 | C44 | VA_China4 | China | Shandong | HapⅡ-C | WheatUnion |
| 134 | YM05 | Liangxing99 | China | Shandong | HapⅡ-C | WheatUnion |
| 135 | JiaHM | Jiahongmai | China | Shanxi | HapⅠ-T | WheatUnion |
| 136 | S181 | DF_SXBaiHuoMai | China | Shanxi | HapⅠ-T | WheatUnion |
| 137 | DHM | Dahongmai | China | Shanxi | HapⅡ-C | WheatUnion |
| 138 | MP061 | JinMai10Hao (WeiDong8Hao) | China | Shanxi | HapⅡ-C | WheatUnion |
| 139 | MP094 | JinMai47 | China | Shanxi | HapⅡ-C | WheatUnion |
| 140 | MP129 | ZhongMai175 | China | Shanxi | HapⅡ-C | WheatUnion |
| 141 | S209 | YCL_ChangZhi6406 | China | Shanxi | HapⅡ-C | WheatUnion |
| 142 | HM | Huomai | China | Shaanxi | HapⅠ-T | WheatUnion |
| 143 | HZB | Hanzhongbai | China | Shaanxi | HapⅠ-T | WheatUnion |
| 144 | TMM | Tumangmai | China | Shaanxi | HapⅠ-T | WheatUnion |
| 145 | MP046 | BiMa1Hao | China | Shaanxi | HapⅠ-T | WheatUnion |
| 146 | MP049 | XiNong6028 | China | Shaanxi | HapⅠ-T | WheatUnion |
| 147 | S182 | DF_SHXChunXiaoMai | China | Shaanxi | HapⅠ-T | WheatUnion |
| 148 | S183 | DF_SHXMaZhaMai | China | Shaanxi | HapⅠ-T | WheatUnion |
| 149 | S213 | YCL_BiMa4 | China | Shaanxi | HapⅠ-T | WheatUnion |
| 150 | S214 | YCL_FengChan3 | China | Shaanxi | HapⅠ-T | WheatUnion |
| 151 | LM | Laomai | China | Shaanxi | HapⅡ-C | WheatUnion |
| 152 | MP063 | AiFeng3Hao | China | Shaanxi | HapⅡ-C | WheatUnion |
| 153 | MP067 | XiaoYan6Hao | China | Shaanxi | HapⅡ-C | WheatUnion |
| 154 | MP095 | XiNong881 | China | Shaanxi | HapⅡ-C | WheatUnion |
| 155 | MP139 | XiaoYan96 | China | Shaanxi | HapⅡ-C | WheatUnion |
| 156 | MP142 | XiNong65 | China | Shaanxi | HapⅡ-C | WheatUnion |
| 157 | MP143 | Shan213 | China | Shaanxi | HapⅡ-C | WheatUnion |
| 158 | C43 | VA_China3 | China | Shaanxi | HapⅡ-C | WheatUnion |
| 159 | C47 | VA_China7 | China | Shaanxi | HapⅡ-C | WheatUnion |
| 160 | TJBXM | Tongjiabaxiaomai | China | Sichuan | HapⅠ-T | WheatUnion |
| 161 | ZP | Zipi | China | Sichuan | HapⅠ-T | WheatUnion |
| 162 | MP025 | ChengDouGuangTou | China | Sichuan | HapⅠ-T | WheatUnion |
| 163 | MP026 | ZhongGuoChun | China | Sichuan | HapⅠ-T | WheatUnion |
| 164 | S94 | DF_SC1666 | China | Sichuan | HapⅠ-T | WheatUnion |
| 165 | S95 | DF_SC1587 | China | Sichuan | HapⅠ-T | WheatUnion |
| 166 | S96 | DF_SC1635 | China | Sichuan | HapⅠ-T | WheatUnion |
| 167 | S97 | DF_SC1588 | China | Sichuan | HapⅠ-T | WheatUnion |
| 168 | S98 | DF_SC1668 | China | Sichuan | HapⅠ-T | WheatUnion |
| 169 | S99 | DF_SC1661 | China | Sichuan | HapⅠ-T | WheatUnion |
| 170 | S100 | DF_SC1670 | China | Sichuan | HapⅠ-T | WheatUnion |
| 171 | S249 | DF_SCKangDing1 | China | Sichuan | HapⅠ-T | WheatUnion |
| 172 | MP048 | WuYiMai | China | Sichuan | HapⅡ-C | WheatUnion |
| 173 | MP070 | MianYang11 | China | Sichuan | HapⅡ-C | WheatUnion |
| 174 | MP073 | MianYang15 | China | Sichuan | HapⅡ-C | WheatUnion |
| 175 | MP085 | ChuanMai22 | China | Sichuan | HapⅡ-C | WheatUnion |
| 176 | MP093 | MianYang26Hao | China | Sichuan | HapⅡ-C | WheatUnion |
| 177 | S216 | YCL_Fan6 | China | Sichuan | HapⅡ-C | WheatUnion |
| 178 | C45 | VA_China5 | China | Sichuan | HapⅡ-C | WheatUnion |
| 179 | TW033 | XM0909 | China | Tibet | HapⅠ-T | WheatUnion |
| 180 | S3 | XZbys_JiTang1387 | China | Tibet | HapⅠ-T | WheatUnion |
| 181 | S16 | XZbys_ZuoGongSuiSui1 | China | Tibet | HapⅠ-T | WheatUnion |
| 182 | S23 | XZbys_LangXianSuiSui15 | China | Tibet | HapⅠ-T | WheatUnion |
| 183 | S24 | XZbys_LangXianSuiSui17 | China | Tibet | HapⅠ-T | WheatUnion |
| 184 | S40 | XZbys_LangXianZheDa35 | China | Tibet | HapⅠ-T | WheatUnion |
| 185 | S52 | XZD_GongBuJiangDa1523 | China | Tibet | HapⅠ-T | WheatUnion |
| 186 | S81 | XZD_NaiDong1500 | China | Tibet | HapⅠ-T | WheatUnion |
| 187 | S89 | XZD_GongBuJiangDa1526 | China | Tibet | HapⅠ-T | WheatUnion |
| 188 | S145 | XZbys_ChaYaZheDa49 | China | Tibet | HapⅠ-T | WheatUnion |
| 189 | S162 | XZbys_LongZiZheDa34 | China | Tibet | HapⅠ-T | WheatUnion |
| 190 | S244 | XZD_MoTuo | China | Tibet | HapⅠ-T | WheatUnion |
| 191 | S248 | XZD_MoZongZhuoGa | China | Tibet | HapⅠ-T | WheatUnion |
| 192 | S255 | XZbys_ChaYuSuiSui2 | China | Tibet | HapⅠ-T | WheatUnion |
| 193 | S257 | XZbys_ChaYuSuiSui5 | China | Tibet | HapⅠ-T | WheatUnion |
| 194 | S259 | XZbys_ChaYuZheDa5 | China | Tibet | HapⅠ-T | WheatUnion |
| 195 | S262 | XZbys_JiaChaZheDa61 | China | Tibet | HapⅠ-T | WheatUnion |
| 196 | Zang1817 | XZbys_Zang1817 (Zang1817) | China | Tibet | HapⅡ-C | WheatUnion |
| 197 | GYWLL | Gaoyuan506 | China | Tibet | HapⅡ-C | WheatUnion |
| 198 | ZDSH | Zangdong4 | China | Tibet | HapⅡ-C | WheatUnion |
| 199 | S1 | XZbys_ZuoGong1203 | China | Tibet | HapⅡ-C | WheatUnion |
| 200 | S2 | XZbys_LangXian1274 | China | Tibet | HapⅡ-C | WheatUnion |
| 201 | S4 | XZD_DuiLongDQ1289 | China | Tibet | HapⅡ-C | WheatUnion |
| 202 | S5 | XZD_MoZhuGK1772 | China | Tibet | HapⅡ-C | WheatUnion |
| 203 | S13 | XZMiSui | China | Tibet | HapⅡ-C | WheatUnion |
| 204 | S14 | XZD_ZXM1341 | China | Tibet | HapⅡ-C | WheatUnion |
| 205 | S17 | XZbys_JiaChaSuiSui8 | China | Tibet | HapⅡ-C | WheatUnion |
| 206 | S18 | XZbys_JiaChaSuiSui27 | China | Tibet | HapⅡ-C | WheatUnion |
| 207 | S19 | XZbys_JiaChaSuiSui32 | China | Tibet | HapⅡ-C | WheatUnion |
| 208 | S20 | XZbys_JiaChaSuiSui33 | China | Tibet | HapⅡ-C | WheatUnion |
| 209 | S21 | XZbys_JiaChaSuiSui34 | China | Tibet | HapⅡ-C | WheatUnion |
| 210 | S22 | XZbys_LangXianSuiSui9 | China | Tibet | HapⅡ-C | WheatUnion |
| 211 | S25 | XZbys_LongZiSuiSui10 | China | Tibet | HapⅡ-C | WheatUnion |
| 212 | S26 | XZbys_LongZiSuiSui11 | China | Tibet | HapⅡ-C | WheatUnion |
| 213 | S27 | XZbys_LongZiSuiSui17 | China | Tibet | HapⅡ-C | WheatUnion |
| 214 | S28 | XZbys_LongZiSuiSui23 | China | Tibet | HapⅡ-C | WheatUnion |
| 215 | S29 | XZbys_LongZiSuiSui24 | China | Tibet | HapⅡ-C | WheatUnion |
| 216 | S30 | XZbys_ChaYaZheDa14 | China | Tibet | HapⅡ-C | WheatUnion |
| 217 | S31 | XZbys_ChaYaZheDa18 | China | Tibet | HapⅡ-C | WheatUnion |
| 218 | S32 | XZbys_ChaYaZheDa19 | China | Tibet | HapⅡ-C | WheatUnion |
| 219 | S33 | XZbys_ChaYaZheDa32 | China | Tibet | HapⅡ-C | WheatUnion |
| 220 | S34 | XZbys_JiaChaZheDa9 | China | Tibet | HapⅡ-C | WheatUnion |
| 221 | S35 | XZbys_JiaChaZheDa55 | China | Tibet | HapⅡ-C | WheatUnion |
| 222 | S36 | XZbys_JiaChaZheDa63 | China | Tibet | HapⅡ-C | WheatUnion |
| 223 | S37 | XZbys_LangXianZheDa3 | China | Tibet | HapⅡ-C | WheatUnion |
| 224 | S38 | XZbys_LangXianZheDa4 | China | Tibet | HapⅡ-C | WheatUnion |
| 225 | S39 | XZbys_LangXianZheDa20 | China | Tibet | HapⅡ-C | WheatUnion |
| 226 | S41 | XZbys_LongZiZheDa4 | China | Tibet | HapⅡ-C | WheatUnion |
| 227 | S42 | XZbys_LongZiZheDa9 | China | Tibet | HapⅡ-C | WheatUnion |
| 228 | S43 | XZbys_LongZiZheDa19 | China | Tibet | HapⅡ-C | WheatUnion |
| 229 | S44 | XZbys_LongZiZheDa27 | China | Tibet | HapⅡ-C | WheatUnion |
| 230 | S45 | XZbys_SangRiZheDa21 | China | Tibet | HapⅡ-C | WheatUnion |
| 231 | S46 | XZD_RiKaZeLaSa1301 | China | Tibet | HapⅡ-C | WheatUnion |
| 232 | S47 | XZD_QuShui1316 | China | Tibet | HapⅡ-C | WheatUnion |
| 233 | S48 | XZD_GongGa1348 | China | Tibet | HapⅡ-C | WheatUnion |
| 234 | S49 | XZD_JiangZi1405 | China | Tibet | HapⅡ-C | WheatUnion |
| 235 | S50 | XZD_NaiDongQJ1494 | China | Tibet | HapⅡ-C | WheatUnion |
| 236 | S51 | XZD_ZeDang1511 | China | Tibet | HapⅡ-C | WheatUnion |
| 237 | S72 | XZD_ZeDang1516 | China | Tibet | HapⅡ-C | WheatUnion |
| 238 | S73 | XZD_ZeDang1513 | China | Tibet | HapⅡ-C | WheatUnion |
| 239 | S74 | XZD_RiKaZeLaSa1373 | China | Tibet | HapⅡ-C | WheatUnion |
| 240 | S75 | XZD_RiKaZeLaSa1367 | China | Tibet | HapⅡ-C | WheatUnion |
| 241 | S77 | XZD_QuShui1409 | China | Tibet | HapⅡ-C | WheatUnion |
| 242 | S78 | XZD_QuShui1340 | China | Tibet | HapⅡ-C | WheatUnion |
| 243 | S79 | XZD_QiongJie1483 | China | Tibet | HapⅡ-C | WheatUnion |
| 244 | S82 | XZD_MoZhuGK2065 | China | Tibet | HapⅡ-C | WheatUnion |
| 245 | S83 | XZD_MoZhuGK2064 | China | Tibet | HapⅡ-C | WheatUnion |
| 246 | S84 | XZD_MoZhuGK2050 | China | Tibet | HapⅡ-C | WheatUnion |
| 247 | S86 | XZD_LaSa1342 | China | Tibet | HapⅡ-C | WheatUnion |
| 248 | S87 | XZD_JiangZi1407 | China | Tibet | HapⅡ-C | WheatUnion |
| 249 | S90 | XZD_GongBuJiangDa1525 | China | Tibet | HapⅡ-C | WheatUnion |
| 250 | S91 | XZD_DuiLongDeQing1521 | China | Tibet | HapⅡ-C | WheatUnion |
| 251 | S92 | XZD_DuiLongDeQing1284 | China | Tibet | HapⅡ-C | WheatUnion |
| 252 | S143 | XZbys_LangXianSuiSui6 | China | Tibet | HapⅡ-C | WheatUnion |
| 253 | S144 | XZbys_LangXianSuiSui18 | China | Tibet | HapⅡ-C | WheatUnion |
| 254 | S146 | XZbys_JiaChaZheDa28 | China | Tibet | HapⅡ-C | WheatUnion |
| 255 | S147 | XZbys_JiaChaZheDa37 | China | Tibet | HapⅡ-C | WheatUnion |
| 256 | S148 | XZbys_JiaChaZheDa39 | China | Tibet | HapⅡ-C | WheatUnion |
| 257 | S149 | XZbys_LangXianZheDa5 | China | Tibet | HapⅡ-C | WheatUnion |
| 258 | S150 | XZbys_LangXianZheDa6 | China | Tibet | HapⅡ-C | WheatUnion |
| 259 | S151 | XZbys_LangXianZheDa7 | China | Tibet | HapⅡ-C | WheatUnion |
| 260 | S152 | XZbys_LangXianZheDa16 | China | Tibet | HapⅡ-C | WheatUnion |
| 261 | S153 | XZbys_LangXianZheDa17 | China | Tibet | HapⅡ-C | WheatUnion |
| 262 | S154 | XZbys_LangXianZheDa25 | China | Tibet | HapⅡ-C | WheatUnion |
| 263 | S155 | XZbys_LongZiZheDa15 | China | Tibet | HapⅡ-C | WheatUnion |
| 264 | S156 | XZbys_LongZiZheDa22 | China | Tibet | HapⅡ-C | WheatUnion |
| 265 | S157 | XZbys_LongZiZheDa23 | China | Tibet | HapⅡ-C | WheatUnion |
| 266 | S158 | XZbys_LongZiZheDa26 | China | Tibet | HapⅡ-C | WheatUnion |
| 267 | S159 | XZbys_LongZiZheDa28 | China | Tibet | HapⅡ-C | WheatUnion |
| 268 | S160 | XZbys_LongZiZheDa29 | China | Tibet | HapⅡ-C | WheatUnion |
| 269 | S161 | XZbys_LongZiZheDa32 | China | Tibet | HapⅡ-C | WheatUnion |
| 270 | S163 | XZbys_LongZiZheDa48 | China | Tibet | HapⅡ-C | WheatUnion |
| 271 | S164 | XZbys_LongZiZheDa57 | China | Tibet | HapⅡ-C | WheatUnion |
| 272 | S165 | XZbys_LongZiZheDa62 | China | Tibet | HapⅡ-C | WheatUnion |
| 273 | S166 | XZbys_SangRiZheDa3 | China | Tibet | HapⅡ-C | WheatUnion |
| 274 | S167 | XZbys_SangRiZheDa4 | China | Tibet | HapⅡ-C | WheatUnion |
| 275 | S168 | XZbys_SangRiZheDa6 | China | Tibet | HapⅡ-C | WheatUnion |
| 276 | S169 | XZbys_SangRiZheDa7 | China | Tibet | HapⅡ-C | WheatUnion |
| 277 | S229 | YC_XZRiKaZe54 | China | Tibet | HapⅡ-C | WheatUnion |
| 278 | S245 | XZD_BianBaChunMai6 | China | Tibet | HapⅡ-C | WheatUnion |
| 279 | S247 | XZD_WuJiangZhuo | China | Tibet | HapⅡ-C | WheatUnion |
| 280 | S258 | XZbys_ChaYuZheDa1 | China | Tibet | HapⅡ-C | WheatUnion |
| 281 | S260 | XZbys_ChaYuZheDa6 | China | Tibet | HapⅡ-C | WheatUnion |
| 282 | S261 | XZbys_ChaYuZheDa8 | China | Tibet | HapⅡ-C | WheatUnion |
| 283 | S264 | XZbys_LangXianZheDa38 | China | Tibet | HapⅡ-C | WheatUnion |
| 284 | S265 | XZbys_LangXianZheDa47 | China | Tibet | HapⅡ-C | WheatUnion |
| 285 | S266 | XZbys_LongZiZheDa41 | China | Tibet | HapⅡ-C | WheatUnion |
| 286 | S267 | XZbys_LongZiZheDa42 | China | Tibet | HapⅡ-C | WheatUnion |
| 287 | S268 | XZbys_SangRiZheDa1 | China | Tibet | HapⅡ-C | WheatUnion |
| 288 | HCM | Hongchunmai | China | Xinjiang | HapⅠ-T | WheatUnion |
| 289 | S170 | DF_XJM1 | China | Xinjiang | HapⅠ-T | WheatUnion |
| 290 | TW037 | XM1222 | China | Xinjiang | HapⅡ-C | WheatUnion |
| 291 | TW039 | XM1379 | China | Xinjiang | HapⅡ-C | WheatUnion |
| 292 | KSBP | Kashibaipi | China | Xinjiang | HapⅡ-C | WheatUnion |
| 293 | S171 | DF_XJM20 | China | Xinjiang | HapⅡ-C | WheatUnion |
| 294 | S172 | DF_XJM29 | China | Xinjiang | HapⅡ-C | WheatUnion |
| 295 | S173 | DF_XJM81 | China | Xinjiang | HapⅡ-C | WheatUnion |
| 296 | S174 | DF_XJM90 | China | Xinjiang | HapⅡ-C | WheatUnion |
| 297 | S252 | DF_XJHongChunMai | China | Xinjiang | HapⅡ-C | WheatUnion |
| 298 | S253 | DF_XJHongDongMai | China | Xinjiang | HapⅡ-C | WheatUnion |
| 299 | S254 | DF_XJHongJinBaoYin | China | Xinjiang | HapⅡ-C | WheatUnion |
| 300 | TW030 | XM0929 | China | Yunnan | HapⅠ-T | WheatUnion |
| 301 | DXHKYM | Dianxihongkeyangmai | China | Yunnan | HapⅠ-T | WheatUnion |
| 302 | MP044 | ZhuShiMai | China | Yunnan | HapⅠ-T | WheatUnion |
| 303 | S6 | DF_YNTieKe6 | China | Yunnan | HapⅠ-T | WheatUnion |
| 304 | S56 | DF_YNTieKe2 | China | Yunnan | HapⅠ-T | WheatUnion |
| 305 | S57 | DF_YNTieKe19 | China | Yunnan | HapⅠ-T | WheatUnion |
| 306 | S176 | DF_YN109 | China | Yunnan | HapⅠ-T | WheatUnion |
| 307 | S177 | DF_YN156 | China | Yunnan | HapⅠ-T | WheatUnion |
| 308 | S178 | DF_YN241 | China | Yunnan | HapⅠ-T | WheatUnion |
| 309 | S179 | DF_YN411 | China | Yunnan | HapⅠ-T | WheatUnion |
| 310 | FMSY | Fengmai11 | China | Yunnan | HapⅡ-C | WheatUnion |
| 311 | S58 | DF_YNTieKe31 | China | Yunnan | HapⅡ-C | WheatUnion |
| 312 | S59 | DF_YNTieKe32 | China | Yunnan | HapⅡ-C | WheatUnion |

| **Table S8.** Information on genotypes containing the *TaUBC25* gene in the wheat diversity panel over decades. | | | | | |
| --- | --- | --- | --- | --- | --- |
| **Number** | **Resequencing cultivar ID** | **Accession** | **Years** | **Allele** | **Source** |
| 1 | MP021 | YanDai1817 | 1950 | HapⅠ-T | WheatUnion |
| 2 | S101 | DF_GSHongQiMai | 1904 | HapⅠ-T | WheatUnion |
| 3 | MP046 | BiMa1Hao | 1947 | HapⅠ-T | WheatUnion |
| 4 | MP049 | XiNong6028 | 1947 | HapⅠ-T | WheatUnion |
| 5 | S183 | DF_SHXMaZhaMai | 1950 | HapⅠ-T | WheatUnion |
| 6 | S213 | YCL_BiMa4 | 1947 | HapⅠ-T | WheatUnion |
| 7 | MP026 | ZhongGuoChun | 1932 | HapⅠ-T | WheatUnion |
| 8 | S225 | YCL_BeiJing8 | 1962 | HapⅡ-C | WheatUnion |
| 9 | MP056 | GanMai8Hao | 1964 | HapⅡ-C | WheatUnion |
| 10 | S227 | YCL_ShiJiaZhuang54 | 1961 | HapⅡ-C | WheatUnion |
| 11 | MP059 | JiNing3Hao | 1969 | HapⅡ-C | WheatUnion |
| 12 | S220 | YCL_TaiShan1 | 1969 | HapⅡ-C | WheatUnion |
| 13 | S214 | YCL_FengChan3 | 1971 | HapⅠ-T | WheatUnion |
| 14 | MP138 | KeChun14 | 1972 | HapⅡ-C | WheatUnion |
| 15 | HMM | Hongmangmai | 1979 | HapⅡ-C | WheatUnion |
| 16 | MP060 | BoAi7023 | 1970 | HapⅡ-C | WheatUnion |
| 17 | AMN | Aimengniu | 1970 | HapⅡ-C | WheatUnion |
| 18 | MP057 | JiNan9Hao | 1971 | HapⅡ-C | WheatUnion |
| 19 | S216 | YCL_Fan6 | 1971 | HapⅡ-C | WheatUnion |
| 20 | S273 | YC_Jing411 | 1987 | HapⅡ-C | WheatUnion |
| 21 | MP064 | YanShi4Hao | 1981 | HapⅡ-C | WheatUnion |
| 22 | MP071 | YuMai2Hao | 1983 | HapⅡ-C | WheatUnion |
| 23 | MP075 | YuMai7Hao | 1985 | HapⅡ-C | WheatUnion |
| 24 | MP083 | YuMai13 | 1989 | HapⅡ-C | WheatUnion |
| 25 | MP066 | NingChun4Hao (YongLiang4Hao) | 1981 | HapⅡ-C | WheatUnion |
| 26 | MP085 | ChuanMai22 | 1989 | HapⅡ-C | WheatUnion |
| 27 | KFEH | Fengkang2 | 1990 | HapⅡ-C | WheatUnion |
| 28 | S6554 | YC_JingDong6 | 1995 | HapⅡ-C | WheatUnion |
| 29 | MP081 | JiMai26 | 1991 | HapⅡ-C | WheatUnion |
| 30 | MP098 | JiMai38 | 1998 | HapⅡ-C | WheatUnion |
| 31 | MP099 | GaoYou503 | 1998 | HapⅡ-C | WheatUnion |
| 32 | MP109 | HanDan6172 | 1995 | HapⅡ-C | WheatUnion |
| 33 | MP141 | JiMai30 | 1996 | HapⅡ-C | WheatUnion |
| 34 | S64 | YC_Shi4185 | 1999 | HapⅡ-C | WheatUnion |
| 35 | PYEQ | Pingyang27 | 1994 | HapⅡ-C | WheatUnion |
| 36 | MP065 | BoNong3217 | 1990 | HapⅡ-C | WheatUnion |
| 37 | MP087 | YuMai17 | 1990 | HapⅡ-C | WheatUnion |
| 38 | MP091 | YuMai21Hao (ZhouMai9Hao) | 1994 | HapⅡ-C | WheatUnion |
| 39 | MP097 | YuMai41 | 1998 | HapⅡ-C | WheatUnion |
| 40 | S137 | YC_LuMai21 | 1996 | HapⅡ-C | WheatUnion |
| 41 | S223 | YCL_ZhengYin4 | 1993 | HapⅡ-C | WheatUnion |
| 42 | MP054 | XuZhou14 | 1990 | HapⅡ-C | WheatUnion |
| 43 | MP069 | ShanNong7859 | 1990 | HapⅡ-C | WheatUnion |
| 44 | MP077 | YangMai5Hao | 1990 | HapⅡ-C | WheatUnion |
| 45 | MP092 | YangMai158 | 1997 | HapⅡ-C | WheatUnion |
| 46 | S210 | YCL_SuiMai3 | 1990 | HapⅡ-C | WheatUnion |
| 47 | MP062 | TaiShan1Hao | 1990 | HapⅡ-C | WheatUnion |
| 48 | MP068 | ShanNongFu63 | 1991 | HapⅡ-C | WheatUnion |
| 49 | MP072 | LuMai1Hao (AiMengNiu) | 1990 | HapⅡ-C | WheatUnion |
| 50 | MP074 | JiNan13 | 1990 | HapⅡ-C | WheatUnion |
| 51 | MP086 | LuMai14 | 1990 | HapⅡ-C | WheatUnion |
| 52 | MP089 | LuMai15 | 1998 | HapⅡ-C | WheatUnion |
| 53 | MP096 | LuMai21 | 1996 | HapⅡ-C | WheatUnion |
| 54 | MP102 | JiNan16 | 1998 | HapⅡ-C | WheatUnion |
| 55 | MP106 | JiNan17 | 1999 | HapⅡ-C | WheatUnion |
| 56 | S215 | YCL_JiNan17 | 1999 | HapⅡ-C | WheatUnion |
| 57 | MP094 | JinMai47 | 1998 | HapⅡ-C | WheatUnion |
| 58 | MP063 | AiFeng3Hao | 1990 | HapⅡ-C | WheatUnion |
| 59 | MP067 | XiaoYan6Hao | 1990 | HapⅡ-C | WheatUnion |
| 60 | MP070 | MianYang11 | 1990 | HapⅡ-C | WheatUnion |
| 61 | MP073 | MianYang15 | 1991 | HapⅡ-C | WheatUnion |
| 62 | MP093 | MianYang26Hao | 1998 | HapⅡ-C | WheatUnion |
| 63 | KSBP | Kashibaipi | 1990 | HapⅡ-C | WheatUnion |
| 64 | MP118 | LunXuan987 | 2003 | HapⅡ-C | WheatUnion |
| 65 | S271 | YC_LX987 | 2003 | HapⅡ-C | WheatUnion |
| 66 | YM07 | Nongda3753 | 2005 | HapⅡ-C | WheatUnion |
| 67 | MP104 | GaoCheng8901 | 2000 | HapⅡ-C | WheatUnion |
| 68 | MP111 | DanJiaZhuang8Hao | 2007 | HapⅡ-C | WheatUnion |
| 69 | MP120 | HengGuan35 | 2006 | HapⅡ-C | WheatUnion |
| 70 | MP145 | ZhongYou9507 | 2001 | HapⅡ-C | WheatUnion |
| 71 | MP103 | WenMai6Hao (YuMai49) | 2000 | HapⅡ-C | WheatUnion |
| 72 | MP110 | ZhengMai9023 | 2003 | HapⅡ-C | WheatUnion |
| 73 | MP113 | ZhouMai16 | 2003 | HapⅡ-C | WheatUnion |
| 74 | MP117 | YanZhan4110 | 2002 | HapⅡ-C | WheatUnion |
| 75 | MP124 | ZhengMai366 | 2005 | HapⅡ-C | WheatUnion |
| 76 | MP125 | BoNongAK58 | 2003 | HapⅡ-C | WheatUnion |
| 77 | MP128 | ZhouMai22Hao | 2007 | HapⅡ-C | WheatUnion |
| 78 | MP131 | XinMai26 | 2010 | HapⅡ-C | WheatUnion |
| 79 | MP132 | FengDeCunMai5Hao | 2014 | HapⅡ-C | WheatUnion |
| 80 | MP134 | ZhongMai66 | 2014 | HapⅡ-C | WheatUnion |
| 81 | MP135 | BoNong201 | 2014 | HapⅡ-C | WheatUnion |
| 82 | MP136 | BoNong4199 | 2017 | HapⅡ-C | WheatUnion |
| 83 | S139 | YC_YuMai18 | 2003 | HapⅡ-C | WheatUnion |
| 84 | S218 | YCL_YanZhan1 | 2000 | HapⅡ-C | WheatUnion |
| 85 | PH135 | Zhongmai875 | 2020 | HapⅡ-C | WheatUnion |
| 86 | MP112 | HuaiMai20 | 2003 | HapⅡ-C | WheatUnion |
| 87 | MP133 | XuMai35 | 2015 | HapⅡ-C | WheatUnion |
| 88 | MP108 | JiMai19 | 2001 | HapⅡ-C | WheatUnion |
| 89 | MP114 | YanNong21Hao | 2004 | HapⅡ-C | WheatUnion |
| 90 | MP115 | JiMai20 | 2003 | HapⅡ-C | WheatUnion |
| 91 | MP127 | JiMai22 | 2010 | HapⅡ-C | WheatUnion |
| 92 | MP130 | LiangXing66 | 2008 | HapⅡ-C | WheatUnion |
| 93 | S136 | YC_YanNong15 | 2000 | HapⅡ-C | WheatUnion |
| 94 | S272 | YC_JiMai22 | 2006 | HapⅡ-C | WheatUnion |
| 95 | YM05 | Liangxing99 | 2006 | HapⅡ-C | WheatUnion |
| 96 | MP129 | ZhongMai175 | 2011 | HapⅡ-C | WheatUnion |
| 97 | S209 | YCL_ChangZhi6406 | 2002 | HapⅡ-C | WheatUnion |
| 98 | MP095 | XiNong881 | 2002 | HapⅡ-C | WheatUnion |
| 99 | C45 | VA_China5 | 2004 | HapⅡ-C | WheatUnion |
